# Supplementary material for: Spatiotemporal gene expression and cellular dynamics of the developing human heart
Source: Nat Genet. 2025 Oct 29;57(11):2756–71. doi: 10.1038/s41588-025-02352-6 (PMC12597827; doi:10.1038/s41588-025-02352-6)
Supplement: Supplementary file 1 — Supplementary Figs. 1–19, Supplementary Methods, Supplementary Discussion 1 and 2, Supplementary References. [file 41588_2025_2352_MOESM1_ESM.pdf]

nature genetics

Supplementary information https://doi.org/10.1038/s41588-025-02352-6

Spatiotemporal gene expression and cellular dynamics of the developing human heart

In the format provided by the authors and unedited

SUPPLEMENTARY FIGURES

Supplementary Figure 1. Molecular Region Identification Based on Spatially Aware Clustering of Visium Spots with Banksy. A. Temporal evolution of Banksy spatial regions and Seurat-based spatial clusters, presented in 6, 8, 10 and 12 pcw heart sections. HE–hematoxylin-eosin; the scale bar represents 1 mm. **B.** UMAP of Visium spots from Seurat clustering, embedded according to 14 spatial regions determined by Banksy. Major cardiac compartments are demarcated by dashed lines. **C.** Dot plot depicting the top 5 DEGs (log2FC > 0, p_val < 0.05) across 14 Banksy regions. **D.** Spot transfer matrix between the 23 Seurat-based spatial clusters and the 14 Banksy regions. **E.** Distribution of selected markers between 14 spatial Banksy regions (gene set consistent with Figure 1D). In C and E panels: Avg. exp.–average expression, Pct. exp.–percent of expressing cells.

Supplementary Figure 2. Molecular Markers and Temporal Transitions of Major Cardiac Cell Populations. A. Dot plot depicting the top 5 DEGs (log2FC > 0, p_val < 0.05) between the 31 coarse-grained single-cell clusters. Avg. exp.–average expression, Pct. exp.–percent of expressing cells. **B.** UMAPs illustrating size changes of coarse-grained clusters across four developmental age groups (5.5-6, 7-8, 9-11, and 12-14 pcw, each age group downsampled to n=8,742 cells). Dashed lines mark clusters with min. 2x increase (red) or decrease (blue) in proportion compared to the total number of cells between the 5.5-6 and 12-14 pcw age groups.

Supplementary Figure 3. Molecular Analysis of Mural Cell States. A. Coarse-grained clusters corresponding to pericytes and smooth muscle cells of the outflow tract, great vessels and coronary arteries, displayed on the UMAP of the integrated single-cell RNA-sequencing dataset. **B.** Top 5 DEGs (log2FC > 0, p_val < 0.05) between time-resolved (5.5-6 pcw, 7-8 pcw, 9-11 pcw and 12-14 pcw) subpopulations across all coarse-grained clusters (left), highlighting highly enriched genes with relevant temporal trends in mural cell populations (OFT_SMC, CA_SMC, PC) (right). **C.** Top 10 DEGs (log2FC > 0, p_val < 0.05) between the OFT_SMC, CA_SMC and PC clusters. **D.** Dot plot displaying enrichment of *NOTCH3* and *JAG1* in coarse-grained clusters corresponding to cellular components of the coronary and great arteries. **E.** Spatial expression patterns of *NOTCH3* and *JAG1* in 8 pcw, 10 pcw, and 12 pcw heart sections, outlining the coronary (solid rectangles) and great arteries (dashed rectangles) as predominant locations for cell-cell communication mediated by these molecules. HE–hematoxylin-eosin; the scale bars represent 1 mm. **F.** Dot plot displaying shared markers between the Peric_MC^{fg} and mural mesenchymal cell states. In B-D and F panels: Avg. exp.–average expression, Pct. exp.–percent of expressing cells.

Supplementary Figure 4. Molecular Analysis of Blood-Related Cell States. A. Coarse-grained clusters corresponding to myeloid (MyC), lymphoid (LyC) and red blood cells (HL_excl_1 and HL_excl_4), displayed on the UMAP of the integrated single-cell RNA-sequencing dataset. **B.** Dot plot displaying differential enrichment of platelet, granulocyte and embryonic and mature red blood cell markers in the HL_excl_1 and HL_excl_4 clusters. Avg. exp.–average expression, Pct. exp.–percent of expressing cells. **C.** Feature plots presenting the distribution of selected marker genes in the MyC (upper) and LyC (lower) populations. **D.** Niche graph displaying the closest cellular neighbors of MyCs and LyCs in our dataset, determined by cell state mapping of fine-grained single-cell clusters in the Visium dataset. **E.** Enrichment of LyCs (green) and LECs (red) outline lymphoid tissue regions (solid rectangles) in the adventitia of the great arteries in a 12 pcw tissue section, supported by the spatial distribution of lymphocyte (*LCP1*, *NKG7*, *CD52*), lymphatic endothelial cell (*CCL21*, *LYVE1*) and lymphoid stromal cell (*CCL19*) markers. HE–hematoxylin-eosin; the scale bar represents 1 mm.

Supplementary Figure 5. Transcriptional Heterogeneity of Epicardium-Related Cell States. A. Coarse-grained clusters corresponding to epicardial (EpC) and epicardium-derived progenitor cells (EPDC), displayed on the UMAP of the integrated single-cell RNA-sequencing dataset. **B.** Dot plot showing the relative enrichment of epicardial cell and EPDC markers across the EpC and fine-grained non-mural mesenchymal cell-fibroblast cell states. **C.** Top 10 DEGs (log2FC > 0, p_val < 0.05) between EpC, EPDC_1 and EPDC_2 fine-grained cell states. **D.** Top 5 DEGs (log2FC > 0, p_val < 0.05) between time-resolved (5.5-6 pcw, 7-8 pcw, 9-11 pcw and 12-14 pcw) subpopulations across all coarse-grained clusters (left), highlighting highly enriched genes with relevant temporal trends in the EpC and coarse-grained EPDC populations (right). **E.** Dot plot visualizing the relative enrichment of epicardium-related transcription factors *WT1*, *TBX18* and *TCF21,* across all fine-grained cell states. In B-E panels: Avg. exp.–average expression, Pct. exp.–percent of expressing cells.

Supplementary Figure 6. Temporal Gene Expression Changes in Coarse-Grained Endothelial Cell and Mesenchymal Cell-Fibroblast Clusters. A. Top 5 DEGs (log2FC > 0, p_val < 0.05) between time-resolved (5.5-6 pcw, 7-8 pcw, 9-11 pcw and 12-14 pcw) subpopulations across all coarse-grained clusters, highlighting highly enriched genes with relevant temporal trends in the single-cell dataset. **B.** DEGs in time-resolved coarse-grained clusters representing vascular (MacroVasc_EC, MicroVasc_EC) (A'), endocardium-related (Endoc_EC, EndocCush_EC) (B') and lymphatic endothelial cell (LEC) (C') populations. **C.** DEGs in time-resolved coarse-grained clusters representing interstitial fibroblasts (Int_FB), pericyte-like mesenchymal cells (Peric_MC) (D'), and annulus fibrosus fibroblasts (AnnFibr_FB) (E'). In B-C panels: Avg. exp.–average expression, Pct. exp.–percent of expressing cells.

Supplementary Figure 7. Assessment of Spatiotemporal Transcriptomic Patterns in the Cardiac Valves, Outflow Tract and Great Arteries. A. Top 5 DEGs (log2FC > 0, p_val < 0.05) between time-resolved (5.5-6 pcw, 7-8 pcw, 9-11 pcw and 12-14 pcw) subpopulations across all coarse-grained clusters (left), highlighting highly enriched genes with relevant temporal trends in populations with characteristic localization in the cardiac valves (Valve_MC) (middle), and outflow tract and great arteries (OFT_FB, OFT_SMC) (right). Rectangles mark genes up- (red) and downregulated (blue) over time, spatially plotted in panel B and C. Avg. exp.–average expression, Pct. exp.–percent of expressing cells. **B.** Spatial expression patterns of genes showing decreasing (*EFNA5*, *FOXP2*, *PRDM6*) and increasing (*PI15*, *DLK1*) expression over time within the region of the outflow tract and great arteries (solid squares), displayed in 6 pcw, 8 pcw and 10 pcw tissue sections. **C.** Spatial expression patterns of genes showing decreasing (*SEMA3D*) and increasing (*COL12A1*) expression over time within the region of the semilunar valves (solid squares), displayed in 6 pcw and 10 pcw tissue sections. In B-C panels: HE–hematoxylin-eosin; the scale bar represents 1 mm.

Supplementary Figure 8. Pathological Gene Panel Enrichment across Coarse- (A) and Fine-Grained (B) Cell States.

Supplementary Figure 9. Marker Distribution of Pacemaker and Conductive Cardiomyocyte States. A. Spatial expression pattern of selected marker genes enriched in SAN_CMs and AVN_CMs (compared to other fine-grained cardiomyocyte states) in a 6.5 pcw heart section, detected by *in situ* sequencing (ISS). The displayed gene panel is consistent with the one included in Fig. 3D. The scale bar represents 1 mm. **B.** Spatial expression pattern of marker genes enriched in the PF_CMs, TsPF_CMs and AVB-BB_CMs (compared to other fine-grained cardiomyocyte states) in a 9 pcw heart section, detected by ISS. The displayed gene panel is consistent with the one included in Fig. 3E. The scale bar represents 1 mm. **C.** Relative enrichment of genes visualized in panel A and B across all fine-grained cell states. Avg. exp.–average expression, Pct. exp.–percent of expressing cells.

Supplementary Figure 10. Differentiation Marker Expression in Innervation-Related Cell States. Expression patterns of selected genes associated with glial, neuronal, and chromaffin cell differentiation are presented within the PCA embedding of the innervation-related cell subset.

Supplementary Figure 11. Spatial Arrangement and Marker Profiles of Endothelial Cell States. A. Spatial distribution of endothelial cell states in the great arteries and coronary vasculature, including Art_EC_1 (red), Art_EC_2 (green) cells and Venul_ECs (blue) (upper), and Arteriol_ECs (red), Cap_EC_1 (green) and Cap_EC_2 (blue) cells (lower), presented in a 10 pcw heart section. Arrows–coronary arteries, arrowheads–coronary veins; the scale bars represent 1 mm in the main and 0.3 mm

in the zoom-in panel. **B.** Spatial mapping of Cap_EC_1 (red) and Cap_EC_2 (green) cells in 6, 8, 10, and 12 pcw heart sections (left). Dot plot displaying the top 10 DEGs (log2FC > 0, p_val < 0.05) between the Cap_EC_1 and Cap_EC_2 clusters (right). **C.** Spatial mapping of Endoc_EC_1 (red) and Endoc_EC_2 (green) (upper), and Endoc_EC_3 (red) and Endoc_EC_4 (green) (lower) cells in 6, 8, 10, and 12 pcw heart sections (left). Dot plot displaying the top 10 DEGs (log2FC > 0, p_val < 0.05) between the Endoc_EC_1, Endoc_EC_2, Endoc_EC_3 and Endoc_EC_4 clusters (right). **D.** Spatial mapping of OF_VECs (red) and IF_VECs (green) in an 8 pcw heart section, demonstrating their distinct localization on opposite sides of the atrioventricular valves. The scale bars represent 1 mm in the main and 0.5 mm in the zoom-in panels. In A and D panels: la–left atrium, ra–right atrium, lv–left ventricle, rv–right ventricle, ao–aorta, avg–atrioventricular groove, avv–atrioventricular valve, arrowheads–inflow side, asterisks–outflow side, HE–hematoxylin-eosin. In B-C panels: Avg. exp.–average expression, Pct. exp.–percent of expressing cells.

Supplementary Figure 12. Quality Metrics of Heart Sections Included in the Spatial Transcriptomics Dataset. A. Hematoxylin-eosin images of the 38 embryonic and fetal heart sections included in the Visium dataset. Seventeen sections selected for spatial clustering are marked by red font. The scale bar represents 1 mm. **B.** Sex distribution of donors in the Visium dataset. Out of 16 hearts, 8 were collected from male and 8 from female donors, determined by Y-chromosome-linked *KDM5D* gene expression in representative tissue sections. **C.** Age distribution of donors and quality metrics of the Visium dataset. Violin plots represent nCount and nFeature values across all sections included in the Visium dataset. **D.** Spatiotemporal quality assessment of Visium sections. Spatial feature plots of nFeature and nCount values are presented in 6, 8, 10 and 12 pcw heart sections. **E**. Heart sections included in the ISS dataset. Eight sections collected from 4 hearts, representing 6.5, 8.5, 9 and 11.5 pcw developmental stages, were analyzed by ISS, targeting 150 genes. High ISS signal densities for *MYL2* (in blue), *MYH6* (in purple), *FBLN5* (in green) and *TBX18* (in red) transcripts outline the position of ventricles, atria, great arteries, and epicardium, respectively.

Supplementary Figure 13. Sample Distribution Overview and Data Integration between Experimental Samples in the Visium Dataset. A. Temporal distribution of heart samples included in the single-cell RNA-sequencing (scRNAseq) and spatially resolved transcriptomics datasets, with donor numbers by postconceptional week (pcw) shown in blue, orange and green circles per used technology. Dashed rectangles outline samples grouped for age-resolved assessment of cluster distribution. **B.** Distribution of independent samples across spatial clusters in the integrated (n=25,208) (upper) and time-resolved (6-7 pcw, 8-9 pcw, 10-12 pcw; downsampled to n=2,649 spots per group) Visium spatial transcriptomics dataset (lower).

Supplementary Figure 14. Alignment Analysis between Sections in the Visium and ISS Datasets. A. Per section distribution of Banksy-based spatial regions across the 17 Visium sections included in the clustering analysis. **B.** Per section distribution of Seurat-based spatial clusters across the 17 Visium sections included in the clustering analysis. **C.** Per section distribution of coarse-grained single-cell clusters, calculated by stereoscope, across the entire Visium dataset (38 sections). **D.** Correlation between detected transcript numbers of individual targets in technical replicates within the ISS dataset.

Supplementary Figure 15. Comparison of Maximal Gene Expression Detected in the ISS and scRNAseq datasets. A. Scatter plot showing maximal expression values per gene based on shared coarse-grained cell clusters in the ISS and scRNAseq datasets. **B.** Box plot showing maximal expression values per gene in coarse-grained cell clusters shared between the ISS and scRNAseq datasets, with the mean values calculated for the entire gene panel for summative quantification. The horizontal center line indicates the median, the box edges represent the upper and lower quartiles, and the whiskers extend to 1.5 times the interquartile range for each experimental setting.

Supplementary Figure 16. Quality Metrics of Heart Samples Included in the scRNAseq Dataset. A. Sex distribution of donors in the scRNAseq dataset. Out of 16 hearts, 9 were collected from female and 6 from male donors, determined by X chromosome inactivation-related *XIST* gene expression in the samples. **B.** Age distribution of donors and quality metrics of the scRNAseq dataset. Bar graphs illustrate nCount, nFeature, percentage of mitochondrial, ribosomal and hemoglobin transcripts in single cells by sequencing sample. Horizontal dashed lines correspond to values used for filtering high quality cells. **C.** Original UMAP of coarse-grained single-cell clusters. **D.** Sample distribution across coarse-grained single-cell clusters. **E.** Quality metrics across coarse-grained single-cell clusters. UMAPs visualize nCount, nFeature, and percentage of mitochondrial and ribosomal transcripts across the entire scRNAseq dataset. **F.** Quality metrics used for coarse-grained cluster exclusion. Violin plots visualizing nFeature and *HBA1* gene expression across all coarse-grained clusters were used to identify clusters with low quality or red blood cell contamination (red rectangles), excluded from downstream analysis.

Supplementary Figure 17. Data Integration between Experimental Samples in the scRNAseq Dataset. Distribution of independent samples across coarse-grained single-cell clusters in the integrated (n=73,946) (left) and time-resolved (5.5-6 pcw, 7-8 pcw, 9-11 pcw, 12-14 pcw; downsampled to 8,742 cells per group) scRNAseq dataset (right).

Supplementary Figure 18. Exclusion Criteria for Fine-Grained Single-Cell Clusters. A. Original UMAPs of all fine-grained single-cell clusters in the cardiomyocyte (CM, left), endothelial cell (EC,

middle) and mesenchymal cell-fibroblast (MC-FB, right) subsets. **B.** Violin plots of nFeature values in all fine-grained clusters of the CM (top), EC (middle) and MC-FB (bottom) subsets. Dashed red rectangles outline clusters with potential doublet contamination, and solid red rectangles mark clusters with low quality. **C.** Violin plots of *HBA1* expression in all fine-grained clusters of the CM (top), EC (middle) and MC-FB (bottom) subsets. Red rectangles highlight clusters with potential red blood cell contamination. **D.** Dot plots displaying relative enrichment of consensus cell type markers across the original fine-grained clusters in the CM (left), EC (middle) and MC-FB (right) subsets. Dashed rectangles highlight consensus markers of cardiomyocytes, endothelial cells, and fibroblasts. Gray backdrops mark clusters excluded from downstream analysis, based on low quality, potential doublet contamination, or lack of consensus marker expression of the relevant cell types. **E.** UMAP of all fine-grained clusters in the innervation-related cell subset (left). Dot plot illustrating relative enrichment of consensus markers of glial, neuronal, and chromaffin cell differentiation across all fine-grained innervation-related clusters (right). Gray backdrops mark clusters excluded from downstream analysis, based on lacking or low expression of genes included in the panel. In D-E panels: Avg. exp.–average expression, Pct. exp.–percent of expressing cells.

Supplementary Figure 19. Cluster Trees of Cellular Subsets. Hierarchical cluster trees of single-cell subsets (**A.** Cardiomyocytes; **B.** Innervation-related cell states; **C.** Endothelial cells; **D.** Mesenchymal cells-fibroblasts) obtained by clustering with increasing resolution, highlighting varying levels of transcriptional similarities between fine-grained cell states.

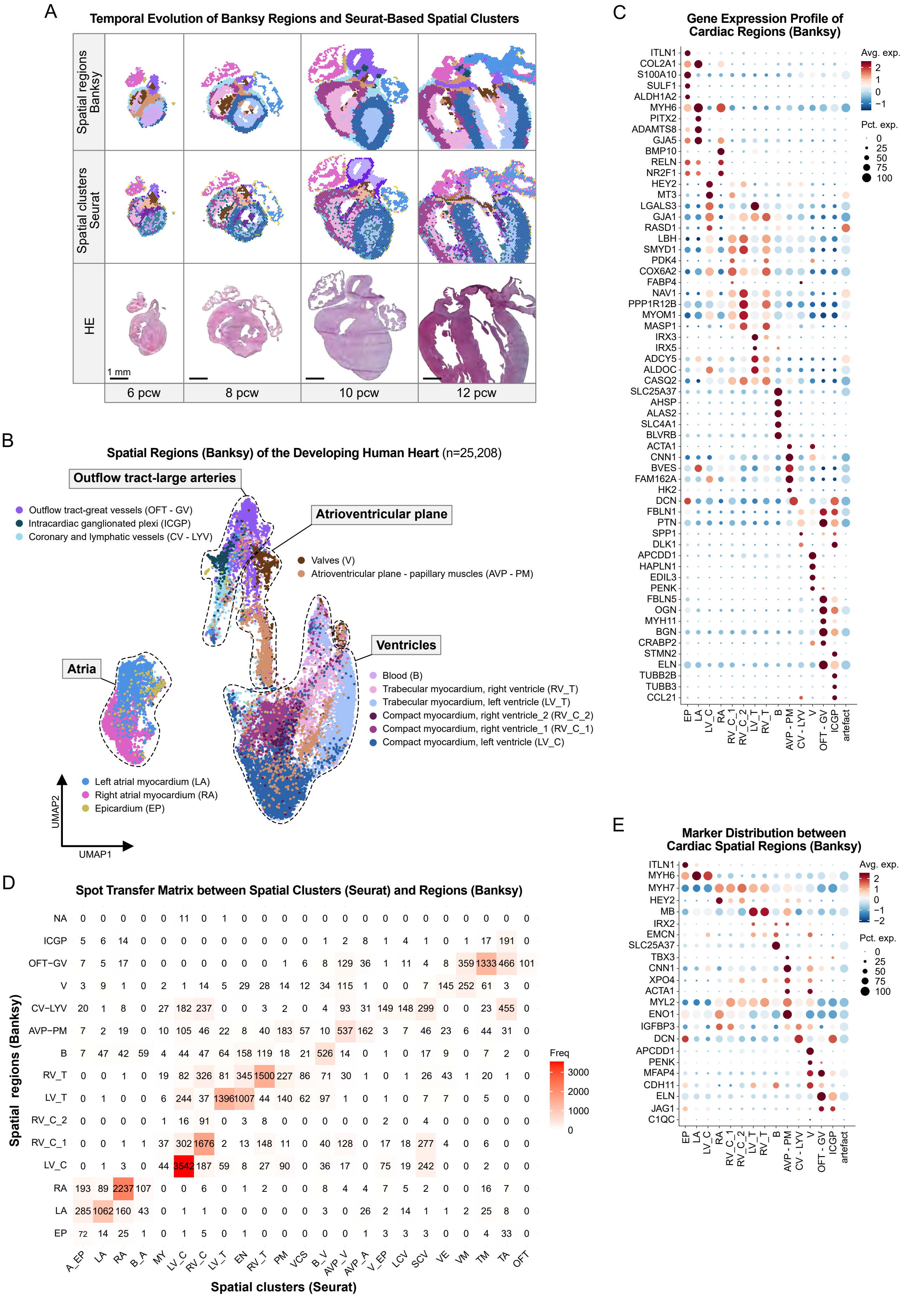

Supplementary Figure 1

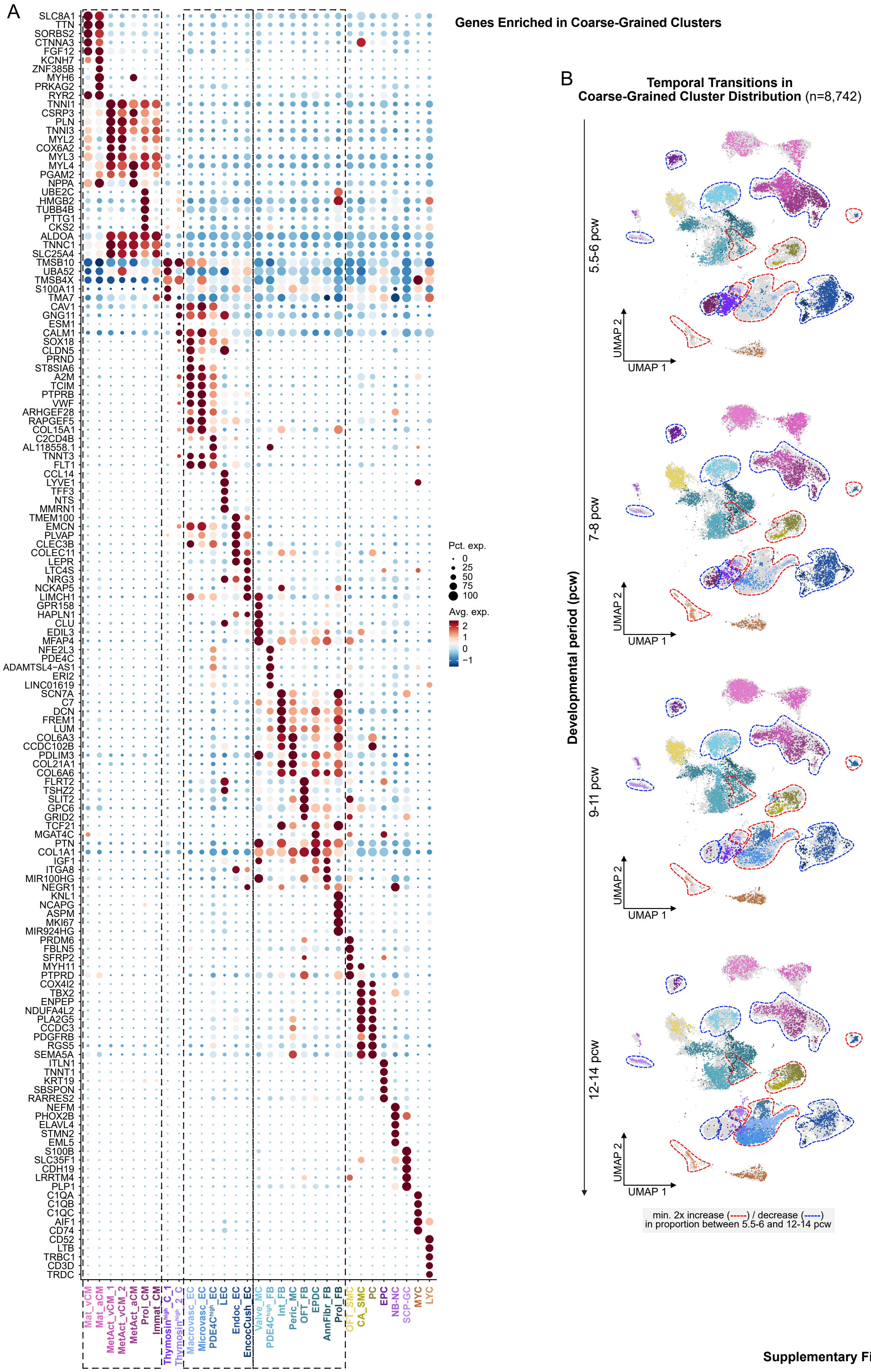

Supplementary Figure 2

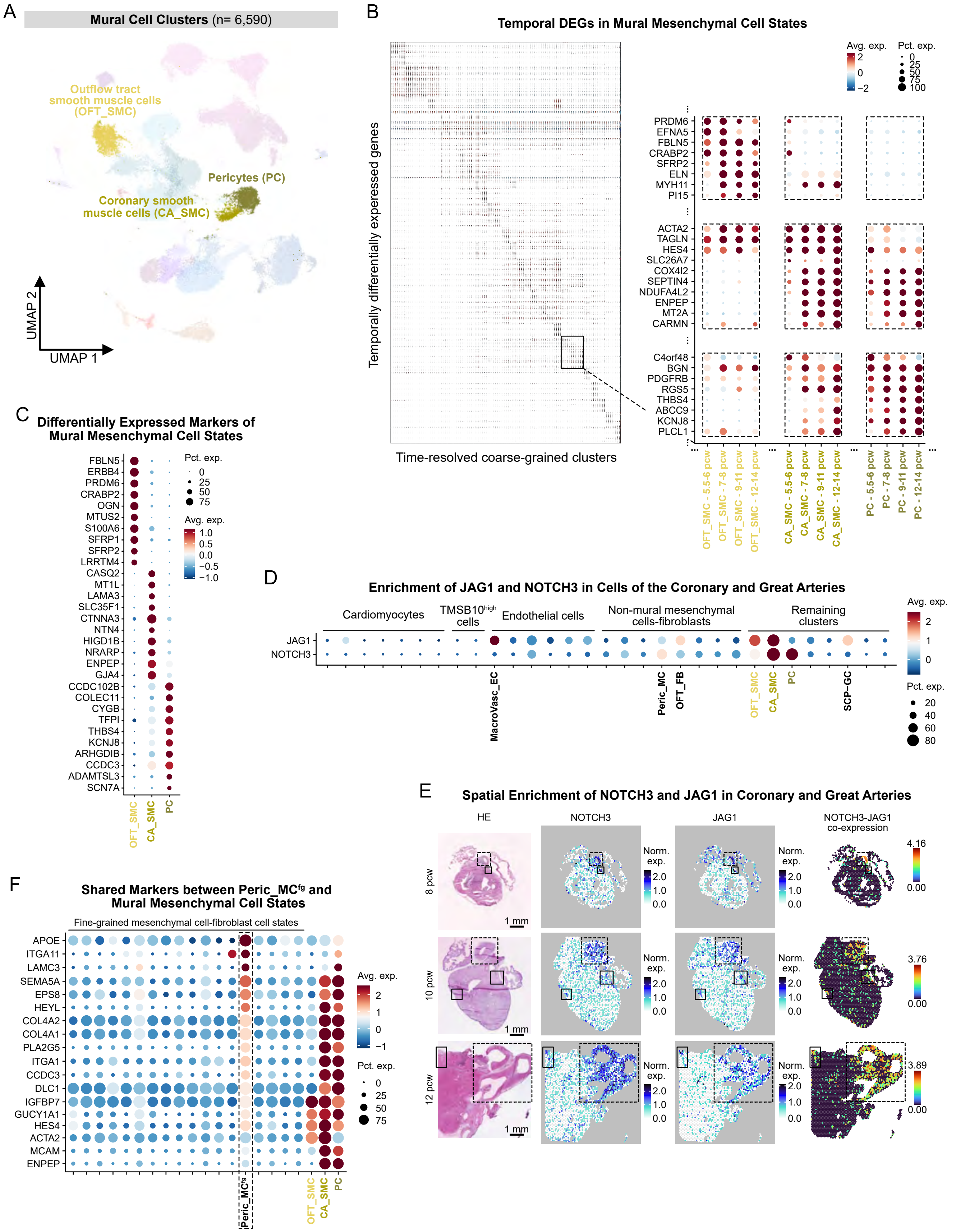

Supplementary Figure 3

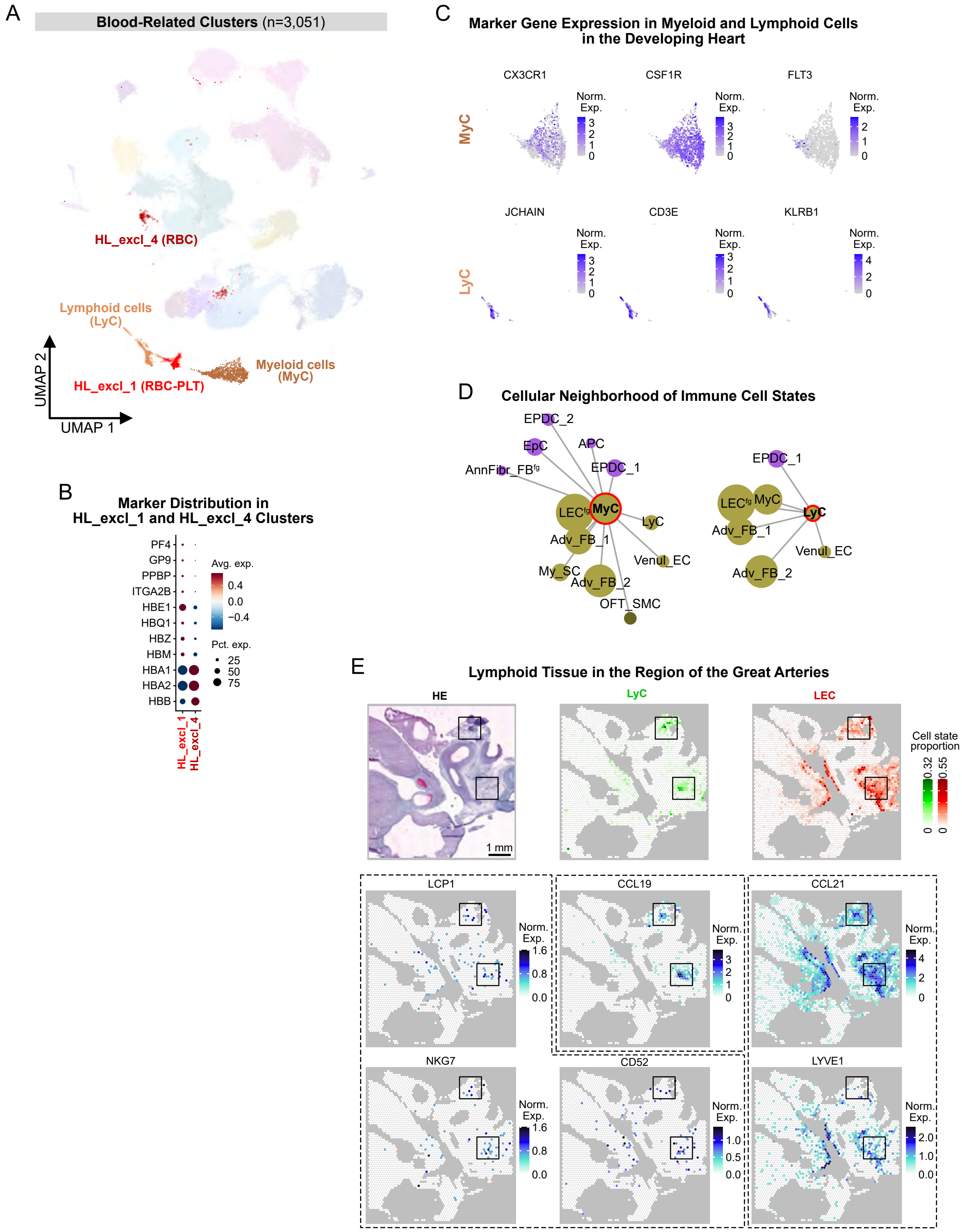

Supplementary Figure 4

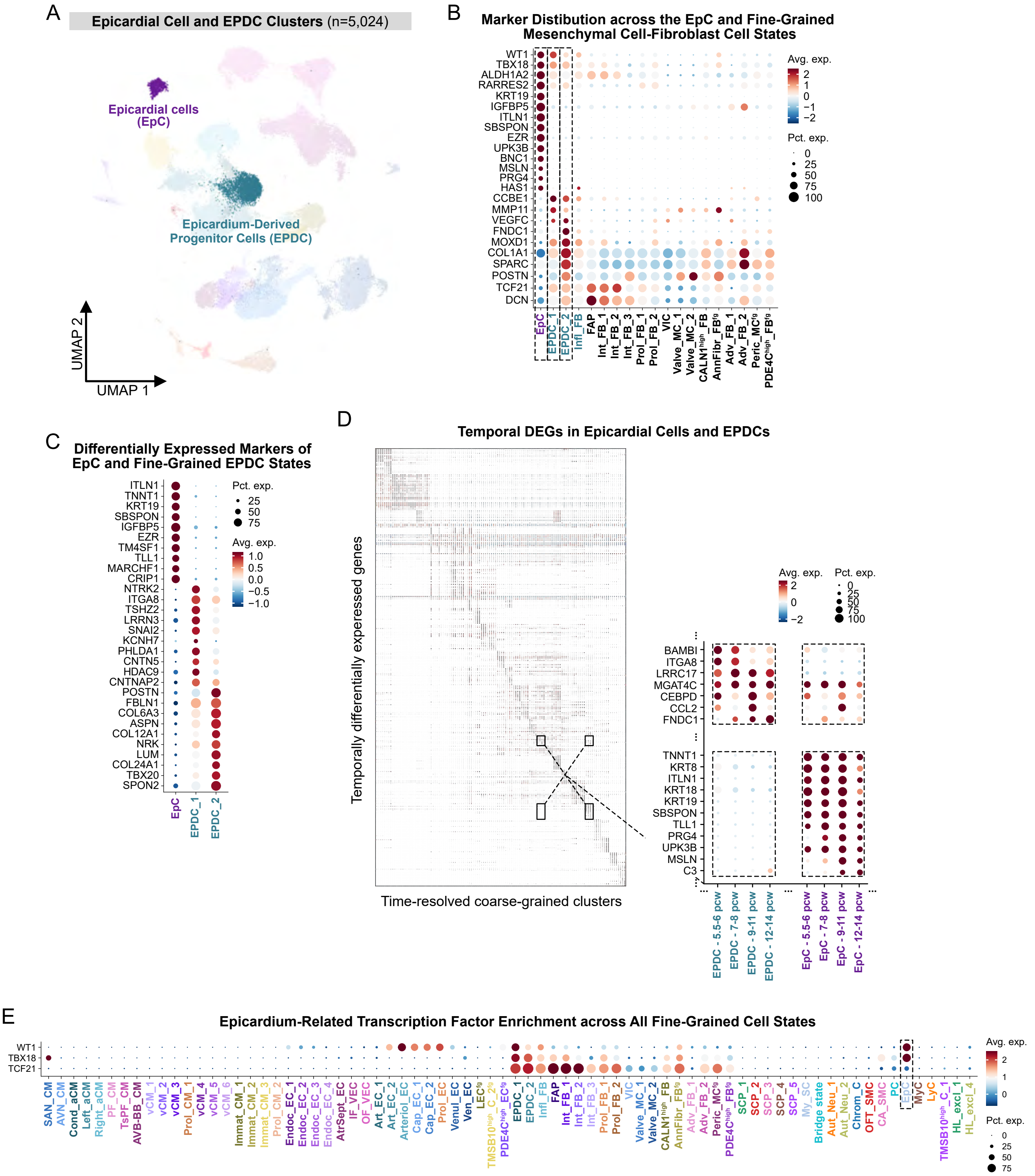

Supplementary Figure 5

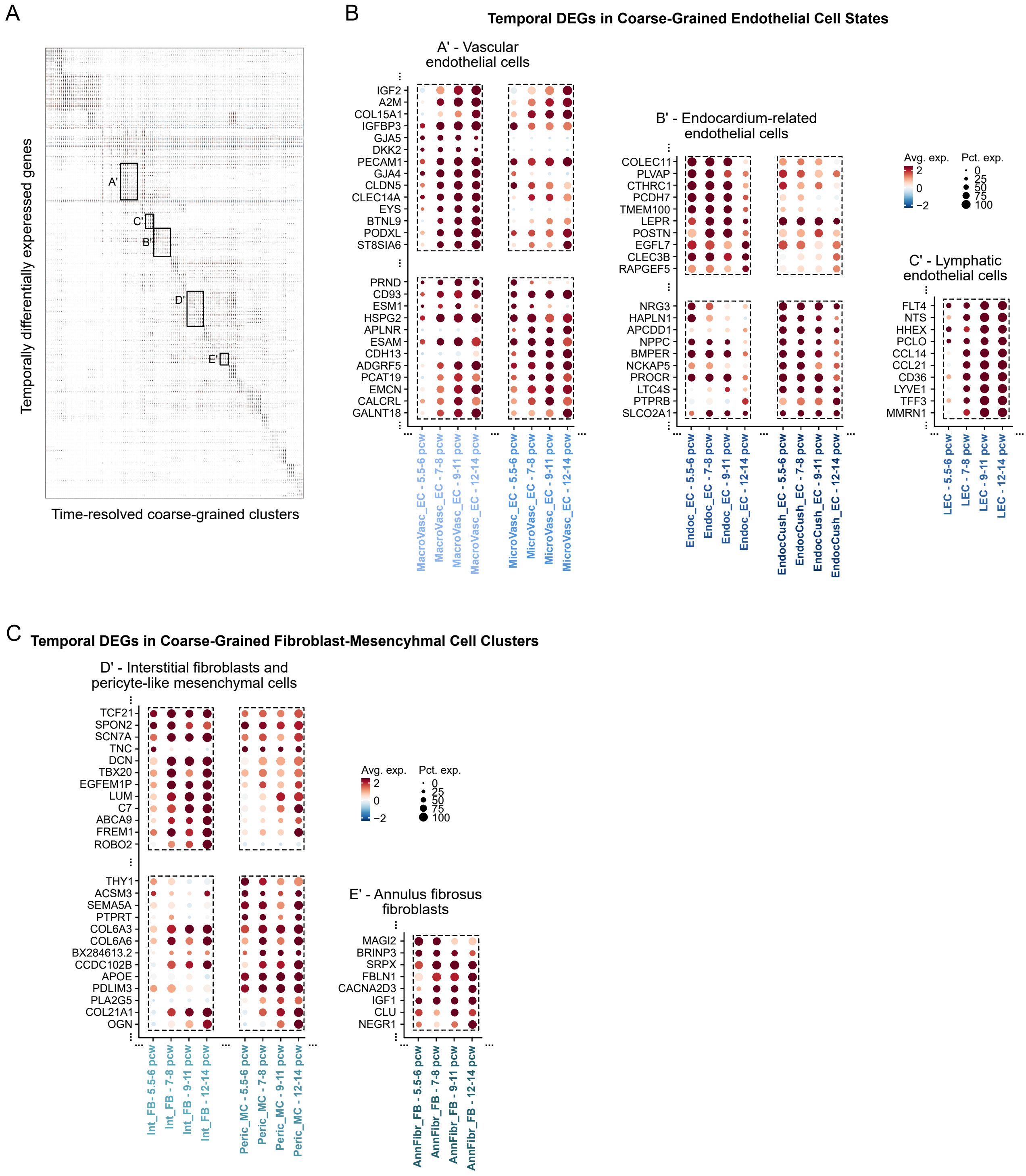

Supplementary Figure 6

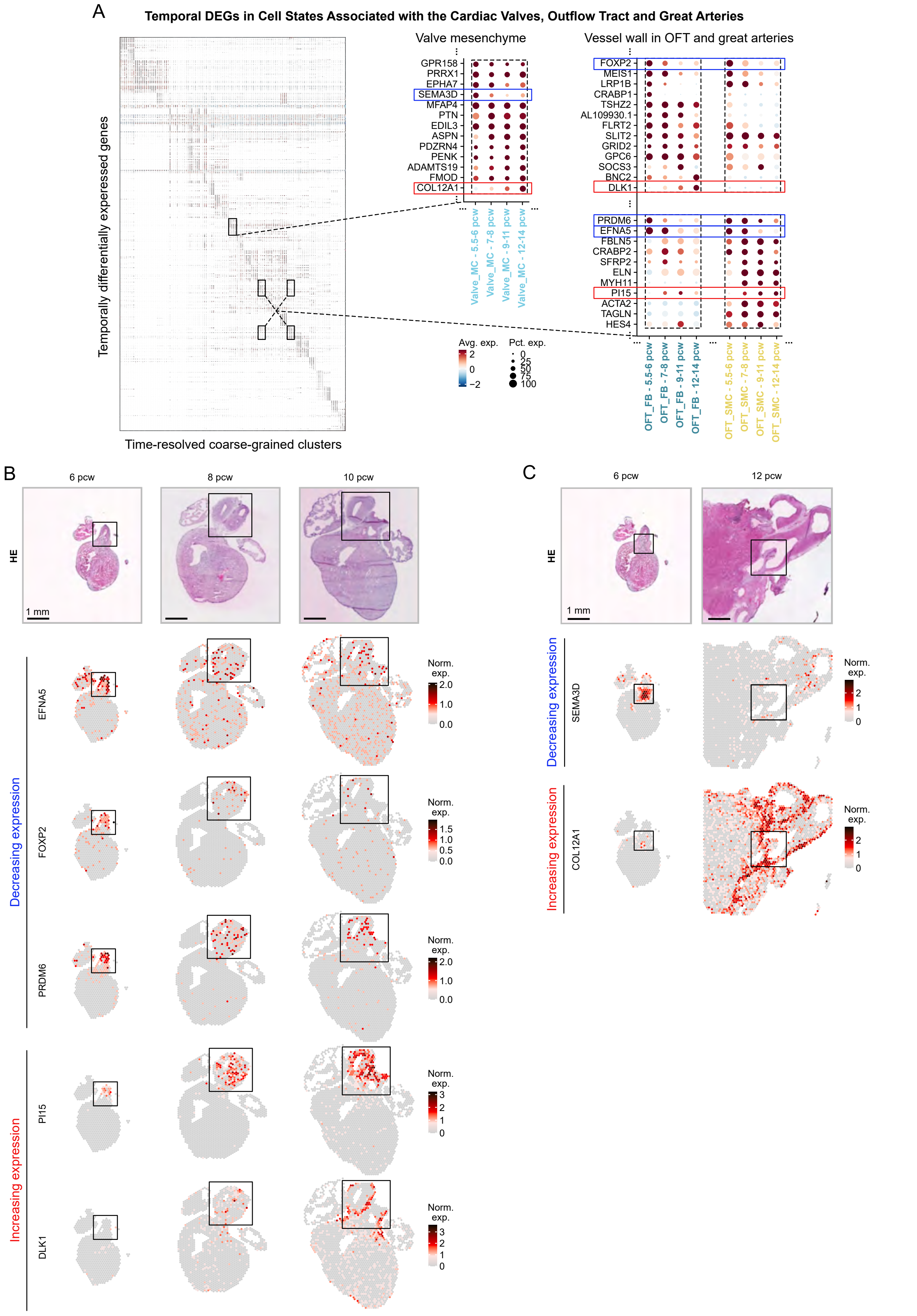

Supplementary Figure 7

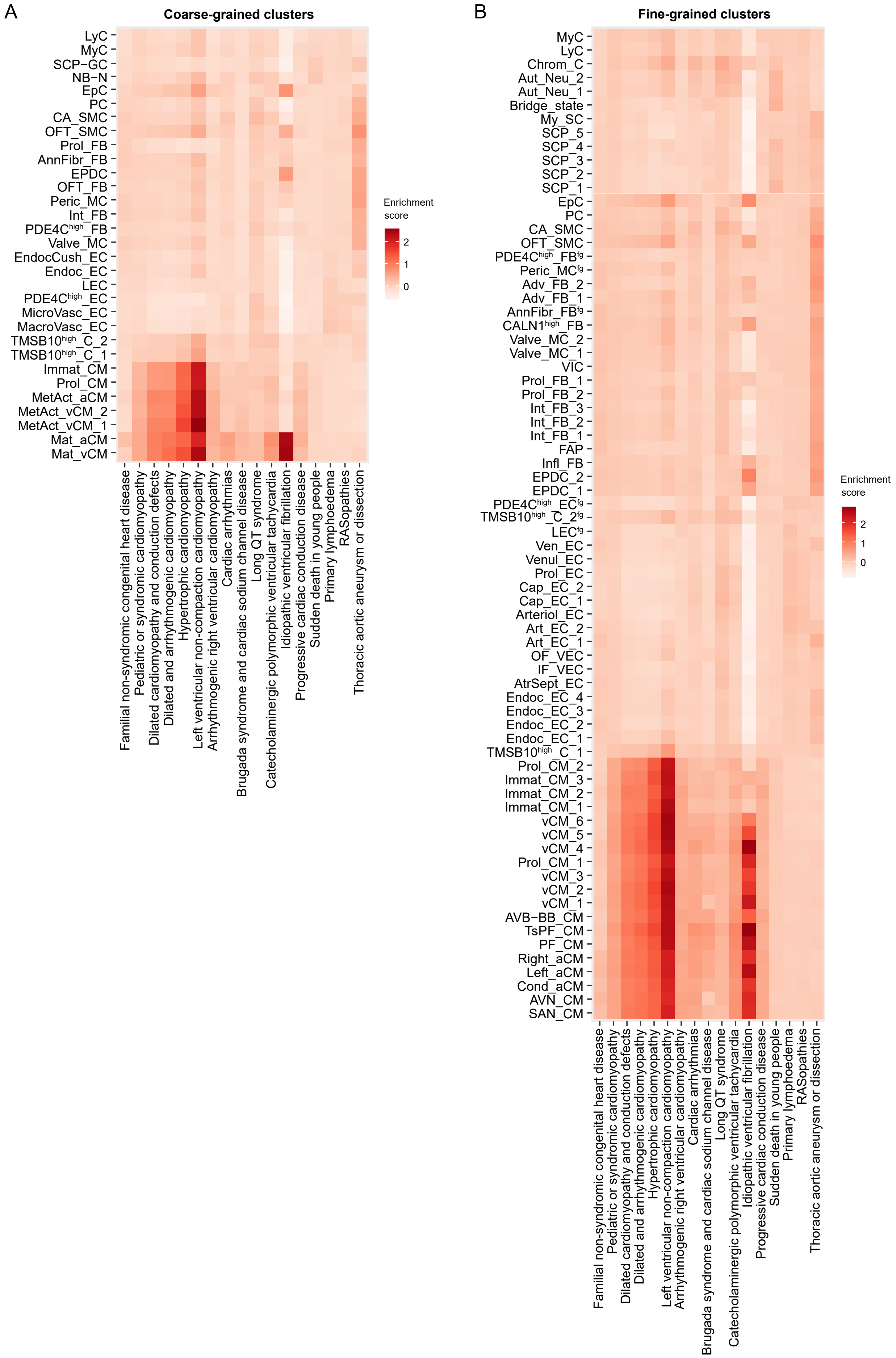

Supplementary Figure 8

A

Spatial Distribution of Markers of APCS Cardiomyocyte States

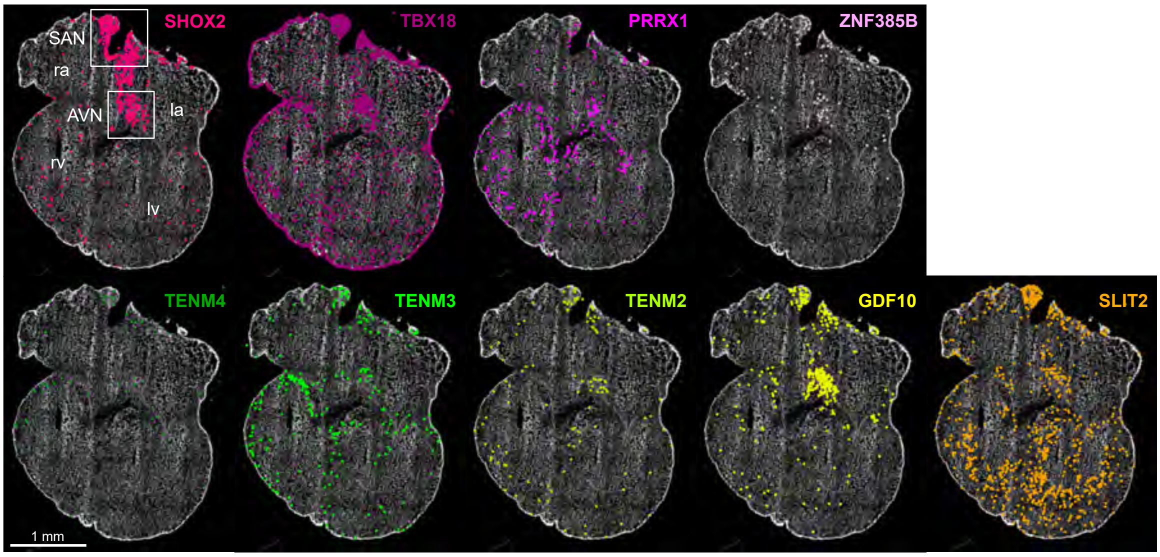

B

Spatial Distribution of Markers of VCS Cardiomyocyte States

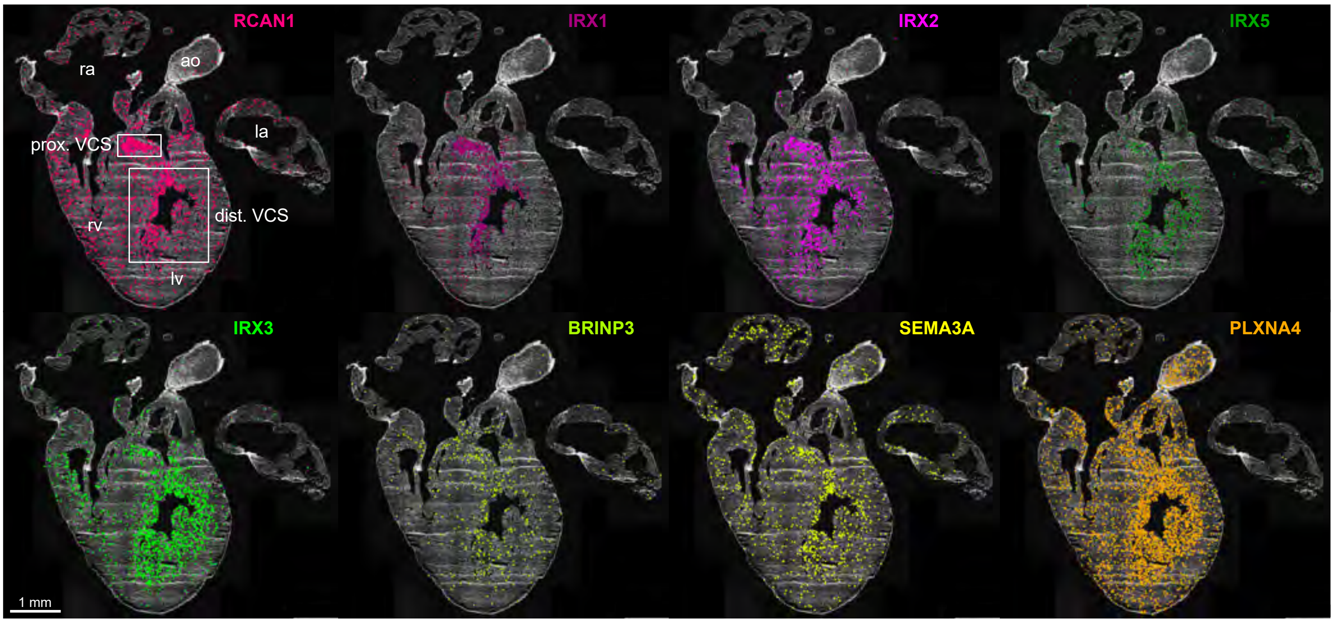

C

Relative Enrichment of CPCS Cardiomyocyte State Markers across All Fine-Grained Single-Cell Clusters

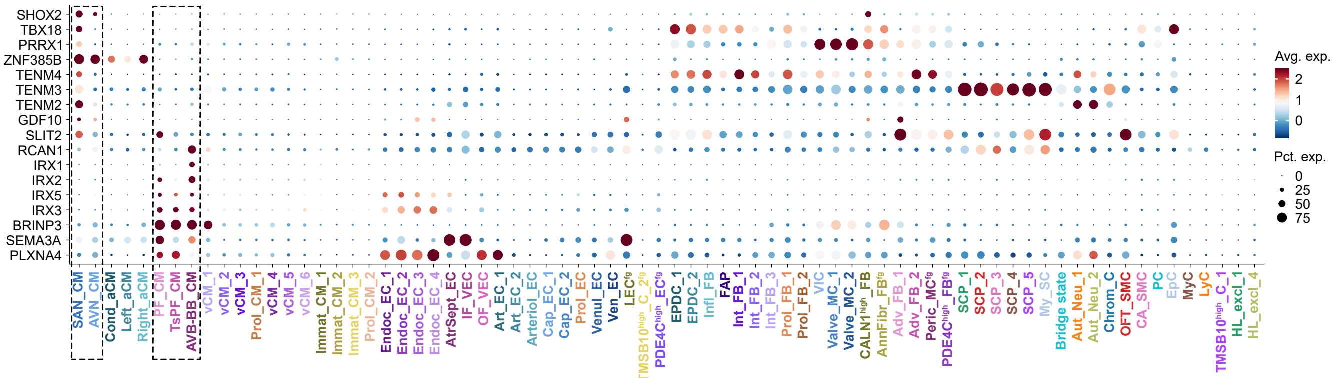

Supplementary Figure 9

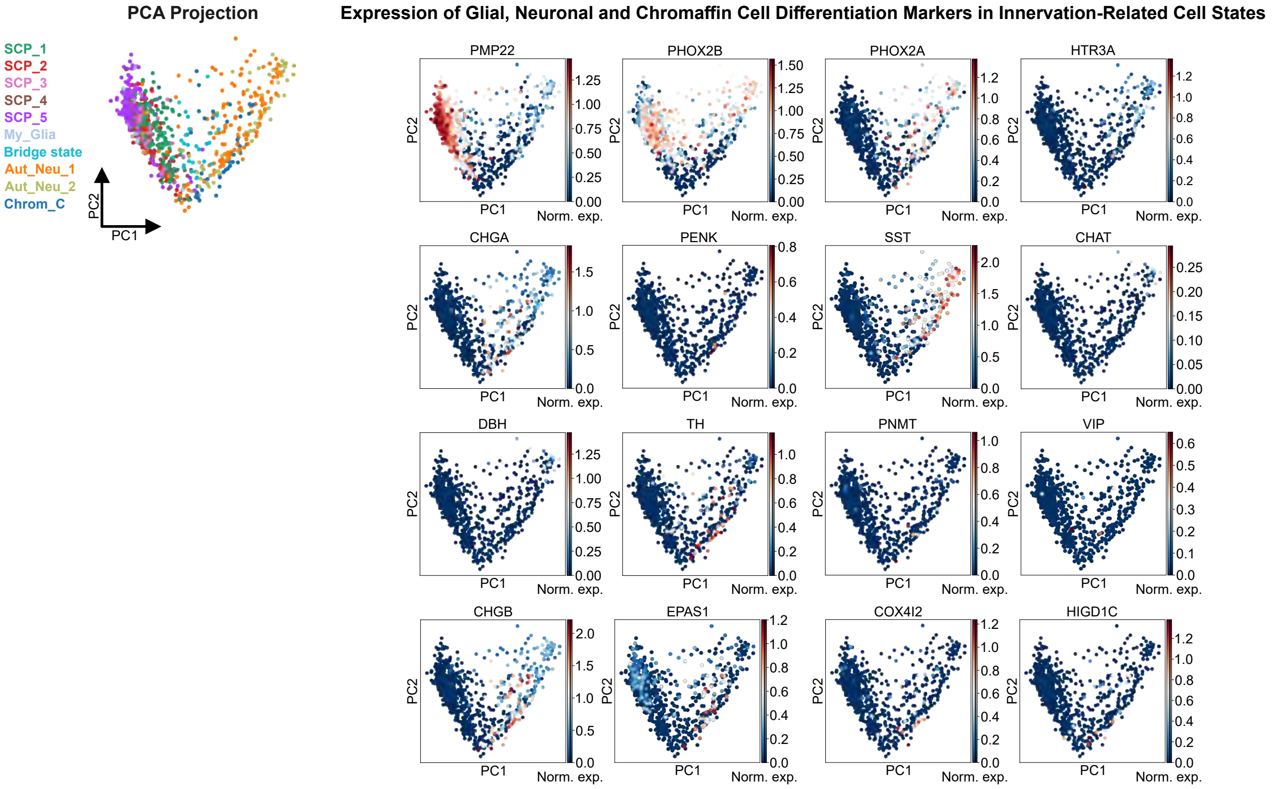

Supplementary Figure 10

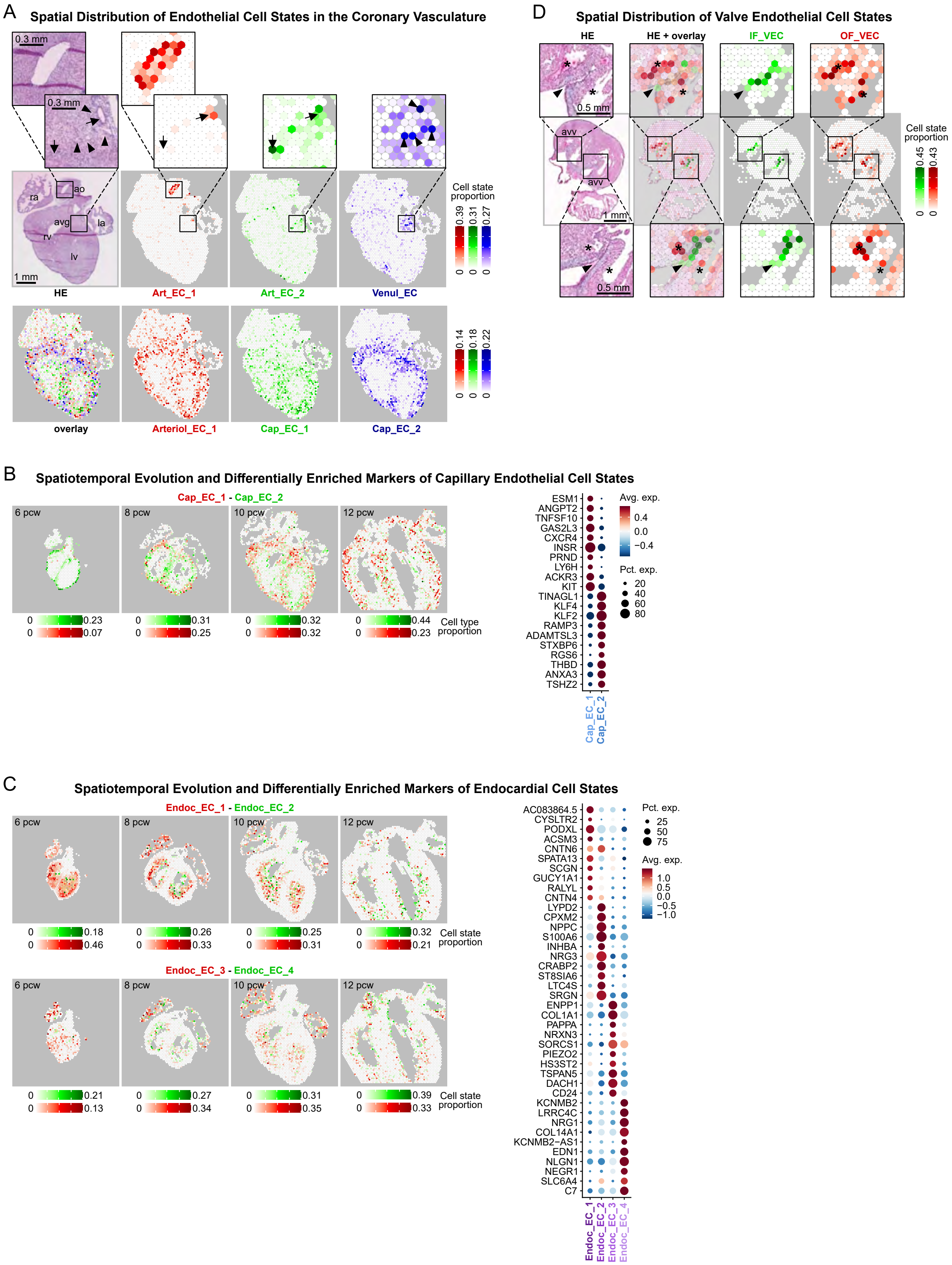

Supplementary Figure 11

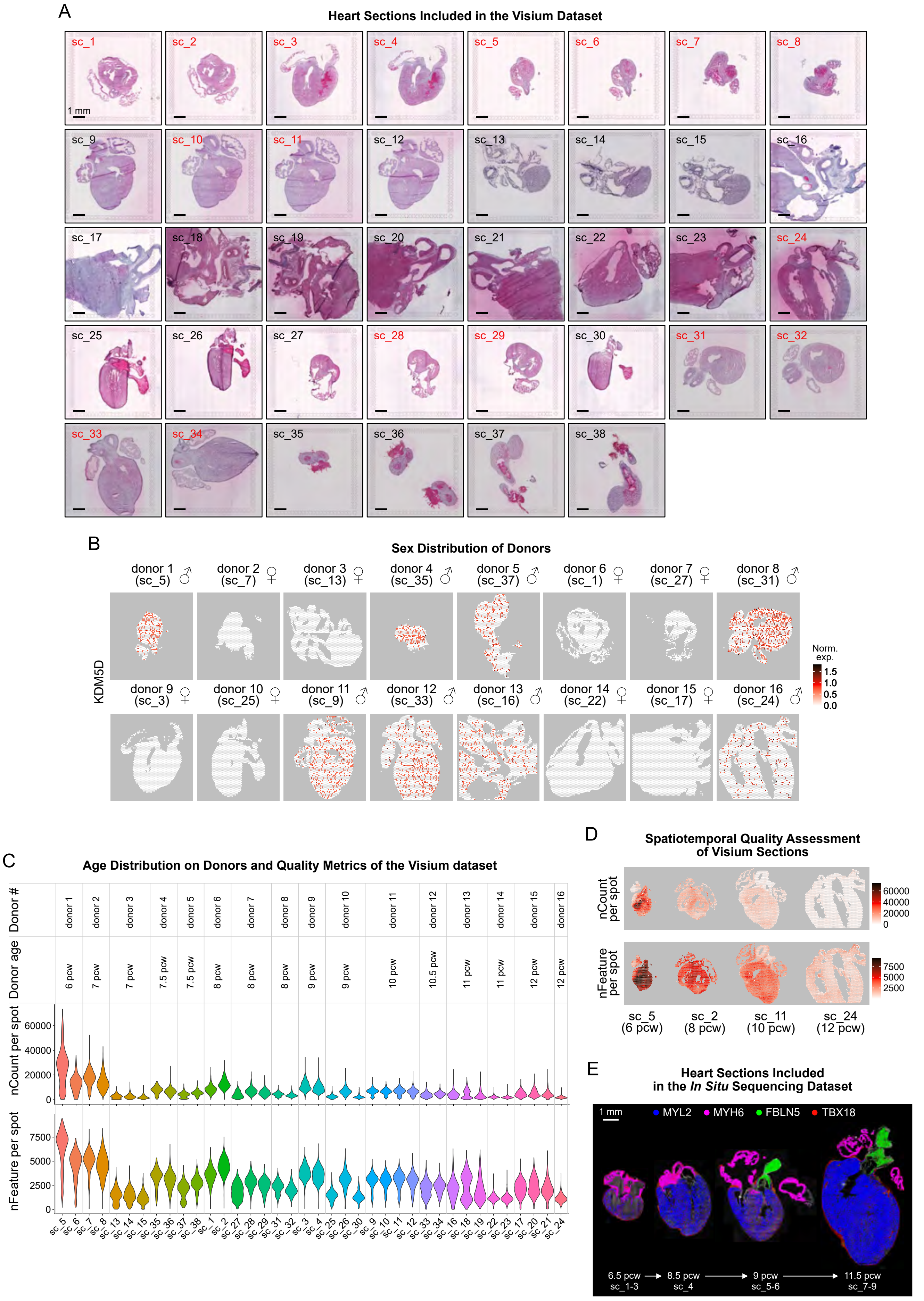

Supplementary Figure 12

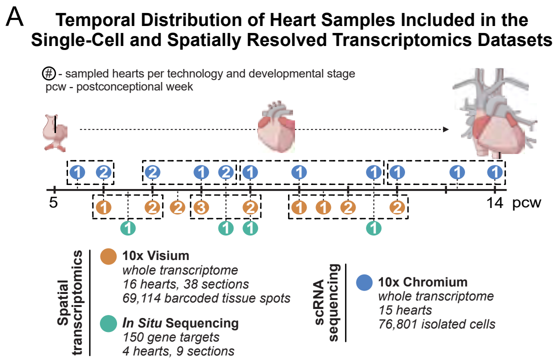

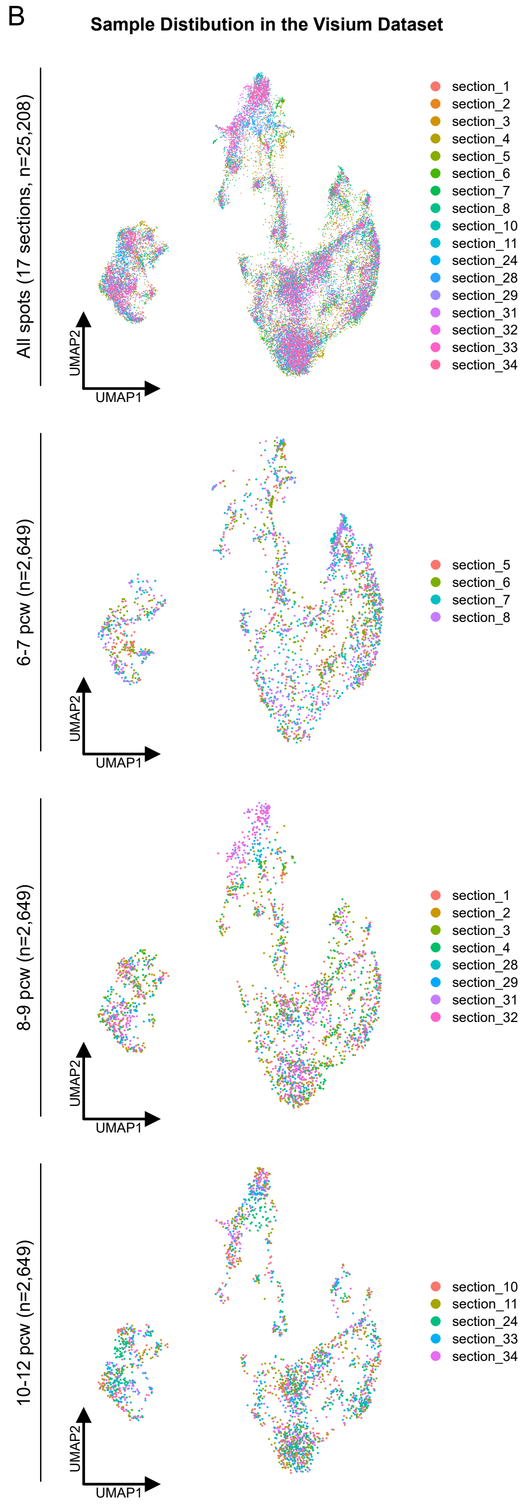

Supplementary Figure 13

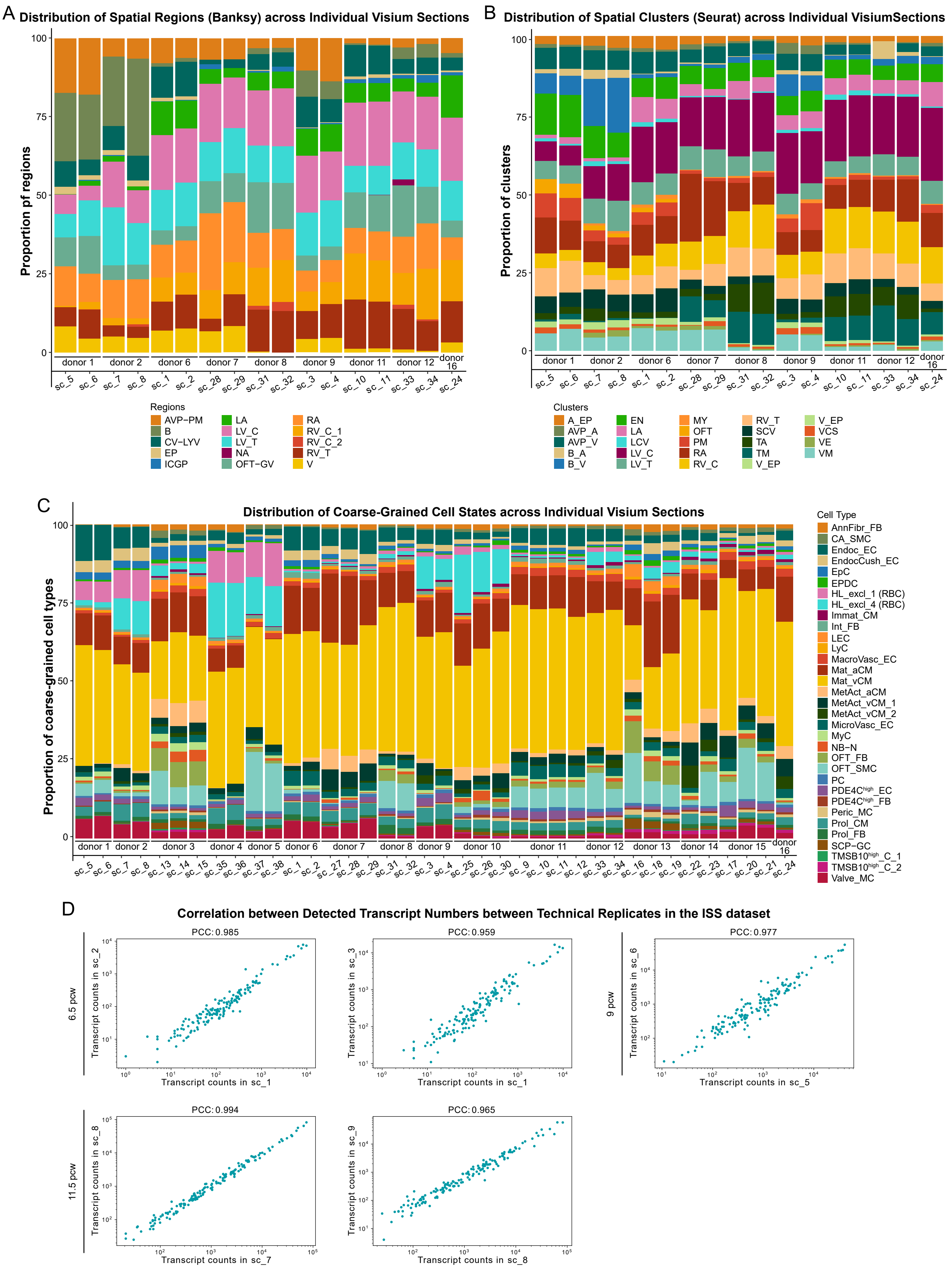

Supplementary Figure 14

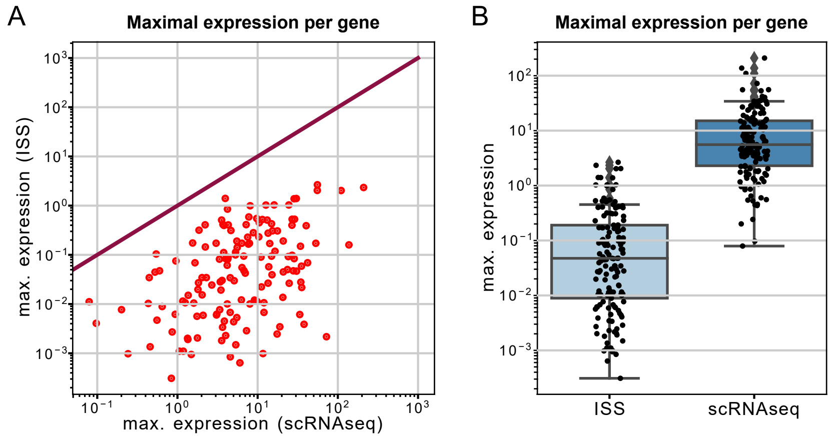

Supplementary Figure 15

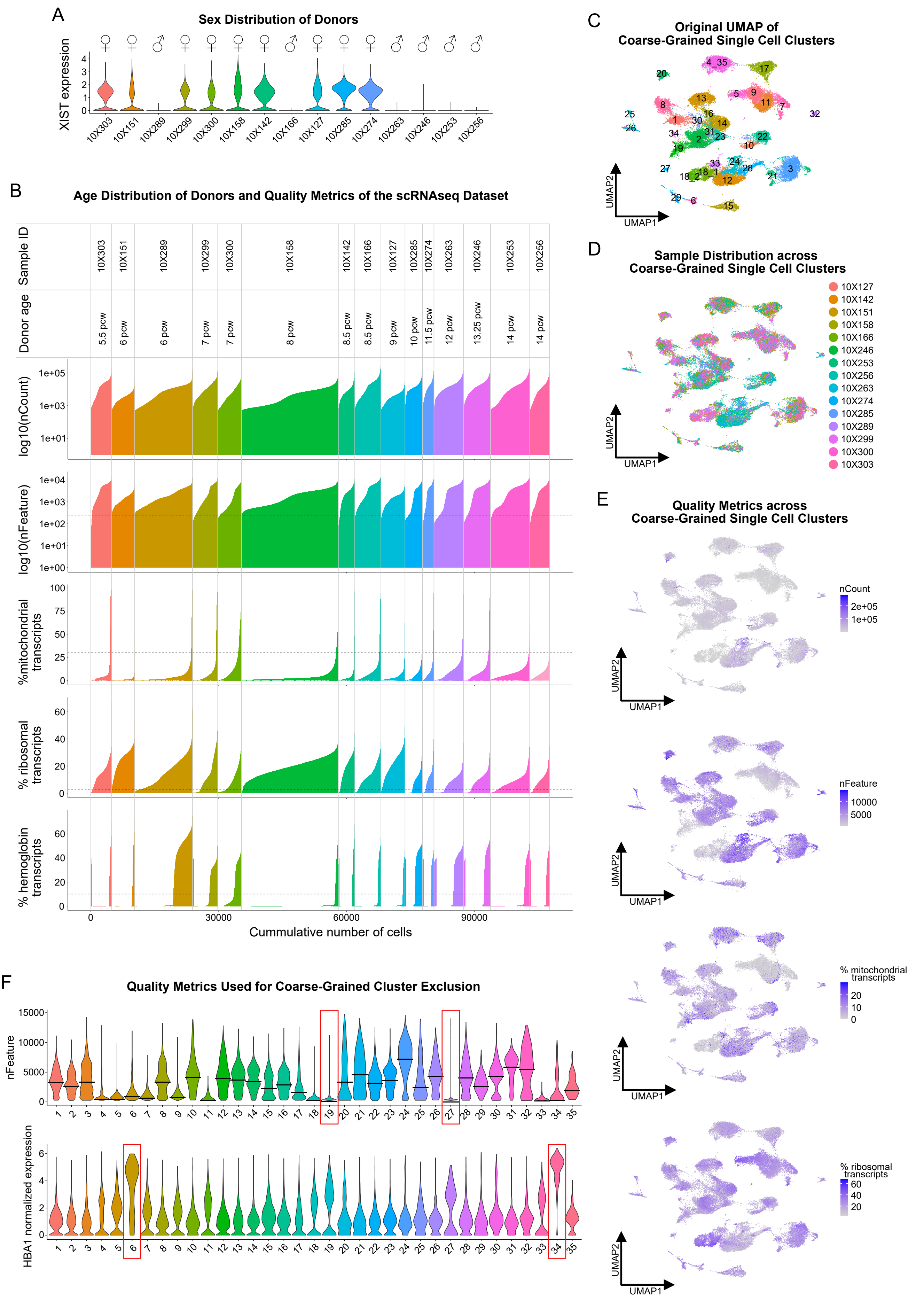

Supplementary Figure 16

Sample Distibution in the Chromium Dataset

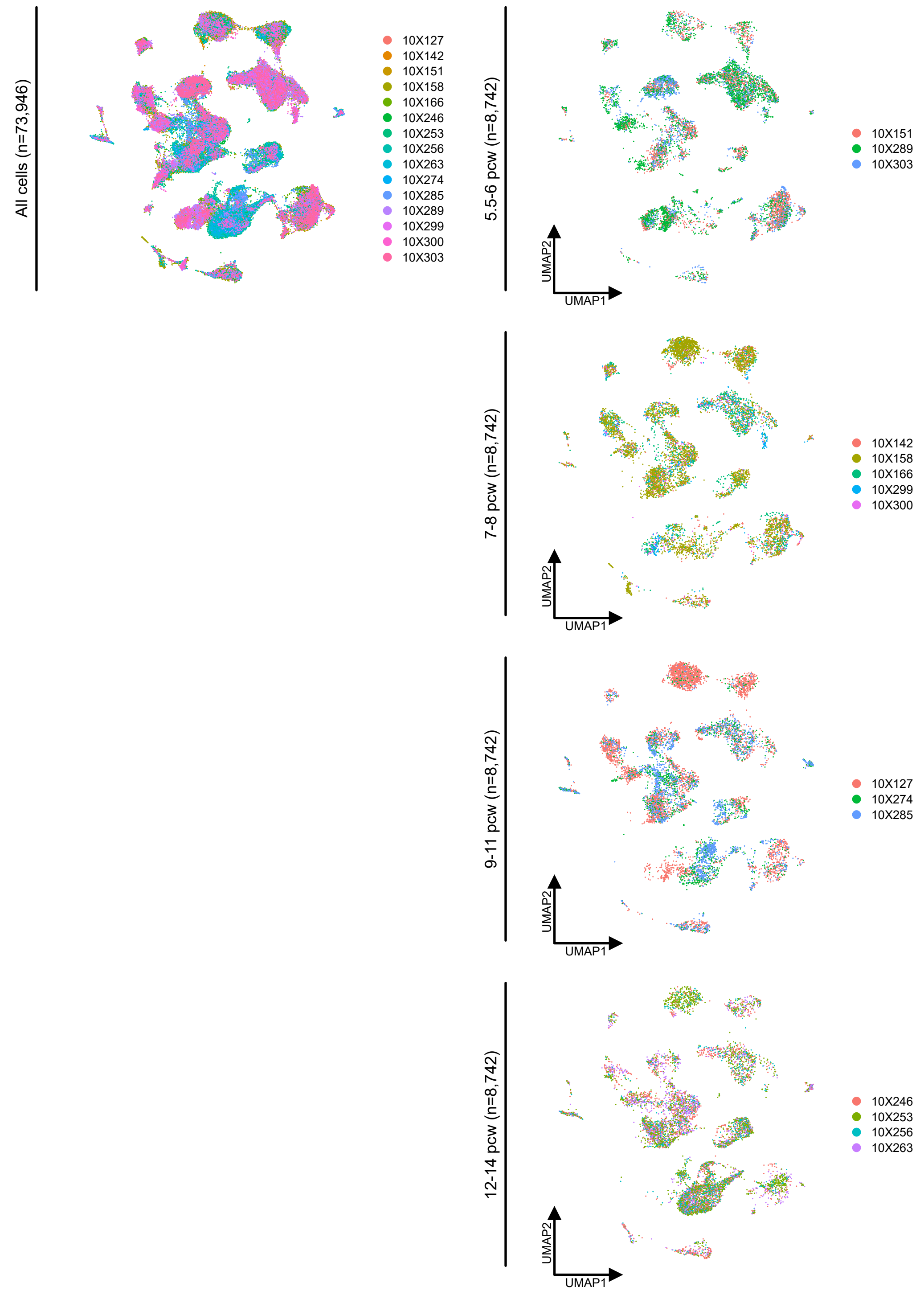

Supplementary Figure 17

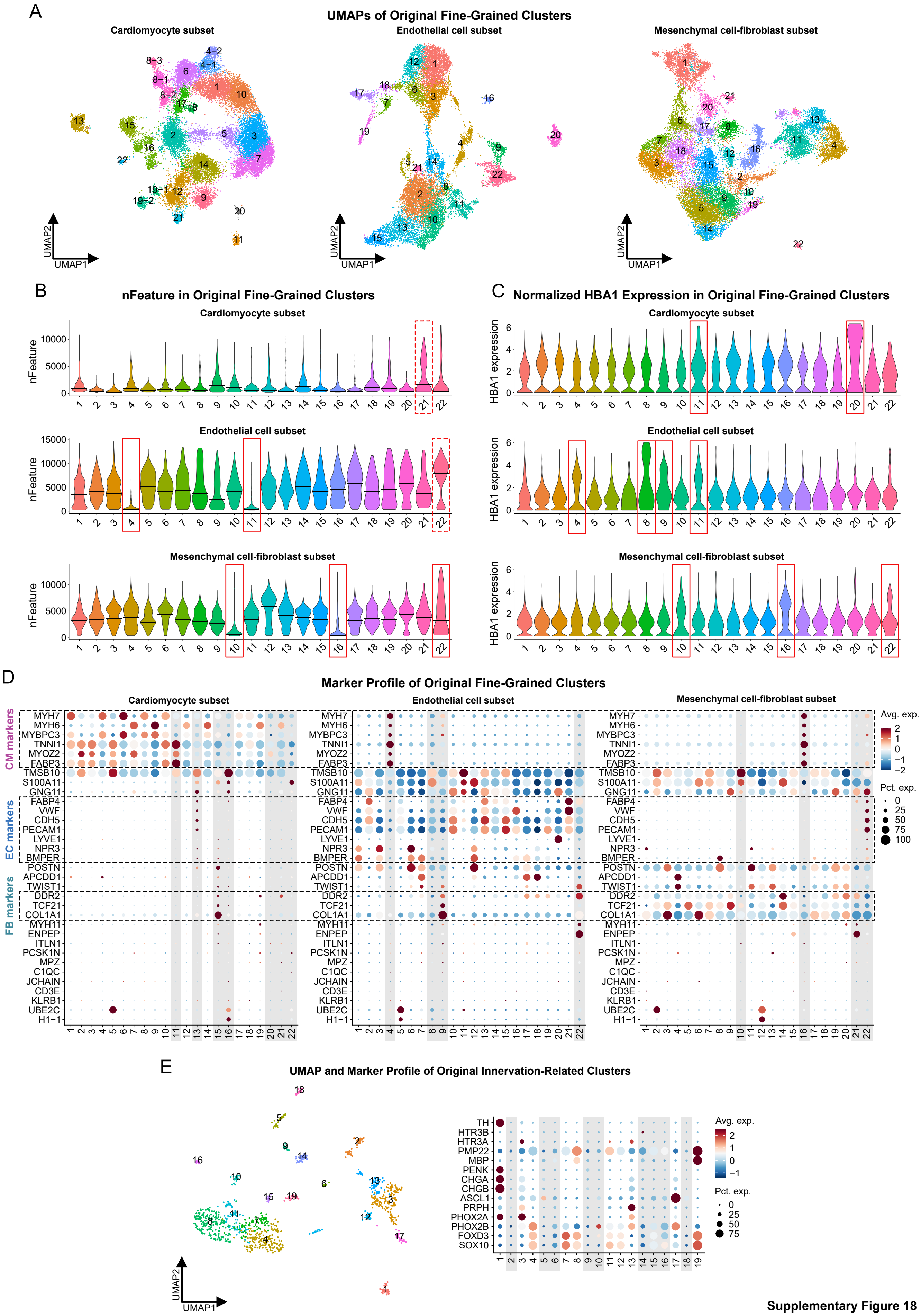

Supplementary Figure 18

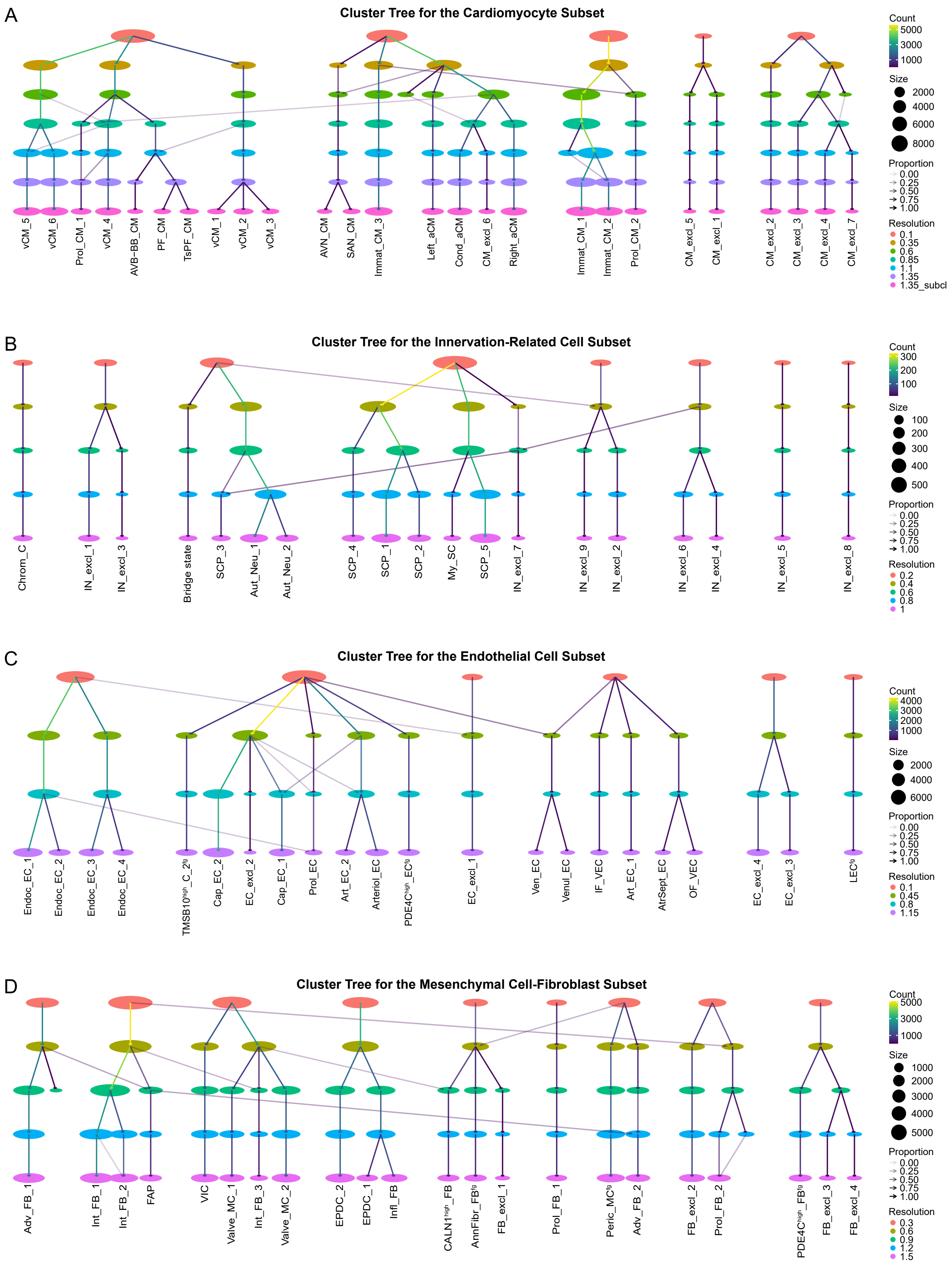

Supplementary Figure 19

SUPPLEMENTARY METHODS

Processing and Analysis of Visium Spatial Gene Expression Data

Sequenced libraries were processed using Space Ranger (v.1.2.1; 10x Genomics). Reads were aligned to the built-in human reference genome (GRCh38 v.2020-A, Ensembl 98). Further processing and data analysis of the spatial data was performed in R Statistical Software (v.4.0.5)[1] using STUtility (v.0.1.0)[2] and Seurat (v.4.1.1)[3] packages. The created count matrix was filtered for MALAT1, ribosomal, mitochondrial, and hemoglobin genes. Spots with fewer than 200 genes were removed from downstream analysis. The processed data was described with a median of nFeature_Spatial and nCount_Spatial of 2,191.5 and 4,179, respectively. Each tissue section was normalized separately using the SCTransform function of the Seurat package. Sections with at least 3 heart chambers present were selected for clustering. In the following steps, principal component analysis (PCA) and sample integration using Harmony (v.1.0)[4] was performed. Unsupervised clustering of spots was performed using the shared nearest neighbor (SNN) algorithm from the Seurat package. Uniform manifold approximation and projection (UMAP) was used for cluster visualization. Temporal evolution of spatial clusters was analyzed by embedding the original UMAP with three age groups (age 1: 6-7 pcw; age 2: 8-9 pcw; age 3: 10-12 pcw). Downsampling in age groups 2 and 3 was performed to equalize the spot numbers across embeddings (n = 2,649 per group). FindAllMarkers() function from Seurat package (logfc.threshold = 0.5, only.pos = TRUE, min.pct. = 0.01) was applied to identify differentially expressed genes (DEGs) between clusters. This method uses the Wilcoxon Rank Sum Test (two-sided) and is corrected for multiple testing with Bonferroni correction using all the genes in the dataset. Subsequently, all clusters were manually annotated. Spatial feature plots were generated with the STUtility package.

In addition to standard clustering, region segmentation was performed on the same subset of sections using the R tool Banksy (v.1.0.0)[5]. This spatially aware clustering tool enables deciphering neighborhoods using both the transcription information of a spot and its neighbors. The hyperparameter k was set to 6 to select the first layer of immediate neighboring Visium spots. The second hyperparameter, lambda, was set to 0.8 to focus the algorithm on region segmentation, as it weighs the importance of the spatial component for clustering. Banksy neighborhood-augmented feature space's clustering was performed with the default Leiden algorithm at a resolution of 0.9. The resulting regions were transferred to the existing object for exploration and visualization.

Non-negative matrix factorisation (NMF) was performed jointly, using the singlet package (v.0.99.36)[6] in R implemented in the semla toolkit, on the entire Visium dataset. The parameter k was manually fixed to 20, resulting in 20 shared factors across the dataset. This method, widely used in the single-cell and spatial omics field, decomposes data variability into gene modules that covariate in a latent space. Each factor consists of all the initial variable features ranked by order of influence in the factor-forming feature loadings.

Hematoxylin-eosin images of the analyzed tissue sections were included in the figures after linear adjustments of intensity and contrast, performed in Affinity Designer. In composite figures with micrographs of more than one tissue section, orientation of the images was adjusted according to the anatomical position (left-right, superior-inferior) of the sampled cardiac structural components.

Quality Assessment of the Visium and *In Situ* Sequencing Datasets

The original microscopic images, quality metrics, sex and age distribution of the heart sections included in the Visium dataset are displayed in Suppl. Fig. 12A-D, while representative sections included in the ISS dataset are visualized in Suppl. Fig. 12E. Batch effects in the data were visually assessed in the section selection used for clustering analysis, by embedding the UMAP with the individual samples, either in an integrated or in an age-resolved manner (3 age groups) (Suppl. Fig. 13A-B). Furthermore, cluster and region distributions were computed per section and presented as bar charts, enabling an evaluation of consistency between technical replicates (Suppl. Fig. 14A-B). Similarly, coarse-grained cell type proportions were computed per section for the entire dataset and visualized in bar charts (Suppl. Fig. 14C).

Consistency between technical replicates in the ISS dataset was evaluated based on agreement between detected transcript numbers of individual targets, by calculating Pearson correlation coefficient between paired sections (Suppl. Fig. 14D). Capture efficiency of ISS was assessed relative to that of scRNAseq, by calculating maximum gene expression values in coarse-grained cell types shared between the two datasets (Suppl. Fig. 15A) and comparing the mean expression for each method, revealing that scRNAseq was 62.5 times more efficient than ISS across the 149 common genes assessed (Suppl. Fig. 15B).

Processing and Filtering of the Single-Cell RNA Sequencing Dataset

Further processing and analysis of the scRNAseq data was performed in R Statistical Software (v.4.3.1)[7], using the Seurat toolkit (v.4.3.0.1)[3]. The data was jointly handled as one Seurat object, and

an initial filtering step was applied, removing cells with more than 30% mitochondrial transcript counts. Cells with less than 250 unique genes or less than 3% ribosomal protein-coding transcript content were also removed unless they also had more than 10% hemoglobin gene expression. The latter consideration was included to keep red blood cells for the subsequent doublet detection step. From the 107,673 initial cells imported, 101,104 cells remained after the first filtering round.

Doublet detection was performed separately for each sequencing dataset using the DoubletFinder R package (v.2.0.3)[8], with expected multiple rates defined according to 10x Genomics guidelines. The data was then merged again, and a second filtering step was applied, removing expected doublets (4,568 doublets), as well as cells with more than 10% hemoglobin gene expression (19,742 red blood cells), identifying 76,991 high-quality cells. Data was then scaled using the Seurat function ScaleData() based on the top 4,000 most highly variable genes, regressing out the number of reads, number of unique genes, percentage of ribosomal genes, percentage of mitochondrial genes, percentage of hemoglobin genes, percentage of heat shock protein-related genes, as well as S and G2M scores. Dataset integration was performed using the R package Harmony (v.0.1.1)[4], leveraging the top 50 principal components. The processed data was described with a median of nFeature_RNA and nCount_RNA of 2,417 and 4,838, respectively.

Quality metrics, sex and age distribution of the heart samples included in the scRNAseq dataset are displayed in Suppl. Fig. 16A-B.

Age Categorization for Single-Cell Data Analysis

To facilitate a temporal analysis of the scRNAseq dataset, samples were classified into four age groups: age 1 (5.5-6 pcw), age 2 (7-8 pcw), age 3 (9-11 pcw), and age 4 (12-14 pcw). This categorization enabled a comprehensive examination of temporal trends within the data, providing insights into developmental patterns across different developmental stages.

Coarse-Grained Clustering and Analysis of the Single-Cell RNA Sequencing Data

Coarse-grained clusters (referred to as HL, as in ‘high level’, in the code) were obtained using the Louvain community detection algorithm on an SNN graph built from the harmony embedding (nearest neighbors set to 20) with the resolution parameter set to 1.8. The UMAP embedding was computed from the harmony embedding (top 50 components), with 10 neighbors and 100 epochs. To identify differentially expressed genes and facilitate the computation, the clusters were downsampled to have a maximum of 150 cells, and pair-wise comparison of each cluster against all other clusters was performed using the FindAllMarkers() function from Seurat (max.cells.per.ident = 300,

logfc.threshold = 0.1, min.pct. = 0.05). This method uses the Wilcoxon Rank Sum Test (two-sided) and is corrected for multiple testing with Bonferroni correction using all the genes in the dataset. To maintain the highest heterogeneity within each cluster, the x=150 cells per group were randomly picked with the R function sample(colnames(data)[data$clusters_louvain_oi == x], size = sample_size[x]), without using any QC condition. Further subclustering of the mixed coarse-grained cluster 18 was performed with the same settings and principal components, except for using the 10 nearest neighbors and a resolution of 0.25, resulting in two distinct subclusters. The small coarse-grained cluster 35 was merged with the highly similar cluster 4 for further analysis.

For temporal analysis of the coarse-grained cluster distribution, UMAP embedding was performed across the four predetermined age groups. Downsampling in age groups 1, 2 and 4 was performed to equalize the cell numbers across embeddings (n = 8,742 cells per group).

The original coarse-grained clusters and related quality metrics are displayed in Suppl. Fig. 16C-E. Coarse grained cluster 6 and 34 marking red blood cells, and 19 and 27 featuring low quality cells were excluded from downstream analysis (Suppl. Fig. 16F), leaving 73,946 cells included in comparative analysis on the coarse-grained level. Batch effects in our data were visually assessed by embedding the coarse-grained UMAP with individual samples, either in an integrated or in an age-resolved manner (4 age groups) (Suppl. Fig. 17).

Fine-Grained Clustering and Analysis of the Single-Cell RNA Sequencing Data

To create fine-grained clusters (occasionally referred to as DL, as in ‘deep level’, in the code), cardiomyocyte (referred to as CM in the code), endothelial (referred to as EN in the code), mesenchymal cell-fibroblast (referred to as FB in the code), and innervation-related cell (referred to as IN in the code) subsets were all processed similarly. The subsetted data was again scaled, this time based on the top 3,000 highly variable genes, and the same variables were regressed out as for the complete dataset. Again, the data was integrated using Harmony, based on the top 50 principal components for the specific subset. Clustering was based on the 30 nearest neighbors and calculated using Louvain community detection with a resolution of 1.35 for cardiomyocytes, 1.25 for endothelial cells, 1.5 for fibroblasts, and 1 for innervation-related cells. UMAPs were calculated based on the top 50 integrated principal components and 30 neighbors, using 100 epochs. Marker genes were identified using FindAllMarkers() from Seurat, using the same settings as for the coarse-grained clusters. The pacemaker-conduction system-related cardiomyocyte clusters 4, 8, and 19 were further subclustered to increase granularity. For this purpose, Louvain community detection was applied to each of these clusters, calculated from the top 30 nearest neighbors with a resolution of 0.5.

Fine-grained cardiomyocyte clusters 11, 13, 15, 16, 20, 21, and 22, fine-grained endothelial clusters 4, 8, 9, and 22, fine-grained mesenchymal cell-fibroblast clusters 10, 16, 21 and 22, and fine-grained innervation-related cluster 2, 5, 6, 9, 10, 14, 15, 16 and 18 were excluded from downstream analysis, on the basis of low quality metrics, potential doublet contamination, or lack of consensus marker expression of the relevant cell types. The original UMAPs of these cell subsets, consensus marker expression profiles of the original fine-grained clusters, and parameters used as basis for cluster exclusion are presented in Supplementary Figure 18A-E.

Additionally, we used the clustree tool (v.0.5.1)[9] to visualize single-cell clusters as hierarchical trees, generated by increasing resolution at the subset level, to illustrate the varying degrees of transcriptional similarity among the fine-grained cell states identified in our analysis (Suppl. Fig. 19A-D). We manually selected different resolutions for each of the four fine-grained clustering analyses, ensuring that the incremental steps were consistent for each clustering and aligned with the final resolutions chosen for our analysis. This approach was necessary to maintain a regular progression in the clustering hierarchy while using the clustree tool with default parameters.

Spatial Mapping Using Cell State Deconvolution

The spatial mapping of the scRNAseq data onto tissue sections included in the Visium dataset was achieved through probabilistic inference using stereoscope (v.0.3.1)[10], effectively addressing spatial heterogeneity. By assuming a negative binomial distribution, this model estimates the proportions of single-cell states in every spatial spot. This guided decomposition process was executed separately for coarse- and fine-grained annotations. In the coarse-grained iteration, with 31 clusters remaining after excluding poor-quality clusters and red blood cells, the dataset was downsampled to the 500 cells containing the highest number of features in each population. Then, the top 5,000 most variable genes from this subset were utilized. Similarly, low-quality clusters were excluded in the fine-grained iteration, and the dataset was downsampled to the top 250 cells with the highest number of features for each of the 72 remaining cell states. The top 5,000 most variable genes from this subset were employed for the analysis. The stereoscope analysis ran with a batch size of 2,048 and 50,000 epochs for parameter estimation and proportion inference steps in both the coarse- and fine-grained iterations. A complementary, strictly age-matched cell type mapping was performed using the Visium and coarse-grained single-cell data of the same age window. After excluding single-cell data from hearts not matching the age range of the Visium sections, deconvolution with stereoscope was performed with the 3 previously determined age groups (age 1: 6-7.5 pcw; age 2: 8-9 pcw; age 3: 10-12 pcw) with 500 cells per population, the top 5,000 most variable genes, and unchanged stereoscope parameters.

Spatial cell state prediction maps were generated using MapFeatures() from the semla package (v.1.1.6)[11]. A max_cutoff parameter of 0.99 was applied to mitigate potential biases from outliers in coarse-grained clusters, while no max_cutoff was used for fine-grained clusters. In the figures, these maps were visualized alongside the corresponding intensity- and contrast-adjusted hematoxylin-eosin micrograph of the relevant tissue section.

Annotation of Spatial and Single-Cell Clusters

Annotation of the spatial clusters was performed manually, based on the relative enrichment of consensus markers of cell states with characteristic spatial distributions, and the relation between known anatomical landmarks and the observed localization of spatial clusters within the analyzed heart sections. For the coarse- and fine-grained single-cell clusters, a spatially aware annotation strategy was utilized. Cell state identification was based on the hierarchy of three features: first, the relative enrichment of consensus cell type/state markers was considered, discerning major cell populations and minor cell states with known transcriptomic profiles. For further refinement, the deconvolution results of the integrated single-cell and spatial transcriptomics datasets were utilized, factoring in the predicted localization of the analyzed cell states. Finally, enrichment of gene signatures consistent with different biological states in relation to maturation, metabolism, or proliferation were considered, in case the previous two features alone did not sufficiently explain the distinct characteristics of the cluster. Where possible, cluster names were selected to match the conventional cell state nomenclature, while in other cases they were formulated to reflect the distinguishing spatial or transcriptomic feature of the population in question. Fine-grained cell states with annotations identical to coarse-grained clusters are distinguished by an ‘fg’ (as in ‘fine-grained’) superscript.

Dominant marker genes of single-cell clusters were identified by sequential filtering of the top 40 DEGs by lowest p-values (p_val), top 20 DEGs with the highest percentage difference (pct.1-pct.2), and top 5 or 10 DEGs with the highest average log2 fold change (avg. log2FC), which were then visualized by dot plots across the compared clusters.

Assessment of Ion Channel Profiles of Cardiac Pacemaker-Conduction System-Related Cell States

After differential gene expression analysis across all fine-grained cardiomyocyte clusters, a comprehensive list of ion channel-encoding DEGs ($p < 0.05$, avg. log2FC > 0), enriched in any of the fine-grained cardiomyocyte clusters, was extracted. Next, this list was filtered in parallel for genes expressed in more than 10% of cells (pct. exp. > 0.1) of the SAN_CM or AVN_CM clusters, or of the

AVB-BB_CM, PF_CM or TsPF_CM clusters, respectively. In Fig. 3F, genes with higher average expression observed in SAN_CMs or AVN_CMs compared to the contractile atrial clusters Right_aCM, Left_aCM and Cond_aCM were included. In Ext. Fig. 3F, genes with higher average expression observed in AVB-BB_CMs, PF_CMs or TsPF_CMs compared to the contractile ventricular cluster vCM_4 were included. The compiled gene lists were then assessed for biological relevance in the specification of the pacemaker-conductive phenotype.

Analysis of Cell State Co-Detection and Identification of Spatial Compartments and Cellular Niches

Spatial compartments and cellular niches were identified based on co-detection scores, obtained by calculating the commonly used Pearson correlation coefficients between the distribution of each fine-grained cell state pair, based on the spatial cell proportion predictions of the stereoscope analysis. The matrix of these in-pair calculated correlation scores, referred to as co-detection scores, served as a proxy for appreciating the level of spatial overlap between the fine-grained cell states despite the sparsity of the data, outlining groups of cell states with close spatial association. Co-detection scores in our dataset ranged between -0.25 and 0.55 (95th percentile = 0.10) in the age-resolved, and between -0.2 and 0.42 (95th percentile = 0.09) in the merged dataset. The co-detection matrices were further visualized on heatmaps using geom_tile() from ggplot2 (v.3.4.4)[12]. Spatially co-localised cell types were described with positive co-detection scores, while non-co-localised cell types were described with negative ones. Since 72 cell types were used for the deconvolution, inherently many proportions were close to zero, hence, the co-detection heatmap resulted in many scores around zero. Those indicate neither co-detection, nor non-co-detection. To capture niches clearly, a co-detection graph network was built, where co-detection scores above a manually selected arbitrary threshold of 0.07 were kept. All the other scores were set to 0 since we considered building graphs with negative values not meaningful. From the remaining co-detection matrix, we used graph_from_adjacency_matrix() function from the igraph (v.1.5.1)[13] package in R (weighted=TRUE, mode="undirected", diag=FALSE) which represents high co-detection scores by shorter edges between nodes (nodes appear closer in the graph) and degree() function from the igraph package in R to calculate the degree for each node influencing the size of the nodes (the more connections, the bigger the node). The graph was plotted using the plot() function from igraph package (vertex.size=deg*1.1) and other parameters left to default. In our case (less than 1000 vertices, and no other attributes), the network layout has been selected by default by the tool, resulting in the Fruchterman-Reingold algorithm. The resulting network (Fig. 7A, lower panel) is useful for readability, where niches and compartments were manually annotated based on the cell states' spatial relation to known cardiac structural components.

These steps were performed on the merged Visium dataset, as well as in an age-resolved manner (age 1: 6-7.5 pcw; age 2: 8-9 pcw; age 3: 10-12 pcw) to describe spatial cell relations in a temporal context.

Spatial association of manually selected niches was visualized with a new in-house function CellCol.R, an adaptation of the MapFeatures() function built upon the package semla (v.1.1.6)[11]. This function enables simultaneous visualization of per-spot predicted cell type proportion of two or three cell states, overlaid onto the section of interest in the Visium dataset. Overlap of co-detected cell states was presented through a scaled color blending using blend_colors() function from colorjam (v.0.0.26.900)[14].

Cell-Cell Communication Analysis

Single-cell clusters in spatial proximity were selected for cell-cell communication (CCC) analysis. Intercellular communication between ligands (L) and receptors (R) was inferred using CellPhoneDB (v.2)[15] implementation through the tool provider Liana (v.0.1.13)[16] in R (v.4.3.1). The mean of the gene expression for known ligand and receptor pairs (LR) from the curated CellPhoneDB was calculated by randomly permuting (100 epochs) cell labels in the subset, generating a null distribution for each LR event between each cluster in the subset. The p-value of 0.05 was used as threshold for selecting significantly enriched LR pairs between cell states having a mean expression of LR equal to or higher than the mean of that specific LR event calculated with the null distribution. The default mode *magnitude* within the function rank_method() was used to order LR events based on expression level. A selected subset of the identified ligand-receptor interactions was visualized as dot plots across the analyzed cell state pairs.

Additionally, a subset of co-localised cell types were selected from the niche network (SAN_CM and its direct neighbors and Chrom_C) to assess potential LR interaction described in a recently published, custom neural-GPCR module of CellPhoneDB (41586_2023_6311_MOESM5_ESM)[17]. After selecting the pairs of protein_name_a (ligands) and protein_name_b (receptors) present in the module, separate lists of ligands and receptors were generated for every cluster of interest from their differentially expressed genes. The cell-cell interactions were evaluated bi-directionally, SAN_CM (donor) to its neighbors (receivers) and from neighbors (donors) to SAN_CM (receiver), using the gene lists accordingly. The interactions were scored for each present LR pair by multiplying the average expression of the ligand and the receptor in their respective cell types with the AverageExpression(group.by = c("annot_dl")) function from the Seurat package. The results are presented as heatmaps where LR pairs are colored according to their

score, while gray squares mark LR pairs where the ligand or receptor was not enriched in the assessed cell states within their respective cellular subsets.

Spatial LR interactions were visually presented in tissue sections for a selection of known LR pairs by calculating their geometric means per spot. The spatial LR interaction scores were plotted using an in-house adaptation of the MapFeatures() function from the semla package.

RNA Velocity and Pseudotime Analysis

RNA velocity analysis for innervation-related fine-grained clusters was performed on the top 5,000 variable features calculated with filter_genes_dispersion() from scVelo (v.0.3.0)[18] in Python (v.3.8.17). PCA was performed with Scanpy (v.1.9.5)[19], followed by recomputing the neighboring graph (scanpy.pp.neighbors(), n_neighbors = 25). Velocity was plotted on the PCA embeddings after running scv.tl.velocity() and scv.tl.velocity_graph(). Pseudotime was calculated separately on the top 5,000 variable features (highly_variable_genes()) with scFates (v.1.0.6)[20] in Python (v.3.11.6). PCA and clustering were performed similarly as for the RNA velocity analysis, resulting in the same embedding. Curves of the PCA were calculated using the ElPiGraph algorithm implemented in scFates (scFates.tl.curve()). Subsequently, pseudotime was calculated with scFates.tl.pseudotime() (n_jobs = 20, n_map=100), and both pseudotime and expression of markers were visualized on the PCA embedding (scanpy.pl.pca()).

Transcription Factor Inference Using Gene Regulatory Networks

Inference of transcription factor (TF) activity was performed using the Scenic pipeline (v.1.3.1)[21] in R (v.4.3.1). Gene regulatory networks (GRN) were generated by analyzing co-expression across the scRNAseq data with GENIE3 (v.1.22.0)[22], based on the overlap between the most variable features calculated during clustering of the dataset and the provided databases (hg19-500bp-upstream-10species.mc9nr.feather and hg19-tss-centered-10kb-7species.mc9nr.feather). Co-expression of the remaining TFs in the network were converted into regulons using runSCENIC_1_coexNetwork2modules(). The implemented AUCell from Scenic was used to score every regulon in each individual cell. Then, known TF-binding motifs were used to prune interactions from the GRN (regulatory modules or regulons). Relevant regulons were retrieved if their specific TF was differentially expressed (FindMarkers(), test.use = “wilcox”, avg_log2FC > 0 and p_val < 5e-7) in the cluster of interest, compared to the other clusters of the specific group. Regulated genes in the dataset were similarly selected (FindMarkers(), test.use = “wilcox”, top 1,000 based on p-value). The

resulting network of regulatory TFs and their associated target genes was constructed as a network graph using the igraph package[13].

The entire list of inferred transcription factors and target gene pairs, as well as differentially expressed transcription factors in the investigated cell states are presented in Suppl. Table 9-16.

Regulon enrichment analysis in selected endothelial cell states was performed for each cell using AddModuleScore() from Seurat, scoring the average expression of the target genes of a TF compared to control sets of genes. The enrichments were visualized on violin plots for the three selected clusters in an age-resolved manner, where age groups 3 and 4 were merged (creating an age group representing 9-14 pcw) in order to decrease the difference in cell numbers between the compared populations.

Pathological Gene Sets Enrichment Analysis

Known sets of gene markers associated with cardiovascular pathologies with high level of evidence (green marking) were retrieved from the PanelApp from Genomics England database (https://panelapp.genomicsengland.co.uk/) for gene set enrichment analysis (GSEA) (panels: Arrhythmogenic right ventricular cardiomyopathy (Version 3.11), Brugada syndrome and cardiac sodium channel disease (Version 3.10), Cardiac arrhythmias (Version 13.37), Catecholaminergic polymorphic VT (Version 4.6), Dilated and arrhythmogenic cardiomyopathy (Version 2.31), Dilated cardiomyopathy and conduction defects (Version 1.94), Familial non syndromic congenital heart disease (Version 1.86), Hypertrophic cardiomyopathy (Version 4.13), Idiopathic ventricular fibrillation (Version 1.2), Left ventricular noncompaction cardiomyopathy (Version 1.4), Long QT syndrome (Version 3.8), Paediatric or syndromic cardiomyopathy (Version 4.10), Primary lymphoedema (Version 3.11), Progressive cardiac conduction disease (Version 2.8), RASopathies (Version 1.81), Sudden death in young people (Version 1.15), Thoracic aortic aneurysm or dissection (GMS) (Version 3.16)). Each set of genes was used to compute its respective enrichment in each cell of the dataset using AddModuleScore() from Seurat, scoring the average expression of the gene set compared to control sets of genes. The mean of the enrichment scores was computed per cluster for the coarse- and fine-grained annotation layers. The resulting mean values were visualized on heatmaps representing the gene set of interest across every cluster using geom_tile() from the ggplot2 package.

Calculation of Single-Cell Cluster Proportions

The relative sizes of coarse-grained clusters, as well as the fine-grained cluster representing FAPs, were assessed based on the ratio of cells in the cluster of interest, compared to the total number of cells

in the relevant subset, and visualized with bar graphs in Microsoft Excel. The total number of cells in coarse- and fine-grained single-cell clusters, also per analyzed heart and sequencing sample, is displayed in Supplementary Table 17-18.

Abbreviations

A list of abbreviations used in the study, along with their expansions, is provided in Suppl. Table 19.

SUPPLEMENTARY DISCUSSION 1

Overview:

- Spatial clusters and regions in the developing human heart
- Transcriptomic heterogeneity of coarse-Grained cardiomyocyte, endothelial cell and mesenchymal cell-fibroblast clusters
- Temporal changes in cardiac cell composition
- Molecular analysis of mural cell states
- Molecular analysis of blood-related cell states
- Spatial heterogeneity of fine-grained mesenchymal cell-fibroblast cell states
- Transcriptomic heterogeneity in epicardium-related cell states
- Temporal gene expression changes in coarse-grained endothelial cell and mesenchymal cell-fibroblast clusters
- Assessment of spatiotemporal transcriptomic patterns in the cardiac valves, outflow tract and great arteries

Spatial Clusters and Regions in the Developing Human Heart

For independent, clustering-based spatial profiling of the heart, we selected 17 sections from the Visium dataset, which included tissue areas from at least three cardiac chambers and provided an even coverage of the investigated timeframe (6th-12th postconceptional weeks). By Seurat-based, unsupervised clustering of the related 25,208 Visium tissue spots, we identified 23 spatial clusters (Fig. 1A-B, Ext. Fig. 1A, Suppl. Table 1). Two of these clusters aligned with blood remnants in the atria and ventricles (B_A, B_V), and one with myeloid cell transcriptomic signature (MY) appeared scattered across the myocardium; however, the remaining 20 clusters mapped to distinct cardiac regions (Fig. 1C-D, Ext. Fig. 1B).

Cardiac spatial clusters were broadly consistent across developmental stages (Ext. Fig. 1C). Still, we observed gradual disappearance of an early outflow tract-related cluster (OFT) and expansion of clusters representing the tunica media (TM) and adventitia (TA) of the developing large arteries (Ext. Fig. 1C-D). The early OFT cluster's transcriptomic profile substantially overlapped both with the TM and the valve mesenchyme-related VM clusters, likely due to their spatial proximity at early developmental stages (Ext. Fig. 1A). Notably, the VM-enriched gene *PENK*, a neural crest-derived mesenchymal cell marker described in mouse cardiac valves[23], was not detected in the early OFT cluster, indicating a later contribution of this cell type to valve development (Fig. 1D). Additional

clusters represented the epicardial-subepicardial (A_EP, V_EP) and endocardial-subendocardial (EN) layers, small and large coronary vessels (SCV, LCV), and the developing valve endothelium (VE) (Fig. 1B-D, Ext. Fig. 1A-B, E).

Our clustering approach delineated major myocardial compartments in a side-specific manner, facilitating the exploration of their transcriptomic differences (Fig. 1E, Ext. Fig. 1A-B, E). Besides several common atrial markers, we observed selective enrichment of *PITX2*, *COL2A1*, and *ADAMTS8* in the left (LA), and *ANGPT1*, *BMP10,* and *DKK3* in the right atrial myocardial clusters (RA). The ventricle-associated spots were further divided into compact (LV_C, RV_C) and trabecular clusters (LV_T, RV_T), marked by the relative enrichment of *HEY2* and *MT3*, versus *MB* and *FHL2*, respectively. Furthermore, we detected side-specific enrichment *NREP* in the left and *CKM* in the right compact layers, and of *LGALS3* and *IRX3* in the left and *MASP1* and *PPP1R12B* in the right trabecular compartments. The transcription factor IRX3 is associated with ventricular conductive phenotype specification[24], while galectin 3 (encoded by *LGALS3*) is established as a marker for adverse cardiac remodeling, primarily affecting the left ventricle[25]. A recent study proposed *MASP1* as a candidate gene for ventricular conduction disorders[26], and the MYPT2 protein (encoded by *PPP1R12B*) is a known regulator of cardiomyocyte contraction force generation[27]. Side-specific enrichment of these genes might potentially contribute to the distinct electromechanical properties of the two ventricles.

Furthermore, we found a spatial cluster characterized by high expression of cardiomyocyte stress- and hypoxia response-related genes (*ACTA1*, *NPPB*, *ENO1*, *LDHA*, *MIF*, *FAM162A*)[28,29] positioned around the papillary muscles (PM), likely reflecting mechanical strain on these structures (Fig. 1F, Ext. Fig. 1A-B, E). Additionally, tissue spots with conduction system cell signatures localized to the ventricular subendocardium (VCS) and the atrial side of the atrioventricular plane (AVP_A), consistent with the position of bundle branches and atrioventricular nodal tissue, respectively (Fig. 1B-D, Ext. Fig. 1A-B, E). Notably, we also observed a ventricular cluster of ambiguous identity, localized close to the atrioventricular plane (AVP_V), sharing markers with both AVP_A (*CNN1*, *XPO4*) and PM-MB (*ACTA1*, *MYL2*) clusters (Fig. 1B-D, Ext. Fig. 1A-B, E).

Additionally, we expanded our investigation with a spatially aware, neighborhood-based region identification strategy, by implementing the recently published tool Banksy[5]. With this approach, we identified 14 biologically relevant spatial regions within the same 17 Visium sections and annotated them based on their marker profiles and spatial distributions (Suppl. Fig. 1A-C, E). Overall, we found a good agreement between Banksy-based regions and Seurat-based spatial clusters (Suppl. Fig. 1D-E), especially for ones representing larger myocardial compartments (atrial RA and

LA, ventricular compact RV_C, LV_C and trabecular RV_T, LV_T clusters) and the outflow tract and great vessels (OFT and TM clusters). On the other hand, clusters dominated by signatures of monolayers (endocardial and epicardial clusters EN, V_EP and A_EP) or finer structural components (VCS cluster for ventricular conduction system components, PM cluster for papillary muscles and myocardial bands, SCV cluster for smaller coronary vessel structures), as well as ones with a more dispersed pattern (myeloid cell-rich MY cluster) or low abundance in the region (blood-dominated B_A cluster in the atria) were redistributed between the Banksy regions in the closest positions (Suppl. Fig. 1D). The neighborhood-based region calling also led to some more pronounced rearrangements of a few spatial clusters, such as in the cardiac valve-related mesenchyme cluster (VM), where several spots related to the semilunar valves shifted from the Banksy region demarcating the valves to the one of the closely associated outflow tract and great vessels (Suppl. Fig. 1D). At the same time, spots of the tunica adventitia cluster (TA) got separated between this region, a newly identified domain corresponding to components of the intracardiac ganglionated plexi (ICGP), and a mixed region representing larger lymphatic and coronary vascular structures (Suppl. Fig. 1D). Overall, the spatially aware, neighborhood-based region identification provides largely consistent results with our Seurat-based spatial clustering, however, by default, it is not adept at distinguishing fine or spatially dispersed structural components with limited size in two dimensions. At the same time, Banksy identified a region characterized by innervation-related markers, previously not distinguished by our Seurat-based clustering, highlighting the benefits of complementary use of the two approaches.

Transcriptomic Heterogeneity of Coarse-Grained Cardiomyocyte, Endothelial Cell and Mesenchymal Cell-Fibroblast Clusters

Based on their expression of consensus cell type markers, we identified seven cardiomyocyte (*MYH7*, *MYH6*, *MYBPC3*, *TNNI1*, *MYOZ2*, *FABP3*), six endothelial cell (*FABP4*, *VWF*, *CDH5*, *PECAM1*, *LYVE1*, *NPR3*, *BMPER*), and eight mesenchymal cell-fibroblast (*DDR2*, *TCF21*, *COL1A1*) coarse-grained single-cell clusters in our dataset (Fig. 2A-C, Suppl. Fig. 2A, Suppl. Table 2).

The seven coarse-grained cardiomyocyte clusters displayed prominent differences in the expression of maturation, metabolic state and cell cycle markers, based on which we identified mature (Mat_vCM, Mat_aCM) and metabolically active atrial and ventricular clusters (MetAct_aCM, MetAct_vCM_1-2), besides proliferating cardiomyocytes (Prol_CM) (Fig. 2C, E, Ext. Fig. 2A, Suppl. Fig. 2A). Additionally, we observed a population with lower cardiomyocyte-specific gene expression and a dynamic decrease in proportion (15.18% to 1.63%) over the investigated time frame, outlining immature cardiomyocytes (Immat_CM) (Fig. 2E-F, Ext. Fig. 2A, Suppl. Fig. 2A). Transcriptome-

based identities of coarse-grained endothelial cell clusters were in agreement with their predicted localization in the tissue, outlining endocardial (Endoc_EC) and endocardial cushion-related cells (EndocCush_EC), macro- (MacroVasc_EC) and microvascular (MicroVasc_EC) endothelial cells of the coronary vasculature, and lymphatic endothelial cells (LEC) (Fig. 2C, E, Ext. Fig. 2B). Specific markers for mesenchymal cell- and fibroblast subtypes in the fetal heart are currently lacking (Fig. 2C), thus positional cues are especially valuable to decipher cell identities. Using a spatially informed annotation strategy, we identified distinct coarse-grained populations of fibroblasts around the outflow tract and developing great arteries (OFT_FB), cardiac valve-related mesenchymal cells (Valve_MC), annulus fibrosus fibroblasts (AnnFibr_FB), and interstitial fibroblasts (Int_FB) dispersed across the entire myocardium (Fig. 2E, Ext. Fig. 2B). We also observed a coarse-grained cluster representing epicardium-derived progenitor cells (EPDC) located in the subepicardial domain, with transcriptomic signatures consistent with an epicardial origin and ongoing epithelial-to-mesenchymal cell transition (EMT) (Fig. 2C, E, Ext. Fig. 2B, Suppl. Fig. 5A, D). Furthermore, we recognized an additional mesenchymal cell population resembling a pericyte transcriptomic profile (Peric_MC), and a cluster enriched in cell cycle genes (Prol_FB) (Fig. 2C, E, Suppl. Fig. 2A, Suppl. Table 2).

Our clustering also highlighted an endothelial (PDE4C^{high}_EC) and a fibroblast population (PDE4C^{high}_FB) with overlapping transcriptomic profiles, enriched in several regulators of primary cilia formation and function (*TTC21A*, *ARL13B*, *TULP2*), and the cAMP-signaling-related genes *PDE4C* and *ATF3* (Fig. 2C, E, Suppl. Fig. 2A, Suppl. Table 2). Ciliary cAMP signaling is involved in the differentiation of various cell types[30], and a recent study revealed PDE4C regulating ciliary cAMP signaling in murine kidney cells[31]. By performing immunostaining for the broad cilia marker ARL13B, the endothelial marker UEA I, and PDE4C or ATF3, we observed ciliation, as well as PDE4C and ATF3 protein expression across the entire fetal heart including vessel walls. Furthermore, we found a prominent subcellular localization for both ATF3 and PDE4C in close association with the labelled cilia (Ext. Fig. 2C), in structures consistent with PCNT^{+} basal bodies (data not shown). Thus, our data indicates a unique ciliated cell population, spread across the fetal heart, where cilium-related cAMP signaling may play an important role.

Temporal Changes in Cardiac Cell Composition

Temporal changes of cell state distribution in our dataset followed the main events of early cardiogenesis, including a shift towards mature cardiomyocyte profiles, pronounced expansion of vascular endothelial and mural cell populations associated with coronary vessel formation, a relative shrinkage of the epicardial, endocardial and endocardial cushion-related cellular compartments, and

an expansion of the Schwann cell precursor-glial cell population in the developing innervation (Fig. 2F, Suppl. Fig. 2B). Interestingly, TMSB10^{high}_C_1 and TMSB10^{high}_C_2 clusters, also enriched in *S100A11* and *GNG11*, respectively, showed opposite temporal trends, possibly signaling a shift towards endothelial commitment in these populations (Suppl. Fig. 2B).

Molecular Analysis of Mural Cell States

Beyond the finely resolved and spatially characterized non-mural mesenchymal cell and fibroblast states, our single-cell dataset also included coarse-grained clusters with smooth muscle and pericyte transcriptional characteristics (Fig. 2B-C, Suppl. Fig. 2A, 3A). The two smooth muscle cell populations displayed distinct spatial patterns, one being enriched around the outflow tract and developing great vessels (OFT_SMC), and the other found in a more extended localization within the myocardium with especially high proportion around the large coronary arteries (CA_SMC) (Fig. 2D, Ext. Fig. 7C). Beyond a shared enrichment of genes consistent with smooth muscle identity (*MYH11*, *TAGLN*, *ACTA2*), the two populations featured vastly different transcriptomic profiles (Suppl. Fig. 2A, 3B-C, Suppl. Table 2). As discussed in the 'Assessment of Spatiotemporal Transcriptomic Patterns in the Cardiac Valves, Outflow Tract and Great Arteries' section, OFT_SMCs shared several markers with the adventitial fibroblast population in the same region (OFT_FB), such as previously described regulators of outflow tract development (*MEIS1*[32], *PRDM6*[33,34], *LRP1B*[35]), but displayed selective enrichment of several extracellular matrix components (*ELN*, *FBLN5*) responsible for the elastic properties of the forming great arteries (Suppl. Fig. 7A, Suppl. Table 2). These two gene groups displayed opposite temporal trends in our dataset, consistent with a shift from primary developmental processes towards maturation of the forming great arteries.

CA_SMCs showed a large overlap in their transcriptomic profile, with even similar temporal trends, with the pericyte population (PC), including classical markers of pericytes (*RGS5*, *KCNJ8*, *ABCC9*, *PDGFRB*, *CYGB*, *ENPEP*) (Suppl. Fig. 2A, 3B, Suppl. Table 2). While several of these genes displayed somewhat higher enrichment in the PC population, others involved in local oxygen sensing (*HIGD1B*, *COX4I2*, *NDUFA4L2*) showed mildly stronger enrichment in the coronary smooth muscle cells, in line with these cells' role in mediating hypoxia-induced vasodilation in the coronary vasculature[36] (Suppl. Fig 3B-C, Suppl. Table 2). Pericytes have been previously proposed to serve as precursors for smooth muscle cells in the coronary vasculature, by upregulating *NOTCH3* at arterial remodeling sites in the vicinity of *JAG1*-expressing vascular endothelial cells, upon the onset of blood flow[37]. We observed consistent gene expression patterns in corresponding single-cell clusters with *NOTCH3* showing the highest enrichment in CA_SMCs and PCs, and *JAG1* in CA_SMCs and arterial

endothelial cells (MacrVasc_EC) (Suppl. Fig. 3D) and found high spatial enrichment of the *JAG1-NOTCH3* ligand-receptor pair around the large coronary and great arteries in the Visium dataset (Suppl. Fig. 3E). These observations provide indirect support for a developmental connection between pericytes and coronary artery smooth muscle cells in the human heart. Beyond shared markers, CA_SMCs also showed high expression of transcripts for the adhesion molecule catenin α3 (encoded by *CTNNA3*), the basal membrane component laminin α3 (encoded by *LAMA3*), the Ca^{2+}-binding protein calsequestrin 2 (encoded by *CASQ2*), and gap junction protein-encoding *GJA4* (Suppl. Fig. 3C).

Importantly, the pericyte population shared several highly enriched genes (*PLA2G5, SEMA5A, HEYL*, *ITGA11*, *GUCY1A1*, *CCDC3*) with a fine-grained cell state of the mesenchymal cell-fibroblast population, which we annotated as pericyte-like mesenchymal cells ($Peric\_MC^{fg}$) (Suppl. Fig. 3F). These cell states were mapped to largely complementary tissue regions, with PCs appearing in the compact ventricular myocardium, and $Peric\_MC^{fg}$ cells in the atria and inner layers of the ventricular walls (Fig. 6B). $Peric\_MC^{fg}$ had the highest expression of *APOE*, *ACTA2*, *IGFBP7*, and *MCAM* among the fine-grained mesenchymal cell-fibroblast cell states, consistent with a myofibroblast-like character (Suppl. Fig. 3F, Suppl. Table 6). Cells with largely similar marker profiles have been annotated as myofibroblasts, as well as atrial fibroblasts in recent reports describing the cellular composition of the developing heart[38,39], highlighting the ambiguity regarding the precise identity of this population. Under healthy conditions, the adult human heart is largely devoid of myofibroblasts, which, however, readily differentiate from cardiac fibroblasts upon injury[40], thus the origin and role of cells with similar characteristics in the developing cardiac architecture is yet unclear.

Molecular Analysis of Blood-Related Cell States

In our single-cell dataset, we found a substantial number of blood-related cells, including red blood cells, immature platelets, and immune cells (Fig. 2B-C, Suppl. Fig. 2A, 4A). Since red blood cells and platelets are not considered resident cellular components of the cardiac architecture and appeared to be present in widely varying amounts between experimental samples, we decided to exclude them from downstream analysis, with the exception of cell state deconvolution of the Visium dataset, where the transcriptomic signature of these cell types still needs to be accounted for to obtain reliable results. We identified two coarse-grained clusters (marked as HL_excl_1 and HL_excl_4) dominated by red blood cell-specific transcripts (Suppl. Fig. 4A-B, Suppl. Table 2). The HL_excl_1 cluster showed stronger enrichment of embryonic hemoglobin isoforms (*HBE1*, *HBQ1*, *HBZ*, *HBM*), consistent with an immature embryonic/fetal erythrocyte phenotype, with the HL_excl_4 cluster being characterized

by higher expression of adult-like hemoglobin complex genes (*HBA1*, *HBA2*, *HBB*). Furthermore, HL_excl_1 also included a small subset of cells expressing thrombocyte markers (*PF4*, *GP9*, *PPBP*, *ITGA2B*), presumably representing large embryonic platelets[41], and a small number of cells with granulocyte transcriptional characteristics (*IL5RA*, *FCER1A*) (Suppl. Table 2, Suppl. Fig. 4B).

Myeloid and lymphoid cells were also present in our single-cell dataset (Fig. 2B-C, Suppl. Fig. 2A, 4A). The majority of myeloid cells featured markers consistent with yolk sac origin, such as *CX3CR1* and *CSF1R*, while a smaller population expressed *FLT3*, highlighting the gradual appearance of immune cells originating from the fetal liver[42] (Suppl. Fig. 4C, upper panels). The distribution of these markers also aligned with the positions of $CCR2^-$ and $CCR2^+$ subpopulations within the myeloid compartment, respectively (data available in our interactive viewer). These results are consistent with observations in the adult heart, where *CCR2* expression was proposed to separate a macrophage population derived from definitive hematopoietic progenitors, constantly being renewed by monocyte recruitment, from self-renewing, tissue-resident macrophage population derived from the yolk sac under the fetal period[43]. The lymphoid population also appeared to be a mixture of cells with B-, T-, and NK-cell characteristics based on the expression pattern of *JCHAIN*, *CD3E* and *KLRB1* within the population (Suppl. Fig. 4C, lower panels). While both the myeloid and lymphoid populations showed dispersed patterns throughout the tissue, co-detection analysis highlighted their highest spatial overlap with cells in the epicardial and subepicardial regions and the adventitia of the great vessels (Suppl. Fig. 4D). These results align with the previously proposed seeding path for yolk sac-derived immune cells in mice, first populating a niche within and under the epicardial layer[44]. In addition, they provide indirect support to reports describing the central role of myeloid cells in the formation of coronary arteries and lymphatic vessels in the developing heart[45,46], by placing this cell type in the vicinity of such vessel structures (Ext. Fig. 7C).

While we did not observe any distinct single-cell state with characteristics of lymphoid stromal cells, we captured high spatial enrichment of *CCL19*, a consensus marker of these cells, in several Visium sections, in concise segments within the subepicardium and adventitia of the great vessels (Suppl. Fig. 4E). These areas showed a markedly high predicted proportion of lymphoid cells (LyC), and a close spatial association with lymphatic endothelial cells (LEC), supporting that the related tissue structures are, in fact, lymph nodes of the developing cardiac lymphatic system.

Spatial Heterogeneity of Fine-Grained Mesenchymal Cell-Fibroblast Cell States

Fine-grained clustering of the mesenchymal cell-fibroblast cellular subset delineated spatially distinct cell states (Fig. 6A, Ext. Fig. 6A-B, Suppl. Table 6), including mesenchymal components of the developing cardiac valves, discussed in detail in the main text (Fig. 6E-G, Ext. Fig. 6F-G).

Additionally, we recognized two adventitial fibroblast states (Adv_FB_1-2), with Adv_FB_1 outlining the wall of the outflow tract and great arteries, and Adv_FB_2 also appearing around large coronary arteries (Fig. 6B). The Peric_MC^{fg} mesenchymal cell population resembling the pericyte transcriptional signature, as discussed above, was traced to the innermost layer of the ventricular myocardium and to the atrial walls, to positions complementary to the genuine pericyte population (Fig. 6B, Ext. Fig. 6A-B). This localization is suggestive of a potential endocardial origin, which has recently been proposed for a pericyte-like cardiac mesenchymal cell state in mice[47].

Furthermore, we identified two fine-grained populations representing early forms of epicardium-derived progenitor cells (EPDC), based on their predicted positions in the subepicardium at the atrioventricular groove (EPDC_1) and the heart surface (EPDC_2), and combined enrichment of the epicardial marker *WT1* and EPDC marker *TCF21*[48] (Fig. 6C, Ext. Fig. 6B, E, G; further discussed in the 'Transcriptomic Heterogeneity in Epicardium-Related Cell States' segment). We also found various cell states consistent with an interstitial fibroblast phenotype, characterized by high *CD34* expression and gradual temporal expansion from the epicardium to the outer (Int_FB_1) and inner (Int_FB_2) layers of the ventricular wall, or located in the subendocardium and the atrioventricular region (Int_FB_3) (Fig. 6D, Ext. Fig. 6B). Int_FB_1 and Int_FB_2 exhibited the highest *TCF21* expression in our dataset, indicating advanced transition from an epicardial progenitor state towards fibroblast identity (Ext. Fig. 6B, Suppl. Fig. 5E). Notably, we also recognized a distinct cell state with robust *CD34* expression, dynamic temporal expansion, and a transcriptomic profile (*CLEC3B*, *ROBO2*, *SEMA3C*, *ADAMTSL1*, *CCN3*, *SOX9*, *DLK1*) resembling fibro-adipogenic progenitors (FAP) (Ext. Fig. 6A-C). Many of these markers appeared closely associated in a gene regulatory network defined by three transcription factors enriched in this population: while *ZBTB16* promotes white and brown adipogenesis[49], *KLF2* and *GLIS3* block further adipogenic differentiation of preadipocytes[50,51], consistent with the progenitor characteristics of this cell state (Ext. Fig. 6D). Spatial mapping showed enrichment of this population near EPDC_1 cells in the atrioventricular groove, reflecting the predominant localization of the primordial epicardial adipose tissue (EAT), as well as a presumed epicardial origin of these cells, besides more dispersed occurrence in the ventricular wall (Fig. 6E, Ext. Fig. 6E). FAPs have been proposed as potential sources of fatty-fibrous tissue deposits, a histological hallmark in arrhythmogenic right ventricular cardiomyopathy (ARVC)[52].

Importantly, we found two ARVC-associated desmosome-encoding genes (*PKP4*, *DSC2*) enriched in the FAP cell state, drawing a potential connection towards the pathogenesis of this disease[53] (Suppl. Table 6). Additionally, we recognized a third *WT1*-enriched cell state, also expressing high levels of *CACNA2D3* and *BRINP3*, localized in a distinct layer between the atria and ventricles, outlining the external parts of the fibrous cardiac skeleton (AnnFibr_FB) (Fig. 6E, Ext. Fig. 6A, E, G), beside a fourth population marked by inflammatory gene expression (Infl_FB) (Ext. Fig. 6A).

Notably, we also identified a fibroblast population (CALN1^{high}_FB) featuring high expression of characteristic nodal cell-enriched genes (*CALN1*, *SHOX2*, *CNTN5*), as well as the angiotensinogen-encoding *AGT*, a central component of local renin-angiotensin circuits in nodal tissue (Ext. Fig. 6A, Suppl. Table 6). The latter observation highlights this population as potential developmental equivalents of sinoatrial and atrioventricular node-resident fibroblasts, supported by cell state mapping (Fig. 7B).

Transcriptomic Heterogeneity in Epicardium-Related Cell States

Several studies have described molecular heterogeneity within the developing epicardial compartment. However, human data related to this question are still scarce and partly derived from the analysis of iPSC-derived epicardioids[54–56].

In the developing heart, the epicardium serves as the source of a wide range of mesenchymal cells, including cardiac fibroblasts, pericytes and smooth muscle cells, while their potential contribution to the endothelial and cardiomyocyte compartments, proposed by several animal studies under development and in tissue repair[57–61], is still largely debated. To generate mesenchymal cells, epicardial cells undergo epithelial-mesenchymal transition (EMT), during which they lose their epithelial characteristics, migrate into the subepicardial layer, and become multipotent intermediates called epicardium-derived progenitor cells (EPDCs), which subsequently give rise to the previously mentioned mesenchymal cell types. Many components of the EMT machinery are known, but because of the gradual nature of this process, there is no clear-cut molecular signature that defines the limit between epicardial and EPDC identities.

Epicardial heterogeneity discussed in previous human studies[54,55] was broadly related to the extent and activity of the EMT process in the analyzed epicardium-related samples. A recent report[54], comparing developmental and adult epicardial cells in single-cell datasets of dissociated whole heart tissue, identified three subpopulations within their epicardial compartment, including a mesothelial, a fibroblast-like, and a proliferating subpopulation, and highlighted temporal shifts, with the two latter

populations disappearing in adult samples. Another work[55] analyzed isolated human fetal subepicardial samples and identified two epithelial and three mesenchymal populations, however, molecular heterogeneity between clusters within the two groups was limited. These findings suggest that epicardial and epicardium-derived progenitor cells (at least in the earliest stages of their transition) were co-analyzed in these investigations. Taking this into consideration, it is plausible that inconsistencies between studies arise from shifts of cells representing the earliest stages of the EMT process between the epicardial and EPDC clusters. This is further supported by the fact that none of these studies define clear marker sets for the observed subpopulations and rather describe them based on the relative and gradual enrichment of genes related to epicardial vs. mesenchymal identities. Furthermore, collection and processing of the analyzed heart samples might also differ (such as entire hearts vs. enriched epicardial and subepicardial tissue; processing with or without cell sorting; etc.), which might also contribute to the overall variability between individual datasets, and thus to the definition of different clusters. Finally, it is challenging to differentiate between cellular heterogeneity inherently present in the resting epicardium and that due to a commenced differentiation process.

In agreement with these findings, we also observed an epicardial (EpC) and a separate epicardium-derived progenitor cell (EPDC) coarse-grained cluster in our dataset (Fig. 2B-C, Suppl. Fig. 2A, 5A, Suppl. Table 2). Subsequent subclustering of non-mural mesenchymal cell-fibroblast subset allowed us to further refine cells with EPDC transcriptional characteristics, outlining two distinct fine-grained cell states, EPDC_1 and EPDC_2 (Fig. 6A, Ext. Fig. 6A, Suppl. Table 6). While EpCs showed gene enrichment consistent with a mesothelial cell identity (*ITLN1*, *SBSPON*, *EZR*, *UPK3B*, *TNNT1*, *KRT19*, *BNC1*, *MSLN*, *PRG4*), EPDC_1 and EPDC_2 cell states also expressed mediators of the EMT process (*SPARC*, *POSTN*, *SNAI2*) and other relevant transcripts associated with epicardium-derived mesenchymal identity, highlighted by other studies[39,54,55] (*MMP11*, *MOXD1*, *CCBE1*, *VEGFC*, *FNDC1*, etc.), beyond shared enrichment of the classically used epicardial markers *WT1* and *TBX18* (Suppl. Fig. 5B-C).

Notably, cell state mapping not only supported these annotations but also revealed spatial variations within the subepicardium between the two fine-grained EPDC cell states. We found EPDC_1 being concentrated at the atrioventricular groove and EPDC_2 around the ventricular surface, potentially indicating regional differences in the mesenchymal transition process, while EpCs appeared in their expected position, on the outer lining of the heart (Fig. 2D, 6C). Furthermore, while some epicardial markers exhibited relatively consistent expression throughout the investigated time frame in the EpC state, we observed a gradual temporal increase in others (*MSLN*, *C3*, *PRG4*), suggesting that these markers play a defining role in mature mesothelial characteristics (Suppl. Fig. 5D).

When assessed across all fine-grained cell states, we found residual expression *WT1* in the EPDC_1-2, Infl_FB and AnnFibr_FB, and *TBX18* in several other fine-grained mesenchymal cell and fibroblast populations (including FAPs and Int_FB_1-2 cells), providing support to a plausible developmental connection between these cell states and the epicardium (Suppl. Fig. 5E). This observation aligns with the widely accepted view that most cardiac fibroblasts are derived from an epicardial origin, with fibroblasts of proposed endocardial or neural crest origin typically localizing to largely distinct cardiac regions (valves, parts of the subendocardium, ventricular septum). In line with a recent report[38], we found the highest enrichment of *TCF21*, a previously proposed marker of epicardial and epicardium-derived progenitor cells, in the FAP and Int_FB_1-2 populations, supporting the notion that this gene's expression is associated with a more differentiated fibroblast identity in the developing human heart (Suppl. Fig. 5E).

Assessing the presence of a potentially epicardium-derived cardiomyocyte subpopulation in the developing heart was not feasible from the analyzed single-cell transcriptomics dataset, due to ambiguity regarding the relevant age range, overall low number of epicardial cells, and the high level of heterogeneity within the cardiomyocyte compartment. Importantly, cardiomyocyte-related gene expression in epicardial subsets[60] is not conclusive of actual cardiomyogenic differentiation, and previously published fate-mapping studies have been broadly challenged based on presumed leakiness of selected targeting approaches (such as WT1- or TBX18-based reporter gene expression[57–59], or pericardial injection of TAT-Cre recombinase[61]). Thus, and similarly to the other human studies mentioned, we could not observe any clear sign of a potential epicardial contribution to the cardiomyocyte compartment in our dataset.

Temporal Gene Expression Changes in Coarse-Grained Endothelial Cell and Mesenchymal Cell-Fibroblast Clusters

Temporally resolved differential gene expression analysis across coarse-grained single-cell clusters provided insight into relevant molecular transitions in the endothelial (Suppl. Fig. 6A-B) and mesenchymal cell-fibroblast (Suppl. Fig. 6A, C) populations, beyond highly enriched markers calculated from the integrated dataset (Suppl. Fig. 2A, Suppl. Table 2).

Between the macro- (MacroVasc_EC) and microvascular (MicroVasc_EC) endothelial cell clusters of the developing coronary vasculature, we observed an opposite temporal pattern for *CLDN5*, *PRND*, and *IGFBP3* gene expression, implicated in the regulation of endothelial barrier function and angiogenesis, suggesting diverging characteristics related to these cellular functions from an early developmental stage. These populations displayed similar gradual enrichment of many shared marker

genes over time, beyond some more specifically distributed and gradually enriched transcripts (*GJA5*, *GJA4*, *EYS*, *DKK2* in the MacroVasc_EC, and *CDH13*, *APLNR* in the MicroVasc_EC cluster) (Suppl. Fig. 6B_A'). On the other hand, we detected strong selective enrichment of several marker genes of endocardial (Endoc_EC) (*PCDH7*, *TMEM100*, *CLEC3B*) and endocardial cushion-related endothelial cell clusters (EndocCush_EC) (*APCDD1*, *LTC4S*) already in the earliest analyzed age group (5.5-6 pcw), underscoring an even earlier specification between these two populations (Suppl. Fig. 6B_B'). The lymphatic endothelial cells (LECs) showed gradually increasing expression of consensus markers (*FLT4*, *NTS*, CCL21, *LYVE1*, *TFF3*, *MMRN1*) (Suppl. Fig. 6B_C').

In the mesenchymal cell-fibroblast subset, we focused our analysis on the interstitial fibroblast (Int_FB) and pericyte-like mesenchymal cell (Peric_MC) populations, which appeared to be the dominant mesenchymal cell components of the ventricular and atrial walls, respectively (consistent observation on the fine-grained level are presented in the niche network in Fig. 7A). In line with our results on the fine-grained clustering level, we found the highest enrichment of *TCF21* in the Int_FB coarse-grained population across the entire investigated time frame. At the same time, several consensus markers of cardiac fibroblasts (*DCN*, *LUM*, *C7*, *ABCA9*) showed gradual increase in expression in this population, along with *ROBO2*, a highly enriched gene in the fine-grained fibro-adipogenic progenitor-like (FAP) cell state identified in our dataset. This molecule is a component of the SLIT-ROBO signaling pathway, which is increasingly recognized in the modulation of fibrotic responses in various organs[62–64] (Suppl. Fig. 6C_D').

The Peric_MC population, on the other hand, showed marked, although gradually decreasing enrichment of *THY1*, another proposed cardiac fibroblast marker, compared to other coarse-grained clusters of the mesenchymal cell-fibroblast subset. Importantly, the expression of this gene appeared to be highest in the pericyte population throughout the investigated timeframe, adding to several transcriptomic similarities between the two cell states (Suppl. Fig. 6C_D'; also highlighted for the fine-grained Peric_MC^{fg} population in Suppl. Fig. 3F). In terms of extracellular matrix components, the Int_FB and Peric_MC clusters followed largely similar temporal trends and expression levels (*COL6A3*, *COL6A6*, *COL21A1*, *OGN*). On the other hand, *DCN* and *LUM*, encoding the small leucine-rich repeat proteoglycans decorin and lumican, showed higher abundance in the Int_FB, and *TNC*, encoding tenascin C, in the Peric_MC population, highlighting compositional differences between the developing atrial and ventricular interstitial extracellular matrix (Suppl. Fig. 6C_D').

Temporally differentially expressed genes in the mesenchymal cell population located in the outer annulus fibrosus (AnnFibr_FB) showed substantial overlaps with other mesenchymal cell

clusters, such as *BRINP3* and *MAGI2* (also gradually downregulated in the Valve_MC cluster) or *FBLN1* (showing similar enrichment over time in the EPDC and OFT_FB clusters) (Suppl. Fig. 6C_E').

Temporal gene expression patterns related to the OFT_FB, Valve_MC, OFT_SMC, CA_SMC and PC coarse-grained clusters are presented in Supplementary Figure 7A-C and Supplementary Figure 3A-F, and discussed in the 'Molecular Analysis of Mural Cell States' and 'Assessment of Spatiotemporal Transcriptomic Patterns in the Cardiac Valves, Outflow Tract and Great Arteries' segments.

Assessment of Spatiotemporal Transcriptomic Patterns in the Cardiac Valves, Outflow Tract and Great Arteries

The Visium dataset in this study provides comprehensive spatial gene expression information from various stages of early heart development, enabling the exploration of spatiotemporal expression patterns during cardiogenesis. However, analyzing gene expression independently within spatial clusters or regions is not advisable due to several technical challenges, such as differences in cell type composition and density per spatial spot, and discrepancies between sampled anatomical regions. A more robust approach involves integrating the spatial component through spatially aware annotation of cell states identified in the independent single-cell transcriptomic dataset. By selecting the dominant cellular components of regions of interest, these can be assessed through time-resolved differential gene expression analysis to uncover relevant molecular transitions, which can then be spatially validated in the Visium dataset in sections representing consecutive developmental stages. This strategy leverages the overall rich single-cell RNA expression data, providing a more detailed assessment than independent analysis of spatial datasets would allow.

Accordingly, we assessed temporal transcriptional changes in selected regions of interest by utilizing results of time-resolved differential gene expression analysis across age-subsetted (5.5-6 pcw, 7-8 pcw, 9-11 pcw, and 12-14 pcw) subpopulations of all coarse-grained single-cell clusters (Suppl. Fig. 7A). We deduced spatiotemporal patterns related to the outflow tract and great arteries, as well as the cardiac valves, by investigating highly expressed, temporally differentially expressed genes identified in the OFT_FB, OFT_SMC, and Valve_MC single-cell clusters (Suppl. Fig. 7A), which show highly specific localization in these regions (Fig. 2D, Ext. Fig. 2B). In the OFT_FB and OFT_SMC populations, we found several previously proposed mediators of outflow tract development, potentially related to the neural crest origin of certain cellular components of this region, such as *PRDM6*[33,34], *MEIS1*[32], *LRP1B*[35], showing decreasing expression over time. Simultaneously,

key determinants of the developing great vessels' elastic properties (*ELN*, *PI15*) became gradually enriched in OFT_SMCs throughout the investigated timeframe, with a similar trend in non-canonical NOTCH ligand *DLK1* in OFT_FBs. In the Valve_MC cluster, we observed a clear temporal downregulation of *SEMA3D* and an upregulation of *COL12A1*, suggesting distinct roles for these molecules in the early formation and subsequent maturation of cardiac valves[65,66]. Importantly, we spatially validated the observed expression patterns in sections included in our Visium dataset (Suppl. Fig. 7B-C), confirming the reliability of our approach to deduce spatiotemporal molecular signatures from the temporal analysis of spatially annotated cell states. This strategy can be refined by applying the same temporal differential gene expression analysis on finely resolved single-cell clusters with distinct spatial distributions, as outlined by the predicted cellular composition of cardiac compartments and niches (Fig. 7A). However, low cell numbers in certain time-resolved clusters can be a limiting factor in this type of analysis.

SUPPLEMENTARY DISCUSSION 2

In-depth comparison with Farah et al., 2024, Nature[39]

(Figures generated for this analysis are highlighted in bold in the following discussion to distinguish them from figures associated with the main article.)

Beyond our current study, and its predecessor by Asp *et al.*[48], which presented the first spatiotemporal atlas of early human heart development, the recent article by Farah *et al.* represents the latest published investigation of human cardiogenesis on the whole organ level, including spatial omics-based approaches.

Comparison of Datasets, Sample and Data processing

While the conceptual similarity of integrating spatial transcriptomics data in downstream analysis of cellular and molecular events of heart development connects our work to Farah et al., there are several important differences between the collected datasets (**Table 1** of Suppl. Discussion 2).

In their study, Farah et al. analyze a substantial single-cell dataset (142,946 cells) over a later developmental window (9th-16th postconceptional weeks), with a spatial component derived from a smaller sample size (4 sections from 2 hearts), collected at two close time points of the second trimester (12-13th and 15th postconceptional weeks). Additionally, the authors used a targeted spatial transcriptomics method (MERFISH) to spatially validate predefined cell states using a number of selected markers (238 target genes). While excellent for validation, this approach restricts further exploration of spatiotemporal molecular and cellular patterns, mainly due to probe multiplexing limitations or unclear marker profiles of the targeted cell populations.

In our study, we present an untargeted Visium spatial dataset and analysis strategy that supports both hypothesis- and data-driven explorative analysis. Although the Visium analysis offers lower spatial resolution (55 µm), it generates a comprehensive database of the spatial expression patterns of over 16,800 genes without prior selection, across a large and diverse collection of samples (38 tissue sections from 16 hearts, covering 69,114 spatially barcoded tissue spots), and an earlier developmental window which aligns better with major cardiogenic events of the first trimester (6th-12th postconceptional weeks). Our dataset is further supported by a meaningful integration with a largely age-matched (5.5^{th}-14th postconceptional weeks) independent single-cell sequencing dataset (76,991 cells), enabling the discovery of even minor, spatially distinct developmental cell states without predefined marker profiles. Similarly to Farah *et al.*, we also utilized a targeted spatial approach, *in*

situ sequencing, to generate a sizable complementary, high-resolution dataset as an independent validation for the spatial expression patterns of 150 selected genes, including markers of various novel developmental cell states and regulators of cardiogenesis.

**Study**		**Farah *et al.*, 2024, Nature**	**Lázár *et al.*, 2025, Nature Genetics**
**Single-cell RNA-sequencing dataset**			
**Technology**		10x Chromium	10x Chromium
**Size**	# Hearts	8 (selected pieces)	15 (whole)
	# Cells	142,946	76,991
	# Clusters	75 (w/ RBCs)	72 (w/o RBCs)
**Developmental stage**		9-16 pcw	5.5-14 pcw
**Untargeted spatial transcriptomics dataset**			
**Technology**		-	10x Visium
**Size**	# Hearts	-	16
	# Sections	-	38
	# Spots	-	69,114
	# Clusters	-	23 clusters, 14 regions
**Developmental stage**		-	6-12 pcw
**Spatial resolution**	Spot ⌀	-	55 µm
	Spot ↔	-	100 µm
**Gene resolution**		-	Whole transcriptome
**Targeted spatial transcriptomics dataset**			
**Technology**		MERFISH	ISS
**Size**	# Hearts	2	4
	# Sections	4	9
	# Clusters	27	(Mapped coarse-grained clusters)
**Developmental stage**		12-13, 15 pcw	6.5-11.5 pcw
**Spatial resolution**		Cellular	Cellular
**Gene resolution**		238 target genes	150 target genes

Supplementary Discussion 2 - Table 1. Comparison between datasets presented by Farah *et al.* and the current study (*RBC - red blood cell).

Considering the different nature of the spatial transcriptomics datasets included in the two studies, we focused our comparison on the single-cell RNA-sequencing datasets. Importantly, we observed several differences in the processing of the collected heart samples (**Table 2** of Suppl. Discussion 2), as well as the processing and filtering of the obtained sequencing data (**Table 3** of Suppl. Discussion 2) that might have a fundamental impact on the presented analysis results and can account for some of the observed discrepancies.

**Study**		**Farah *et al.*, 2024, Nature**	**Lázár *et al.*, 2025, Nature Genetics**
**Method of pregnancy termination (medical/surgical)**		Not disclosed	Medical
**Dissociation**	Enzymes	Type IV collagenase	Type II collagenase + DNase I
	Duration	60 min	45-150 min
**Resuspension buffer**		EBSS	PBS + 5% FBS
**Cell enrichment**		Filtering + centrifugation (+ gradient centrifugation)	Cell sorting

Supplementary Discussion 2 - Table 2. Comparison in the collection and processing of developmental heart samples for single-cell RNA-sequencing.

**Study**	**Farah *et al.*, 2024, Nature**	**Lázár *et al.*, 2025, Nature Genetics**
**Sequencer**	HiSeq4000	NovaSeq
**Sequencing depth**	Min. 65,000 reads/cell	100,000-250,000 reads/cell
**Cellranger version**	v.3.0.1	v.4.0.0
**Reference genome**	hg38	GRCh38.p13 genecode 35 (with read filtering[67])
**Filtering approach**	Keep cells with >1,000 unique genes and <30% mitochondrial transcripts	Keep cells with >250 unique genes, <30% mitochondrial transcripts, >3% ribosomal transcripts (and RBSs with <3% ribosomal and >10% hemoglobin transcripts)

Supplementary Discussion 2 - Table 3. Differences in the generation and processing of the single-cell RNA-sequencing dataset.

Farah *et al.* do not discuss the details of sample collection, such as if the analyzed fetuses were obtained through surgical or medical abortions, and time limits from the procedure for inclusion in the study. The authors opted for dissecting the collected hearts into intact cardiac chambers and the interventricular septum to increase the coverage of smaller structural components instead of processing entire heart samples, biasing the observed cell state proportions compared to the physiologically occurring ratios. The two datasets also differ in sequencing depth, used analysis software version, read filtering approach, as well as filtering criteria for high-quality cells, introducing further discrepancies between the datasets.

Comparison Strategy for Cellular Subsets

The comparison between the full datasets and the different subsets was carried out using the R package Seurat in the following way: features used for integration were selected using SelectIntegrationFeatures prior to the merging of the datasets. After merging, the datasets were renormalized, followed by scaling and centering, PCA, and integration using Harmony. The results were visualized using UMAPs, separated by dataset and grouped according to original annotations, allowing for the assessment of integration of corresponding labels between the datasets. To confirm the presence of certain fine-grained cell states identified in our study and not accounted for by Farah *et al.*, we performed label transfer on the subsetted data, by projecting our annotations onto the equivalent subsets using FindTransferAnchors followed by TransferData. Agreement in gene expression between clusters of the two datasets was assessed based on the distribution of consensus cell type markers, highly enriched genes determined in the individual datasets, or by using the FindAllMarkers function in the relabelled populations of Farah *et al.*

Integration of Major Cell Populations

Applying the data integration strategy described above to all cells in the two datasets, we observed a good agreement, evidenced by the consistent positions of most major cell classes as defined by Farah *et al.*, and the corresponding populations of merged coarse-grained clusters in our dataset (**Fig. 1A-C** of Suppl. Discussion 2). Notably, this level of clustering did not account for pericytes in the dataset of Farah *et al.* The pericyte cluster identified in our dataset appeared partly associated with the smooth muscle cell (SMC) and partly with the fibroblast (Fibro) populations of Farah *et al.* on the integrated UMAP, consistent with the mural cell characteristics of this cell type (**Fig. 1B-C** of Suppl. Discussion 2).

Farah *et al.* did not identify counterparts of the TMSB10^{high}_C_1 and TMSB10^{high}_C_2 cell populations; however, these clusters appeared in separate positions on the integrated UMAP, similar to their positions in our independent analysis (**Fig. 1B-C** of Suppl. Discussion 2). The smooth muscle cell population from our dataset appeared in two separate positions on the integrated UMAP as well, likely corresponding to distinct clusters related to the outflow tract and great arteries, and to the coronary arteries. In contrast, the SMC cell class from the dataset of Farah *et al.* appeared in a single position, presumably consistent with cells from the latter structures (**Fig. 1B-C** of Suppl. Discussion 2). This discrepancy might be due to the preselection of dissected tissue pieces for the single-cell RNA-sequencing analysis by Farah *et al.*, where the coverage of the great arteries was not discussed.

Integration of the Cardiomyocyte Subsets

Among the cellular subsets, cardiomyocytes showed the least consistent transcriptomic profiles between the two datasets. The cardiomyocyte clusters identified in our study featured markedly lower QC values in comparison to other cellular subsets (Suppl. Fig. 16F, data also available in our interactive viewer), potentially signaling lower tolerance to the experimental protocol. Therefore, discrepancies between the datasets might stem from the differences in filtering parameters based on QC metrics between the two studies (cells with <250 unique genes vs. <1000 unique genes filtered out in our analysis vs. Farah *et al.*), making it possible that equivalents of a substantial proportion of cardiomyocytes analyzed in our study were excluded from the presented dataset of Farah *et al.* The qualitative difference between the datasets was also reflected in the results of our attempt at label transfer between the datasets, using our cluster annotations as reference. In this analysis, the bulk of the cells with an original atrial identity received the Immat_CM_2 label, and the bulk of the ventricular cardiomyocytes were reannotated as vCM_6 (data not shown), obscuring molecular heterogeneity defining cluster identities within our own dataset.

Nevertheless, integration between the cardiomyocyte subsets (**Fig. 2A** of Suppl. Discussion 2) confirmed the separation between clusters with ventricular and atrial identities, with the latter being further separated according to left and right sides in both datasets (**Fig. 2B-C** of Suppl. Discussion 2). In their spatial analysis, Farah *et al.* also distinguished between left and right ventricular cardiomyocyte populations, and compact and trabecular subpopulations within, based on the enrichment of *SLC1A3* and *PRRX1*, and *HEY2* and *IRX3*, respectively (although the precise relation of these populations to the identified ventricular cardiomyocyte single-cell clusters remained unclear). In our fine-gained ventricular cardiomyocyte clusters, vCM_4 and vCM_5 displayed relative enrichment on the left and right sides (cell state mapping results available in our interactive viewer), respectively, with consistent distribution of *SLC1A3* and *PRRX1*, although neither of these genes appeared to be specific for these two clusters within our cardiomyocyte subset (Suppl. Table 3). The populations marked as Early_vCM and Late_vCM of Farah *et al.* appeared interspersed with the merged vCM and Immat_CM (marking immature cardiomyocytes) populations of our dataset (**Fig. 2B-C** of Suppl. Discussion 2), suggesting that these categories are not distinct and are only partially explained by temporal factors. The atrial cardiomyocyte population from Farah *et al.* also appeared separated into two major areas on the integrated UMAP, with one population overlapping with Immat_CMs from our dataset, potentially highlighting cells with less mature characteristics (**Fig. 2B-C** of Suppl. Discussion 2).

Within the cardiomyocyte subset, we focused our investigation on components of the cardiac pacemaker-conduction system (CPCS), thus we aimed to confirm the agreement of these cell states

between the two datasets. We found consistent enrichment of relevant marker genes (defined based on the independent analysis of our dataset) between the SAN_CM and the IFT-like_ncCM (C20), the AVN_CM and the AVC-like_ncCM (C21), and the AVB-BB_CM and Purkinje_CM and the C3 clusters, identified in our study and by Farah *et al.*, respectively (**Fig. 3A** of Suppl. Discussion 2). Despite the challenges with label transfer in this subset, we still identified small cell populations in the dataset of Farah *et al.* relabeled as SAN_CM, AVN_CM and AVB-BB_CM, which mostly originated from the above-mentioned corresponding clusters from the original analysis of Farah *et al.* (**Fig. 3B** of Suppl. Discussion 2). Interestingly, with this approach we could not identify any distinct population consistent with Purkinje fibers (PF_CM) in the dataset of Farah *et al.*, and several markers of this population (*CSMD1*, *SGCD*, *PLXNA4*) appeared to be only weakly expressed in the related C3 cluster (**Fig. 3A** of Suppl. Discussion 2). In their spatial analysis, Farah *et al.* only defined a mixed His-Purkinje ncCM population on the whole section level and separate His bundle- and Purkinje fiber-related populations with further subclustering of the ventricular cellular components, which they observed almost exclusively in the left ventricles. Meanwhile, we could discern distinct AVB-BB_CM and PF_CM clusters in our single-cell dataset and confirm their presence in their expected positions in the subendocardium of the interventricular septum and free ventricular walls on both sides, while also observing higher predicted proportions of PF_CMs in the left ventricle. Importantly, Farah *et al.* presented the IFT-like_ncCM, AVC-like_ncCM and the vCM-IVS-His MERFISH profiles in consistent spatial localization to the ones identified in our study, providing further validation for our unbiased cell state mapping results (data available in the interactive viewers of the two studies).

We also found the vCM_1 cell state identified in our study to be highly consistent with the C4 single-cell cluster of Farah *et al.*, based on several shared markers (*XPO4*, *TNFRSF19*, *CNN1*, *CRABP2*) and supported by label transfer (**Fig. 3A-B** of Suppl. Discussion 2). Farah *et al.* mapped this $CNN1^{+}$-$CRABP2^{+}$ cell population to the atrioventricular region, including valve leaflets, in a side-specific manner, consistently annotated them as vCM-LV/RV-AV, and proposed a potential role for these populations in valve development. We also traced our vCM_1 cell state to consistent regions in the heart tissue across several developmental stages, and highlighted its transcriptional similarities and close spatial association to conductive cardiomyocyte states (AVN_CM and AVB-BB_CM) in the atrioventricular plane, proposing a potential role for these cells in the development of CPCS components in this region (data available in the interactive viewers of the two studies). Importantly, a substantial proportion of the relabeled vCM_1 cells in the dataset of Farah *et al.* originated from the AVC-like_ncCM (C21) cluster, highlighting some ambiguity related to the precise identities of these cell states (**Fig. 3B** of Suppl. Discussion 2). This might also explain the absence of some important

vCM_1 markers (*TENM3, KCNIP4*) from the C4 cluster of Farah *et al.* (**Fig. 3A** of Suppl. Discussion 2), related to the presumed connection of this population to conductive cardiomyocyte states.

Integration of the Endothelial Cell Subsets

The endothelial cell subsets appeared well-integrated on the common UMAP (**Fig. 4A** of Suppl. Discussion 2), based on the interspersed appearance of corresponding populations of the two datasets (**Fig. 4B-C** of Suppl. Discussion 2). The endocardial and vascular endothelial cells of the two datasets were consistently separated into two major populations on the integrated UMAP, according to their vastly different transcriptomic profiles. The vEndocardial population of Farah *et al.*, marking endocardial cells in the ventricles, displayed large overlap and shared marker genes (*NPPC*, *PLAC9*) with the Endoc_EC_1 and Endoc_EC_2 clusters in our dataset (**Fig. 5A** of Suppl. Discussion 2). These clusters also displayed spatial enrichment in the ventricles and higher co-detection scores with components of the ventricular conduction system components, highlighted in our cellular niche graph (Fig. 7A). The atrial aEndocardial population showed similar alignment and shared markers (*COLEC11*, *PROCR*, *SAT1*, *COL3A1*) with the Endoc_EC_3 and Endoc_EC_4 clusters, corresponding to atrium-enriched endocardial cell populations in our dataset (**Fig. 5A** of Suppl. Discussion 2). Importantly, the E5 cluster from Farah *et al.* also appeared to overlap with the venous endothelial cell population (Ven_EC) identified in our dataset (**Fig. 4B-C** of Suppl. Discussion 2). A consistent population, not annotated by Farah *et al.*, was also revealed through label transfer within the E5 cluster of their dataset (**Fig. 5B-C** of Suppl. Discussion 2). Similarly, Farah *et al.* did not further explore the cellular heterogeneity within the endocardial cushion-related endothelial cell population, such as the presence of distinct valve endothelial cell states on opposite sides of the atrioventricular and semilunar valves (marked as OF_VEC and IF_VEC in our dataset), as well as atrial septum-associated endothelial cells (marked as AtrSept_EC in our dataset). Based on the integrated UMAPs and relative enrichment of relevant markers (*HAPLN1*, *COL26A1*, *TSPAN8*), we expected these cell states to be concealed within the E3 and E4 clusters of Farah *et al.* (**Fig. 4B-C** of Suppl. Discussion 2). By label transfer, we could confirm the presence of cells corresponding to all three endocardial cushion-related populations in the dataset of Farah *et al.*, and observed consistent marker profiles of these clusters between the two datasets (*MSX1*, *CCDC80*, *IL1RL1* for AtrSept_EC; *KISS1*, *IFI27*, *S100A6* for IF_VEC; *RGS5*, *BMP4*, *COL26A1* for OF_VEC), based on differentially expressed genes calculated for the relabeled populations (**Fig. 5B-C** of Suppl. Discussion 2).

The vascular endothelial cell populations of the two datasets also showed overall good agreement, but similarly to the endocardial compartment, our dataset provides higher granularity in

terms of annotated cell states. The BEC-Arterial population (consistent with the E7 cluster of Farah *et al.*) aligned with the Art_EC_1, Art_EC_2 and Arteriol_EC populations within our dataset, distinguishing distinct endothelial cell states of consecutive vascular regions from large arteries to the initial segment of the microvasculature (**Fig. 4B-C** and **5A** of Suppl. Discussion 2). The BEC-Capillary population (consistent with the E8 cluster) showed substantial overlap with the Cap_EC_1, and the BEC-Venous population (consistent with the E9 cluster) with the Cap_EC_2 clusters in our dataset on the integrated UMAP (**Fig. 4B-C** of Suppl. Discussion 2). Importantly, the E9 cluster strongly expressed several consensus capillary markers (*RGCC*, *CA4*) and contained only a small cell population with genes associated with postcapillary venule endothelial cell identity (*ACKR1*, *SELE*), also supported the transfer of the Venul_EC label from our annotation to these cells (**Fig. 5B-C** of Suppl. Discussion 2). As discussed above, another cell population with venous character identified in our study, Ven_EC, appeared interspersed with the E5 atrial endocardial cell cluster. These observations make the annotation of the E9 cluster of Farah *et al.* as venous cells ambiguous. With our label transfer strategy, we found the highest diversity of source clusters for the relabeled Cap_EC_2 population (data not shown), also highlighting discrepancies between the transcriptomic profiles of the E9 cluster of Farah *et al.* and the Cap_EC_2 cluster in our dataset. Lymphatic endothelial cell clusters showed good agreement between the two datasets, likely due to their distinct transcriptomic profiles compared to other endothelial subtypes. We also observed shifts between the clusters marking proliferating and capillary endothelial cells between the two datasets, which is likely due to the gradual appearance of the cell cycle-specific transcriptional program, and therefore likely not signaling substantial biological differences between the two studies (**Fig. 4B-C**, **Fig. 5A** of Suppl. Discussion 2). Lastly, we could not identify equivalents of the temporally enriched (9-11 pcw) PDE4C^{high}_EC population in the dataset of Farah *et al.*, which can potentially be explained by technical differences in the data processing, or inconsistent age determination between the two studies.

Integration of the Mesenchymal Cell Subsets

Since Farah *et al.* included both mural and non-mural mesenchymal cells, as well as epicardial cells in their subset labeled as mesenchymal compartment, we also integrated fine-grained clusters of non-mural mesenchymal cell and fibroblast subset with the coarse-grained clusters of pericytes (PC), smooth muscle cells of the outflow tract and great arteries (OFT_SMC) and coronary arteries (CA_SMC) in our dataset, as well as epicardial cells (EpC) for a comparative analysis of these subsets between the two datasets (**Fig. 6A-C** of Suppl. Discussion 2).

Corresponding major mesenchymal populations of the two datasets showed consistent enrichment of several important markers defined by the independent analysis of the dataset by Farah *et al.* However, we observed substantial differences on the level of fine-grained cell states in both datasets, highlighting a high level of molecular heterogeneity in this cellular subset (**Fig. 7A** of Suppl. Discussion 2). As previously discussed in the context of major cell population, Farah *et al.* discerned only one vascular smooth muscle population (VSMC) in their analysis, which they spatially traced to the walls of both the coronary arteries and the outflow tract region. We, on the other hand, identified distinct smooth muscle states with vastly different transcriptomics characteristics in our dataset, mapped to these two locations (CA_SMC and OFT_SMC, respectively). Even by applying label transfer using our cluster annotations as a reference, we only identified a minor population in the dataset of Farah *et al.* with a profile consistent with OFT_SMC, while the bulk of their VSMC population (consistent with the M1 cluster) received a CA_SMC label, supporting a better agreement with the transcriptomic characteristics of coronary smooth muscle cells (**Fig. 7B** of Suppl. Discussion 2). One possible reason for this discrepancy might be the underrepresentation of the region of the OFT and great arteries in the dissected tissue pieces used to generate the single-cell dataset. This is further supported by the similarly low number of cells receiving the Adv_FB_1 label, corresponding to adventitial fibroblasts within the same region (**Fig. 7B** of Suppl. Discussion 2). With this resolution, separate pericyte clusters could be observed also in the dataset of Farah *et al.*, which showed a consistent marker profile and interspersed occurrence on the UMAP with the corresponding PC population from our dataset. Similarly, the epicardial cell clusters appeared well-integrated between the two datasets (**Fig. 6B-C** and **7A-B** of Suppl. Discussion 2).

Among the remaining clusters, most of the expected major developmental cardiac mesenchymal cell populations, such as epicardium-derived progenitor cells (EPDC), valve mesenchymal cells, and interstitial fibroblasts of the cardiac chambers, were accounted for, with several clusters each in both datasets, although these fine-grained subpopulations were not consistent between the two studies (**Fig. 7A** of Suppl. Discussion 2). In our independent analysis, we showed that the transcriptomic heterogeneity represented by the fine-grained clusters of this subset was also reflected in distinct spatial patterns of the related cell states (such as the layered arrangement of interstitial fibroblast states in the ventricular wall (Int_FB_1-3); distinct EPDC states in the atrioventricular groove (EPDC_1) and the rest of the heart surface (EPDC_2); different cell states in the free region of the cardiac valve cusps/leaflets (VIC), their roots and the internal regions of the annulus fibrosus (Valve_MC_1-2), and the external parts of the latter structure (AnnFibr_FB); and a mesenchymal cell state with pericyte-like transcriptomic characteristics (Peric_MC^{fg}), enriched in the

atria and the inner ventricular wall; etc.), based on which we could construct a detailed map of their relative spatial organization (Fig. 6A-H, Ext. Fig. 6A-G), data also available in our interactive viewer). In their spatial analysis, Farah *et al.* discern the spatial distribution of six of the related non-mural mesenchymal cell populations (trabecular vFibro, compact vFibro, aFibro, EPDC, adFibro, VIC), providing a coarser depiction of their spatial relations (data available in the interactive viewer of Farah *et al.*). Importantly, we were able to corroborate the presence of the majority of our spatially annotated mesenchymal cell-fibroblast cell states in the dataset of Farah *et al.*, where smaller discrepancies (such as the lack of transferred Int_FB_3, EPDC_1, Prol_FB_1 and PDE4C^{high}_FB^{fg} labels) might be explained by the differences in data processing and the analyzed time frame (**Fig. 7B and 8A-B** of Suppl. Discussion 2).

In terms of their transcriptomic profiles, highly enriched markers of the vFibro population (consistent with the M10 cluster) (*GPC3*, *KLF2*, *DLK1*) of Farah *et al.* displayed the highest expression in the fibro-adipogenic progenitor-like (FAP) cell state identified in our dataset (**Fig. 7A** of Suppl. Discussion 2). This population showed a dynamic temporal expansion under the investigated timeframe in our study, making it plausible that this cell state has a dominant contribution to the ventricular fibroblast compartment in the, on average, later developmental phases analyzed by Farah *et al.* The aFibro population (consistent with the M11 and M12 clusters) of Farah *et al.* shared several important markers (*TNC*, *APOE*) with the Peric_MC^{fg} cluster in our dataset (**Fig. 7A** of Suppl. Discussion 2), which also appeared to be the dominant mesenchymal component of the atrial walls, besides an additional prominent localization in the inner ventricular wall (Fig. 6B). Notably, joint embedding of single-cell RNA-sequencing and MERFISH cell profiles by Farah *et al.* showed the largest co-occurrence of the trabecular vFibro MERFISH profile with the aFibro single-cell cluster, corroborating our mapping results (and thus the not exclusively atrial localization) of this cell state.

The most prominent discrepancy between the two datasets concerns a population identified as dorsal mesenchymal protrusion (DMP; consistent with M20 and M21 non-proliferating clusters) by Farah *et al.* (**Fig. 6B-C** and **7A-B** of Suppl. Discussion 2), based on them being derived from atrial samples at the earliest analyzed developmental phases, and their enrichment of *OSR1* and *TECRL*. While these genes were indeed described as being expressed by cells within the DMP in developing mouse hearts, they also occur in other cardiac cellular components including mesenchymal cells and therefore cannot be considered specific enough to allow for the identification of cells related to this distinct structure. To our knowledge, there are no reports available yet describing the complete transcriptomic profile of DMP cells, thus their identification based on the above-listed parameters is uncertain, especially without any confirmatory cell state mapping result. Interestingly, on the

integrated UMAP, the M20 cluster shows the closest association with the adventitial fibroblast population mapped to the walls of the great vessels in our dataset (Adv_FB_1), and the M21 cluster with the small CALN1^{high}_FB population, showing highest spatial overlap with the SAN_CM and AVN_CM pacemaker-conductive cardiomyocytes in the region of the sinoatrial and atrioventricular nodes (**Fig. 6B-C** of Suppl. Discussion 2). Adventitial fibroblast components of the great arteries and the developing coronary vasculature are not annotated in the single-cell dataset of Farah *et al.* (**Fig. 6C** of Suppl. Discussion 2). Among the proposed DMP markers of Farah *et al.*, *TECRL* was highly expressed in the CALN1^{high}_FB and EPDC_1 clusters, and beyond these populations, *OSR1* was also enriched in the EPDC_2, Adv_FB_1 and Adv_FB_2 clusters (Suppl. Table 6). Our label transfer strategy also supported the presumed connection between the M20 clusters of Farah *et al.* and adventitial fibroblast populations (Adv_FB_1 and Adv_FB_2) identified in our study, while the M21 cluster contributed to several different labels, including all cells in the relabeled CALN1^{high}_FB population (**Fig. 7B** of Suppl. Discussion 2). Although highly enriched markers of the relabeled Adv_FB_1 population of Farah *et al.* appeared only in a small percentage of the corresponding cluster of our dataset (**Fig. 8B** of Suppl. Discussion 2), this discrepancy might be explained by the difference in the age of the compared cells, since dramatic transcriptomic shifts occur within this region in the time frame covered by the two datasets, also supported by our analysis (Suppl. Fig. 7A-B). Based on these observations, it cannot be excluded that a substantial part of the mesenchymal cell population annotated as DMP by Farah *et al.* in fact represents adventitial components of the outflow tract and great arteries, which might have remained attached to the dissected atrial chambers in larger amounts in smaller hearts collected from earlier developmental points.

Integration of the Innervation-Related Cell Subsets

We observed a good integration between the innervation-related cell subset of our dataset with the neuronal compartment of Farah *et al.* (**Fig. 9A-C** of Suppl. Discussion 2). Importantly, we noticed a probable misannotation of major populations in this subset by Farah *et al.*, labeling two populations in their mid-grained clustering (Neural Crest and Neural Crest_Proliferating) as neural crest cells, which in fact cease to exist in their undifferentiated form in the human heart by 5 postconceptional weeks[68,69], substantially earlier than the first sampled developmental stage in their study. Accordingly, Farah *et al.* did not identify more differentiated neuroblasts or glial cells, which, according to our work, are not only present but have important regulatory functions already in the developing heart (Fig. 4H). By label transfer, we could confirm all these populations in the dataset of Farah *et al.* (discussed below), suggesting misannotation, rather than any major biological difference, as the source of disagreement between the cluster labels in the two studies.

The Schwann cell precursor (SCP) populations from the two datasets appeared interspersed in the integrated UMAP, but the five fine-grained SCP states in our dataset did not align with the four corresponding clusters (N3-N7) in the dataset of Farah *et al.* The small population of myelinating Schwann cells from our study appeared closely associated with the N5 cluster of Farah *et al.* Importantly, the N3 cluster also overlapped with the chromaffin cell population (Chrom_C), and the 'bridge state', representing transitional cells between the SCPs and chromaffin and neuronal cell states, identified in our study. The N1 and N2 clusters (consistent with the mid-grained populations mistakenly identified as neural crest cells) also appeared in consistent positions with the two autonomic neuroblast clusters identified by us but did not align one-by-one with each other (**Fig. 9B-C** of Suppl. Discussion 2).

To further investigate the relationship between the two datasets, we performed label transfer, using our fine-grained cluster annotation as a reference (**Fig. 10A-C** of Suppl. Discussion 2). The results of this analysis confirmed several of the observations discussed above, regarding the inconsistent cluster distribution within the SCP populations, and the presence of more differentiated, myelinating glial cells, transitioning "bridge" cells, and most importantly, a small population of cardiac chromaffin cells in the dataset of Farah *et al.* The cell populations identified by label transfer in this dataset featured a consistent enrichment profile of several key markers of the listed cell states, including differentiation markers and enzymes involved in neurotransmitter metabolism. These results supported our observation regarding the predominance of somatostatinergic (*SST*), and to a lesser extent, cholinergic (*CHAT*) transmission from the developing autonomic neurons in the investigated timeframe, and confirmed the distinct molecular signature of resident chromaffin cells, including differentiation markers (*CHGB*, *CHGA*, *PENK*, *DLK1*), local hypoxia sensors (*EPAS1*, *COX4I2*, *HIGD1C*, *NDUFA4L2*), and enzymes involved in catecholamine synthesis (*TH*, *DBH*, *PNMT*) (**Fig. 10B** of Suppl. Discussion 2). These results provide further support to our proposed model of regulatory mechanisms between the developing autonomic nervous system, resident cardiac chromaffin cells, and the developing pacemaker-conduction system.

Comparison of Cardiac Niches and Cellular Communities

In the study of Farah *et al.*, they identified 75 single-cell and 27 MERFISH-based cell populations. By co-segregating the spatially validated cell states within individual cell zones (150 µm radius), they described 13 cellular communities (CCs) which allowed for the targeted assessment of CCs with varying complexity and purity with a single-cell resolution. However, since the spatial mapping of cell states was performed using a targeted spatial transcriptomics approach (238 transcripts), all

downstream molecular analysis considering the spatial component depends on the imputation of complete gene expression profiles and cell labels from the complementary single-cell dataset.

In our study, we determined spatial relations between cardiac cell states based on their predicted localization, obtained through the deconvolution of the transcriptome-wide Visium dataset with the complete molecular profiles of 72 identified fine-grained single-cell states. This unbiased approach provides spatial coordinates for all clusters included in the analysis by considering their entire transcriptome, thus the spatially reflected heterogeneity and overall probability of cell type identification is not limited by a targeted gene panel. However, the spatial resolution of cell state mapping remains at the level of the single Visium spot (~55 μm in diameter, representing 10-20 individual cells per spot), but the precise cell composition can be deduced from the predicted proportions for each analyzed cell state. Considering the high granularity of our dataset in terms of spatially distinct cell states, we opted to delineate cardiac compartments and niches based on their dominant cellular components, instead of their level of compositional complexity, as investigated by Farah *et al.* Accordingly, we calculated pairwise correlation-based co-detections scores between all fine-grained cell states to assess their spatial co-occurrence in the entire dataset, and used a graph-based visualization strategy to outline the dominant cellular components of structural and functional units within the developing heart tissue (Fig. 7A). By using this framework for downstream investigations, such as cell-cell communication analysis, we focused on the most distinguishing cellular, and thus molecular, components within these units, which appeared highly heterogeneous due to the high number of spatially characterized cell states in our dataset. The graph-based niche identification strategy assigns each cell state to their most prominent cardiac niche or structure, without considering potential secondary contributions to other niches or the presence of less abundant cell states within spots that may still play meaningful roles in ligand-receptor interactions in the analyzed compartments.

Despite the conceptual differences between the two approaches, main observations regarding major cellular components and level of complexity of different CCs defined by Farah *et al.* broadly align with our analysis, as illustrated by the arrangement of the co-detection-based niche graph representing the level of spatial association between the analyzed cell states. This includes higher cellular complexity of ventricular chambers in comparison to the atria, and decreasing cellular complexity from the outside to the lumen of the ventricles (including the enrichment of fibroblasts and cells of the coronary vasculature in the compact layer of the myocardium), as well as dominant cellular components of the chamber- and non-chamber-related CCs, although, as described above, Farah *et al.* provide lower granularity in terms of spatially confirmed cell states in their spatial analysis. For

instance, we annotate and spatially map two consecutively organized cardiomyocyte populations (AVB-BB_CM and PF_CM) as part of the ventricular conduction system niche, while Farah *et al.* define a common MERFISH profile (vCM-His-Purkinje) for these two cell types in their high-level analysis. Importantly, in the IFT/SAN CC, Farah *et al.* validate our cell state mapping results regarding the close spatial association between neuronal and pacemaker cells in the sinoatrial node region, but do not identify any specific fibroblast (similar to CALN1^{high}_FB) or endothelial (similar to Ven_EC) cell states associated with this functional unit. Farah *et al.* also describe expected components of the regions of the great arteries as part of an OFT CC, but unlike in our study, they do not show an enrichment of cells with neuronal profiles in these regions, likely due to the low representation of these segments in the analyzed sections. Related to the atrioventricular node, we found the resident AVN_CM conductive cardiomyocytes show the strongest spatial association with the previously mentioned CALN1^{high}_FB population of the identified mesenchymal cell states, instead of the predominant atrial mesenchymal population (identified as the aFibro population in the dataset of Farah *et al.*, and Peric_MC^{fg} in our study). As suggested by the results of our label transfer approach, a small mesenchymal population consistent with the CALN1^{high}_FB cluster can also be identified in the single-cell dataset of Farah *et al.*, thus their similar spatial associations with functional units of the cardiac pacemaker-conduction system are also possible but cannot be assessed based on the presented MERFISH gene panel. The subepicardial CC identified by Farah *et al.* (which only included the area of the atrioventricular groove, but not the rest of the heart surface) is characterized by several cellular components (i.e. EPDCs, endothelial and mesenchymal cells of coronary and lymphatic vascular structures, and leukocytes) that also showed spatial enrichment in the corresponding regions in our dataset, however, our analysis highlighted additional fine-grained cell states with characteristic localization in this region, such as fibroblasts of the external regions of the annulus fibrosus (AnnFibr_FB) and a subset of the fibro-adipogenic progenitor-like cells (FAP). While the two studies are also in agreement regarding the major cellular components of the developing cardiac valves, including valve endothelial cells (VECs), valve interstitial cells (VICs) and cardiomyocytes, we provided additional layers of the spatial arrangements of these cell states by identifying side-specific subpopulations of VECs, and three spatially distinct valve-related mesenchymal cell states. At the same time, we observed only one distinct cardiomyocyte cluster with strong spatial enrichment in the valve area (vCM_1), as opposed to the two, side-specific MERFISH populations proposed by Farah *et al.* (vCM-LV/RV-AV).

The spatial distribution of several cellular communities identified by Farah *et al.*, especially the ones representing larger myocardial or vessel compartments, showed a high level of similarity to

the molecular regions identified by a spatially informed clustering approach (BANKSY) utilized in our study (Suppl. Fig. 1A-B), confirming the close association with regional molecular patterns and spatially separated cellular communities.

Further refinements of the ventricular MERFISH states of Farah *et al.* identified additional subclusters of cardiomyocytes and fibroblasts, displaying a layered appearance within the ventricular walls, and providing a higher granularity in the identification of ventricular cellular communities. Joint embedding between the newly defined MERFISH profiles and the single-cell dataset highlighted some discrepancies, such as most of the vCM-RV-compact and vCM-RV-hybrid MERFISH population showing the highest co-occurrence with the vCM_trabecular single-cell cluster, and most of the trabecular vFibro MERFISH population with the aFibro single-cell cluster, as discussed above. In summary, while many findings are consistent between the two studies, we observe key discrepancies arising from the use of different spatial technologies – transcriptome-wide spatial data, such as that provided by Visium analysis, compared to targeted approaches like MERFISH – for determining spatial coordinates of cellular populations.

PLXN-SEMA Expression Related to Ventricular Development

In their study, Farah *et al.* proposed a central role for multicellular SEMA-PLXN interactions in the process of ventricular compaction, and present *in vitro* (iPS-derived cardiomyocyte culture) and *in vivo* (conditional SEMA3C knockout mouse) support for their model, where SEMA3C+ compact vFibro cells may attract PLXNA2+PLXNA4+ trabecular and hybrid vCMs to the intermediate-LV and outer-LV CC layers, whereas SEMA6A+SEMA6B+ BECs may prevent these vCMs from migrating by repelling them after contact. In our Visium dataset, *SEMA3C* and *PLXNA4* appeared at consistent positions in the heart tissue as reported by Farah *et al.*, along with the spatial enrichment of *SEMA6A* and *SEMA6B* in the compact myocardial layer. Our single-cell dataset corroborated the enrichment of these genes in the relevant cellular subsets, although *PLXNA4* and *PLXNA2* also appeared to be strongly enriched in the endocardial endothelial cell population, besides subsets of ventricular cardiomyocytes. These molecular arrangements support the possibility of the SEMA-PLXN interactions proposed by Farah *et al.*

SUPPLEMENTARY DISCUSSION 2 – FIGURE LEGENDS

Supplementary Discussion 2 - Figure 1. Integration of Major Cell Populations. A. UMAP of the integrated datasets, embedded by the original dataset. **B.** Integrated UMAP highlighting the major cell types (merged coarse-grained clusters) of Lázár *et al.* **C.** Integrated UMAP highlighting the major cell classes of Farah *et al.*

Supplementary Discussion 2 - Figure 2. Integration of Cardiomyocyte Subsets. A. UMAP of the integrated cardiomyocyte subsets, embedded by the original dataset. **B.** Integrated UMAP highlighting major cardiomyocyte populations (merged fine-grained clusters) of Lázár *et al.* **C.** Integrated UMAP highlighting cardiomyocyte populations (left) and clusters (right) of Farah *et al.*

Supplementary Discussion 2 - Figure 3. Agreement between CPCS and Valve-Related Cardiomyocyte States. A. Marker enrichment (defined based on clusters of Lázár *et al.*) in CPCS and valve-related cardiomyocyte clusters of Lázár *et al.* (left) and Farah *et al.* (right). **B.** Distribution of cells by original cluster in the relabeled CPCS clusters of Farah *et al.* Avg. exp.–average expression, Pct. exp.–percent of expressing cells.

Supplementary Discussion 2 - Figure 4. Integration of Endothelial Cell Subsets. A. UMAP of the integrated endothelial cell subsets, embedded by the original dataset. **B.** Integrated UMAP highlighting fine-grained endothelial cell clusters of Lázár *et al.* **C.** Integrated UMAP highlighting endothelial cell populations (left) and clusters (right) of Farah *et al.*

Supplementary Discussion 2 - Figure 5. Comparison of Transcriptomic Profiles of Endothelial Cell Clusters. A. Distribution of highly enriched markers of endothelial cell populations of Farah *et al.*, across fine-grained endothelial cell clusters of Lázár *et al.* (left), and endothelial cell clusters of Farah *et al.* (right). **B.** Distribution of cells by original cluster in the relabeled endocardial cushion-related, venous and venular endothelial cell clusters of Farah et al. **C.** Marker enrichment (defined based on the relabeled clusters of Farah *et al.*) in the endocardial cushion-related, venous and venular endothelial cell clusters in Lázár *et al.* (left) and Farah *et al.* (right). In A and C panels: Avg. exp.–average expression, Pct. exp.–percent of expressing cells.

Supplementary Discussion 2 - Figure 6. Integration of Mesenchymal Cell Subsets. A. UMAP of the integrated mesenchymal cell subsets, embedded by the original dataset. **B.** Integrated UMAP highlighting fine-grained mesenchymal cell-fibroblast and coarse-grained pericyte, smooth muscle cell and epicardial cell clusters of Lázár *et al.* **C.** Integrated UMAP highlighting mesenchymal cell populations (left) and clusters (right) of Farah *et al.*

Supplementary Discussion 2 - Figure 7. Comparison of Transcriptomic Profiles of Mesenchymal Cell Clusters. A. Distribution of highly enriched markers of mesenchymal cell populations of Farah *et al.*, across fine-grained mesenchymal cell-fibroblast and coarse-grained pericyte, smooth muscle cell and epicardial cell clusters of Lázár *et al.* (left), and mesenchymal cell clusters of Farah *et al.* (right). Avg. exp.–average expression, Pct. exp.–percent of expressing cells. **B.** Distribution of cells by original cluster in the relabeled mesenchymal cell-fibroblast, mural cell and epicardial cell clusters of Farah *et al.* (with 0.45 as prediction threshold).

Supplementary Discussion 2 - Figure 8. Label Transfer of Spatially Annotated Mesenchymal Cell-Fibroblast, Mural Cell and Epicardial Cell Clusters. **A.** Distribution of the reference (Lázár *et al.*, left) and relabeled (Farah *et al.*, right) mesenchymal cell-fibroblast, mural cell and epicardial cell clusters on the integrated UMAP. **B.** Marker enrichment (defined based on the relabeled clusters of Farah *et al.*) in the reference (Lázár *et al.*, left) and relabeled (Farah *et al.*, right) mesenchymal cell-fibroblast, mural cell and epicardial cell clusters (with 0.45 as prediction threshold). Avg. exp.–average expression, Pct. exp.–percent of expressing cells.

Supplementary Discussion 2 - Figure 9. Integration of Innervation-Related Cell Subsets. A. UMAP of the integrated innervation-related cell subset, embedded by the origin dataset. **B.** Integrated UMAP highlighting fine-grained innervation-related clusters of Lázár *et al.* **C.** Integrated UMAP highlighting neuronal populations (left) and clusters (right) of Farah *et al.*

Supplementary Discussion 2 - Figure 10. Label Transfer of Fine-Grained Innervation-Related Clusters. A. Integrated UMAP highlighting the relabeled innervation-related cell populations of Farah *et al.* **B.** Distribution of differentiation markers, hypoxia sensors and neurotransmitter markers across the reference (Lázár *et al.*) and relabeled (Farah *et al.*) innervation-related cell states. Avg. exp.–average expression, Pct. exp.–percent of expressing cells. **C.** Distribution of cells by original cluster in the relabeled innervation-related cell clusters of Farah *et al.*

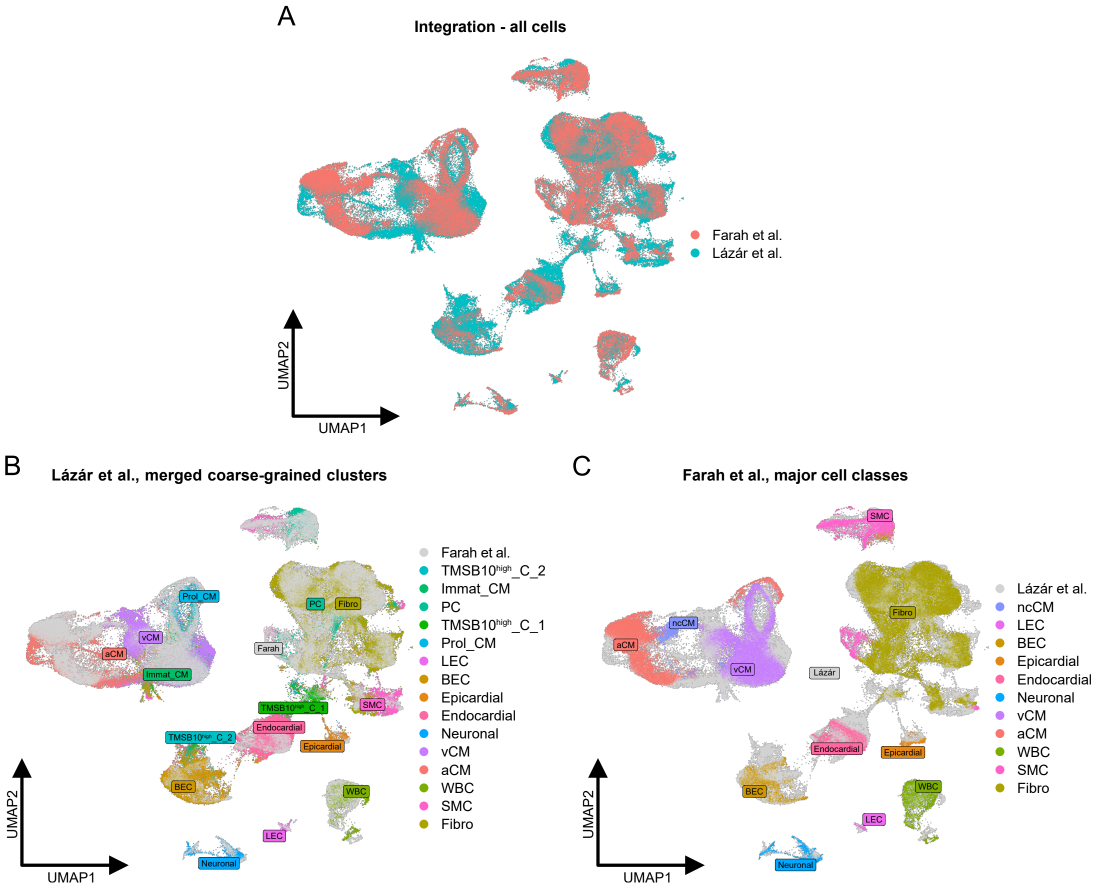

Supplementary Discussion 2 - Figure 1

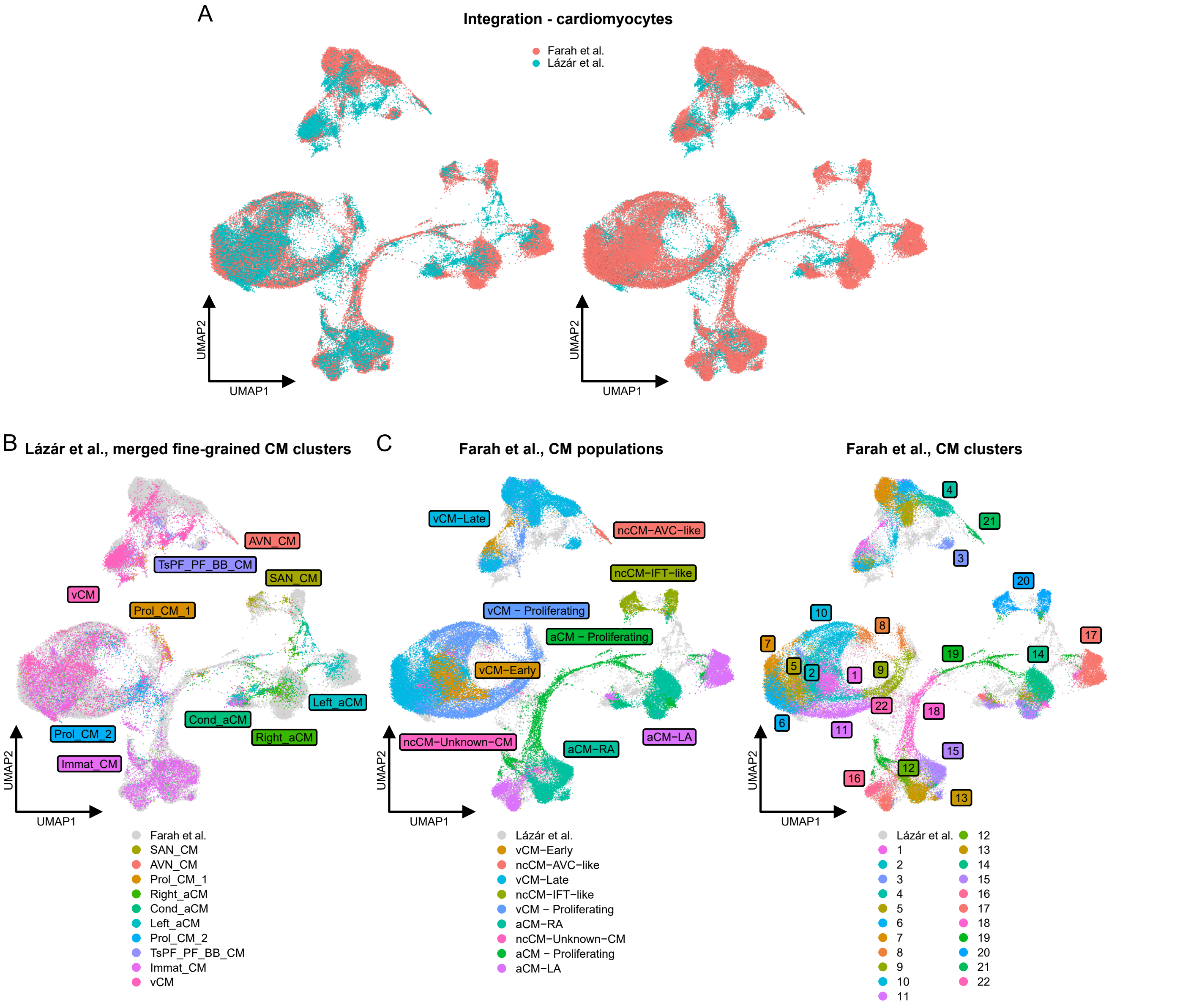

Supplementary Discussion 2 - Figure 2

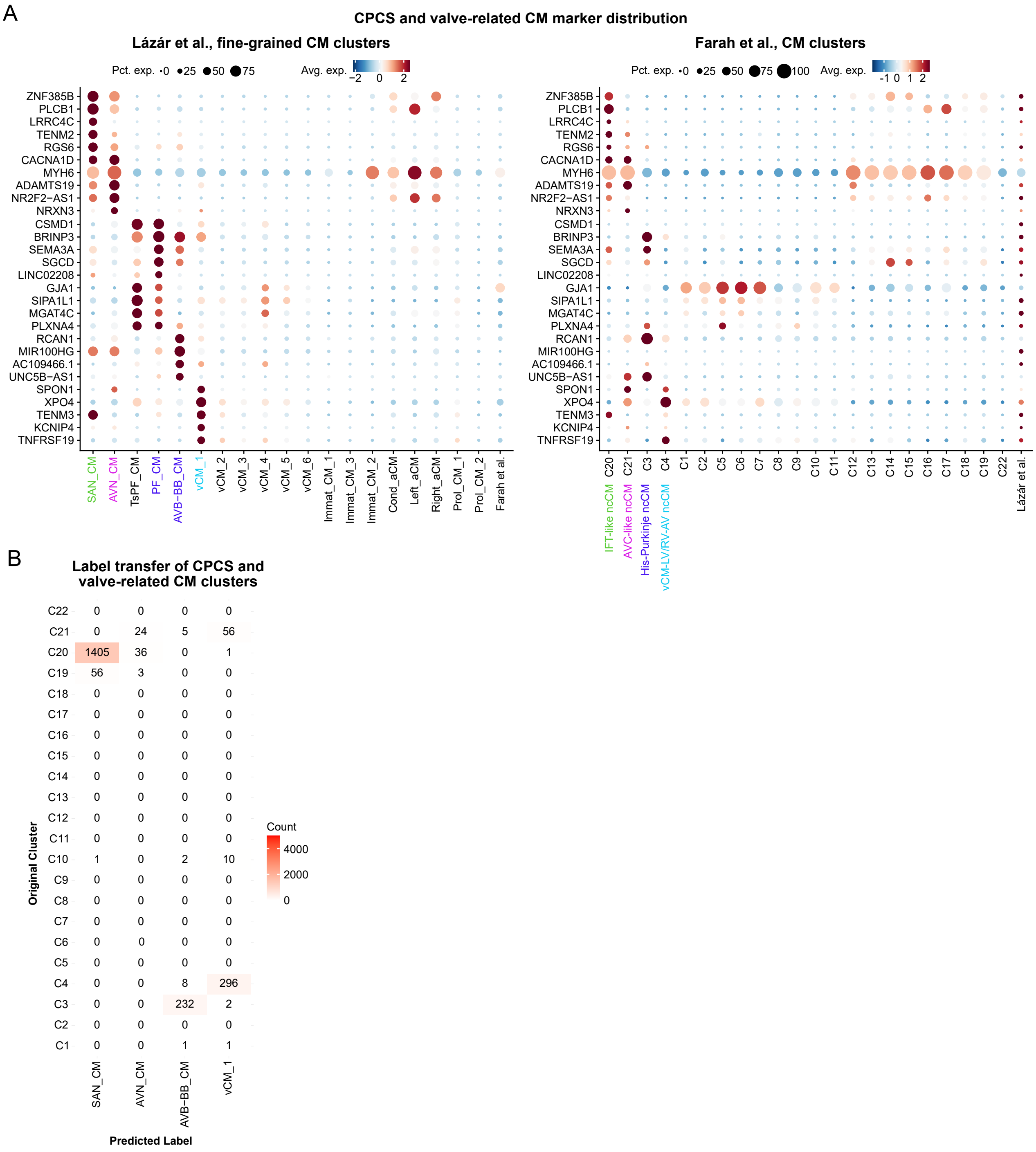
A
CPCS and valve-related CM marker distribution
Lázár et al., fine-grained CM clusters
Farah et al., CM clusters
Pct. exp.
Avg. exp.
ZNF385B
PLCB1
LRRC4C
TENM2
RGS6
CACNA1D
MYH6
ADAMTS19
NR2F2-AS1
NRXN3
CSMD1
BRINP3
SEMA3A
SGCD
LINC02208
GJA1
SIPA1L1
MGAT4C
PLXNA4
RCAN1
MIR100HG
AC109466.1
UNC5B-AS1
SPON1
XPO4
TENM3
KCNIP4
TNFRSF19
SAN_CM
AVN_CM
TsPF_CM
PF_CM
AVB-BB_CM
vCM_1
vCM_2
vCM_3
vCM_4
vCM_5
vCM_6
Immat_CM_1
Immat_CM_3
Immat_CM_2
Cond_aCM
Left_aCM
Right_aCM
Prol_CM_1
Prol_CM_2
Farah et al.
Lázár et al.
IFT-like ncCM
AVC-like ncCM
His-Purkinje ncCM
vCM-LV/RV-AV ncCM
B
Label transfer of CPCS and valve-related CM clusters
Original Cluster
Predicted Label
Count
4000
2000
0

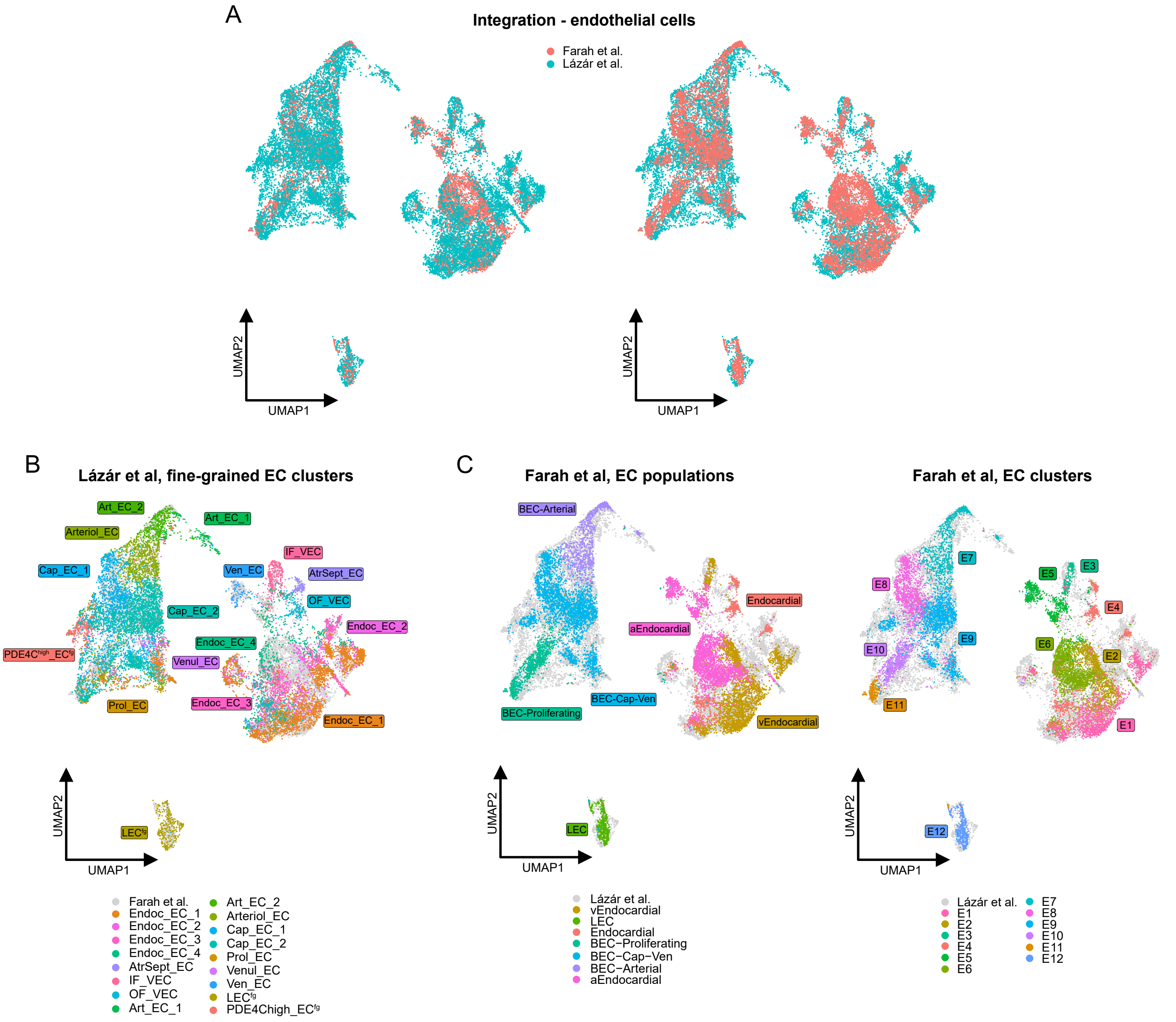

Supplementary Discussion 2 - Figure 4

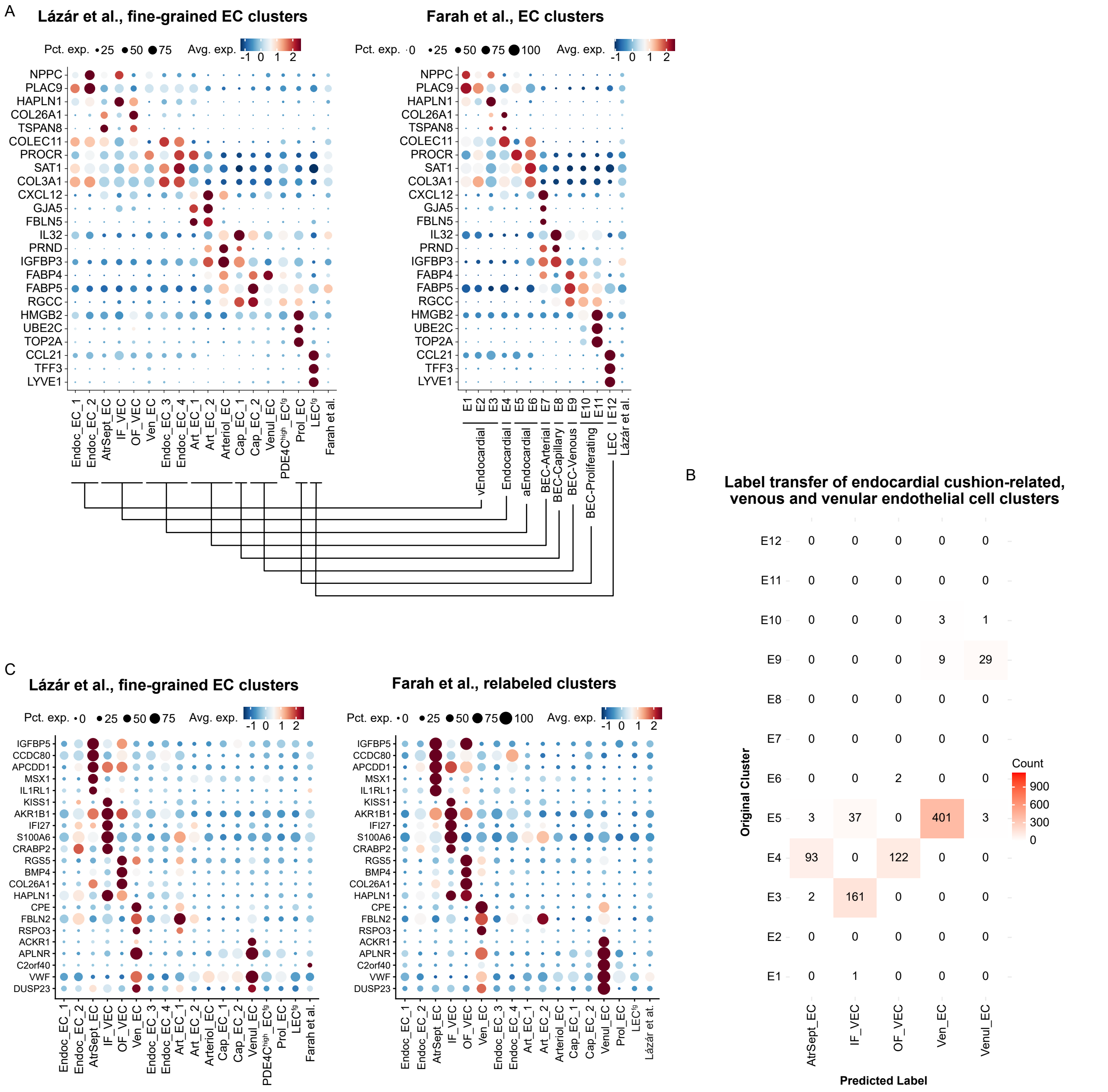

Supplementary Discussion 2 - Figure 5

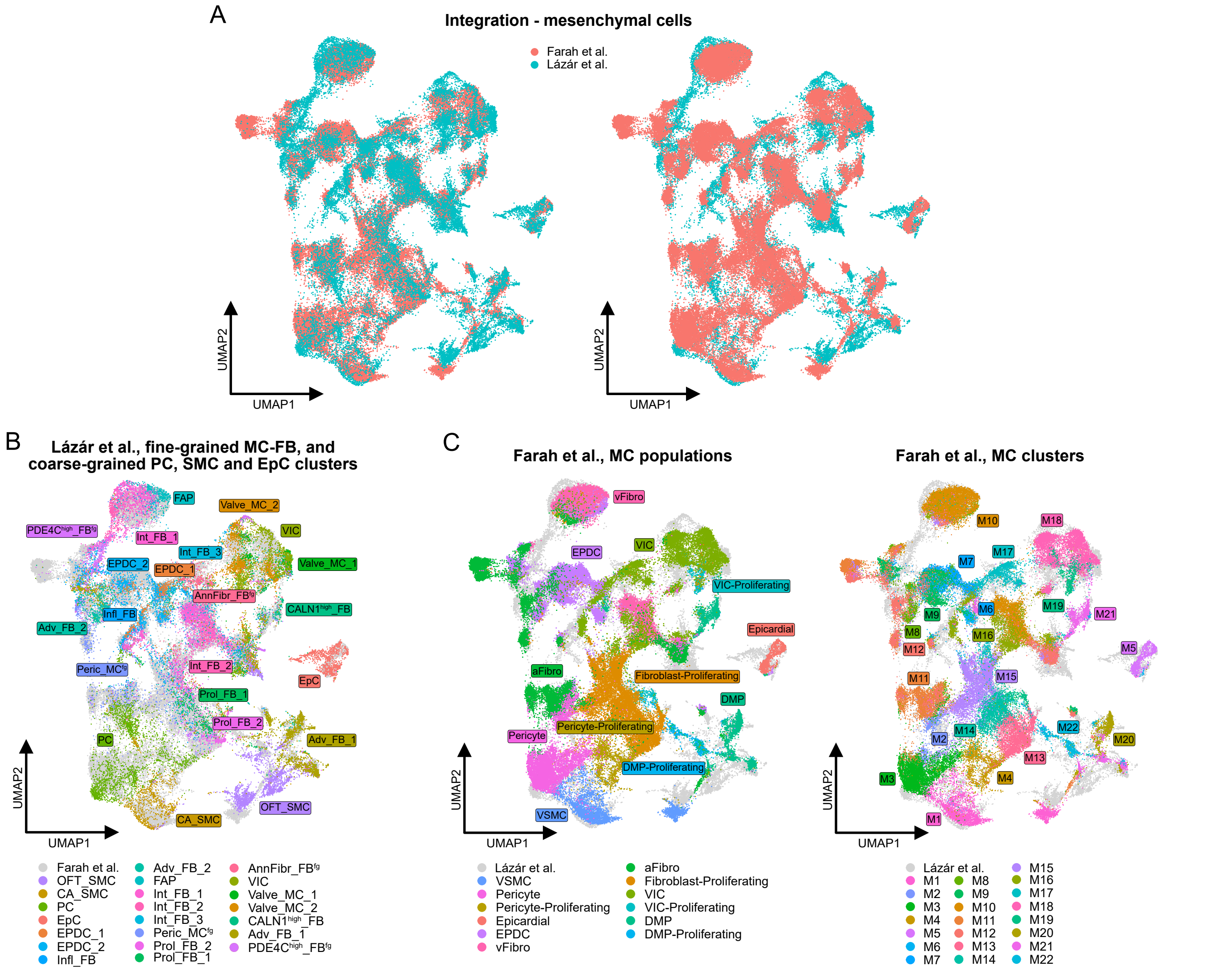

Supplementary Discussion 2 - Figure 6

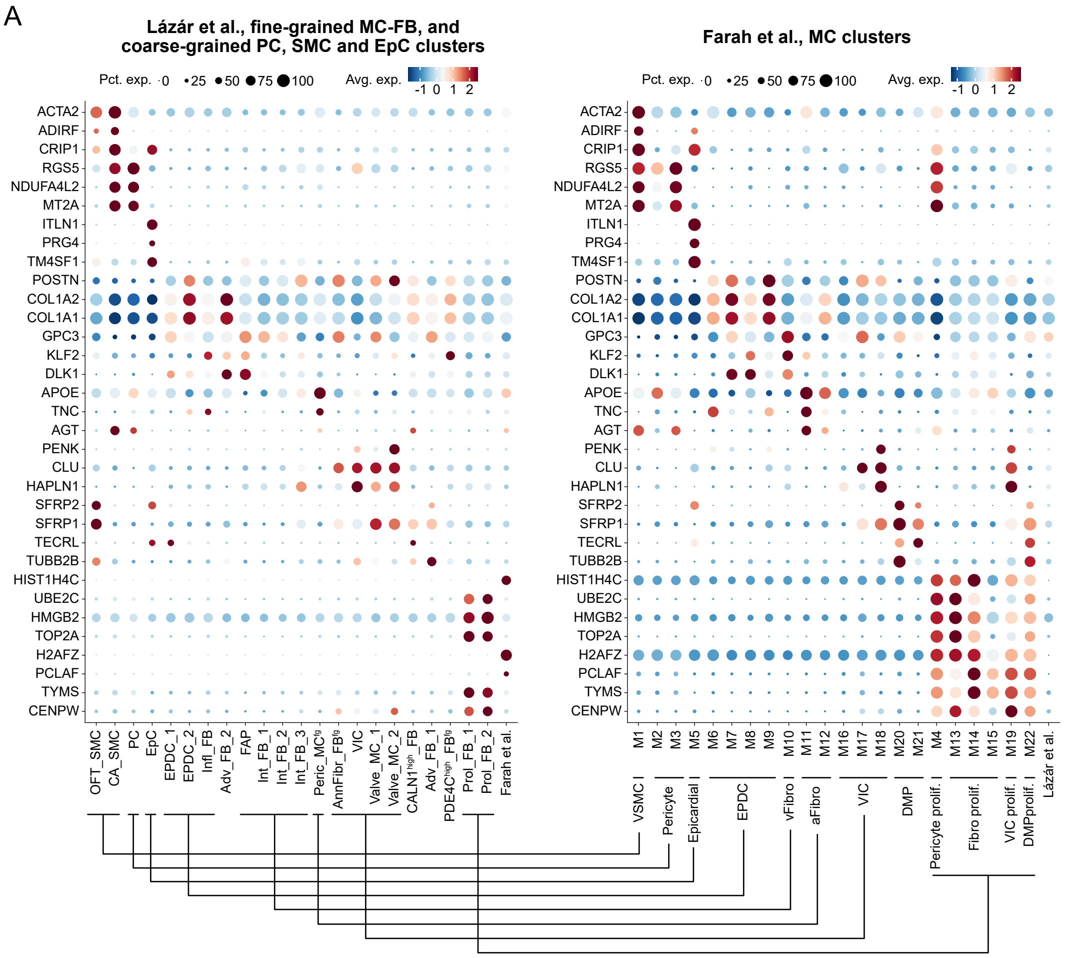

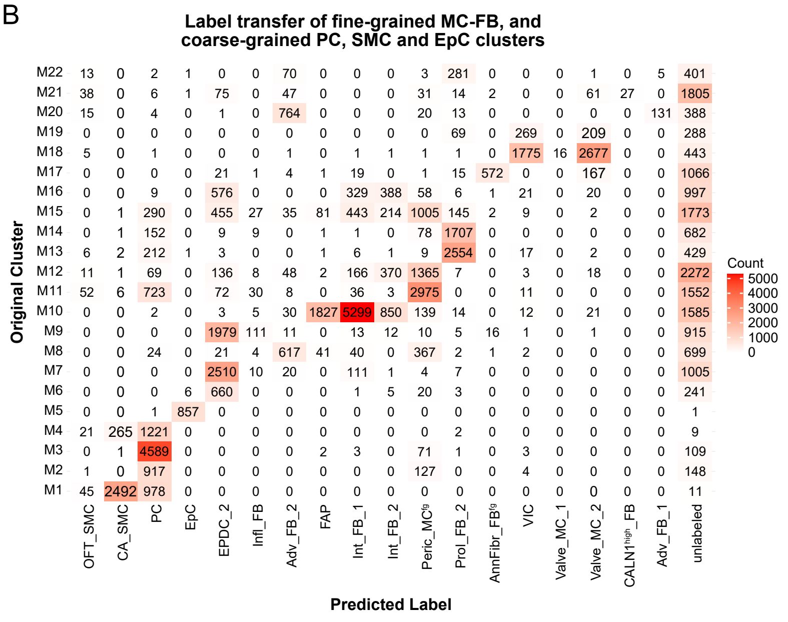

Supplementary Discussion 2 - Figure 7

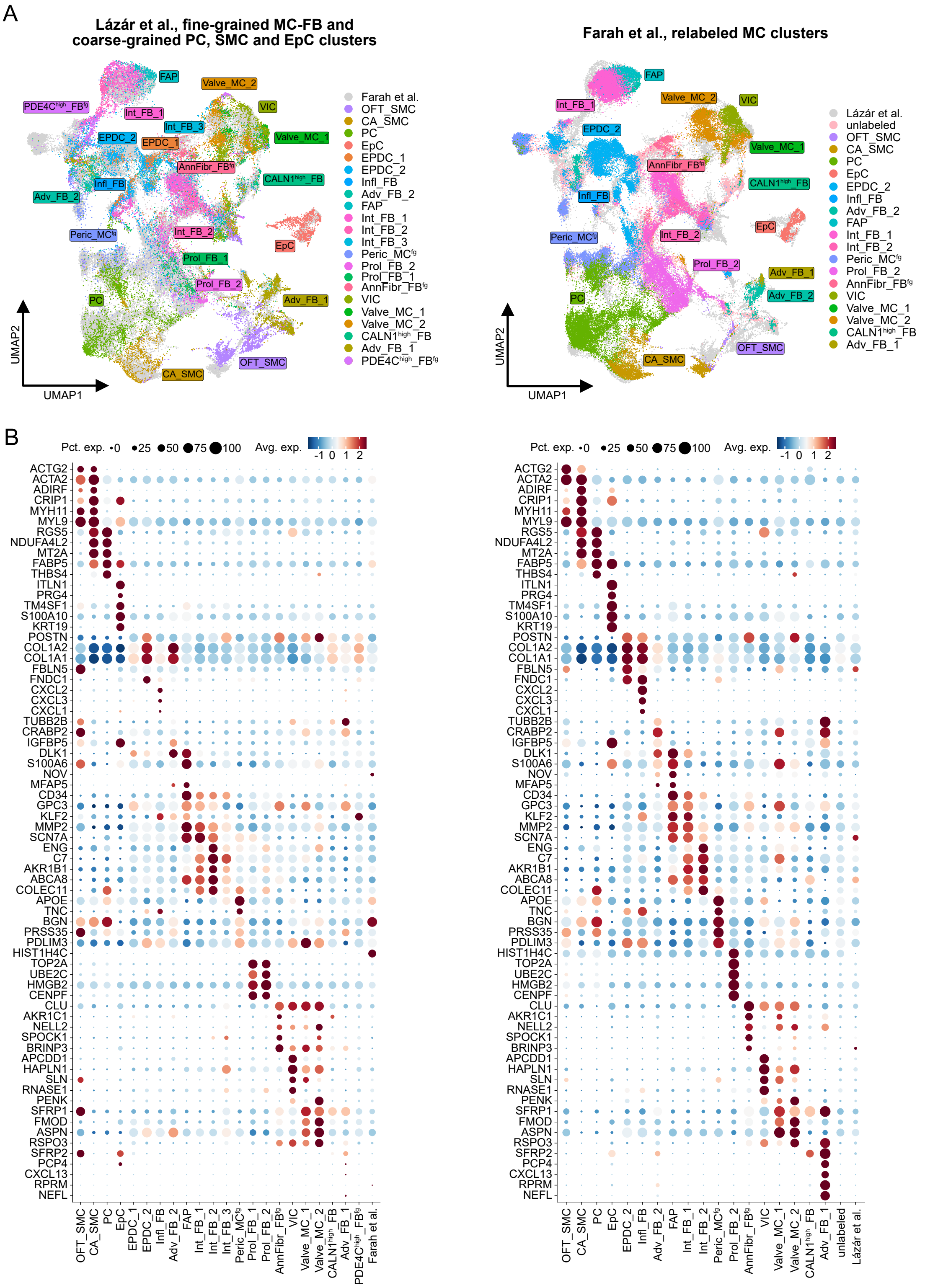

Supplementary Discussion 2 - Figure 8

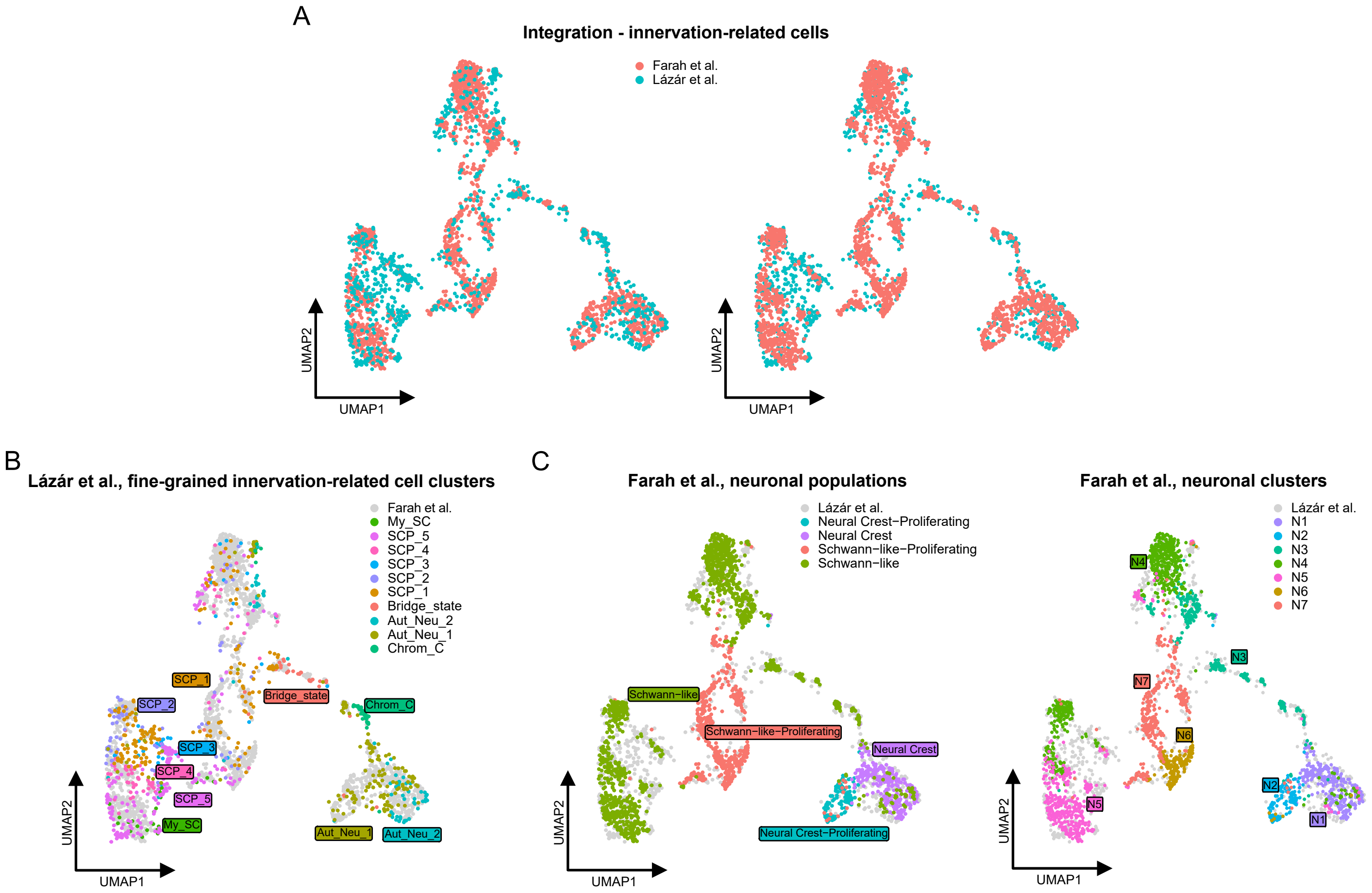

Supplementary Discussion 2 - Figure 9

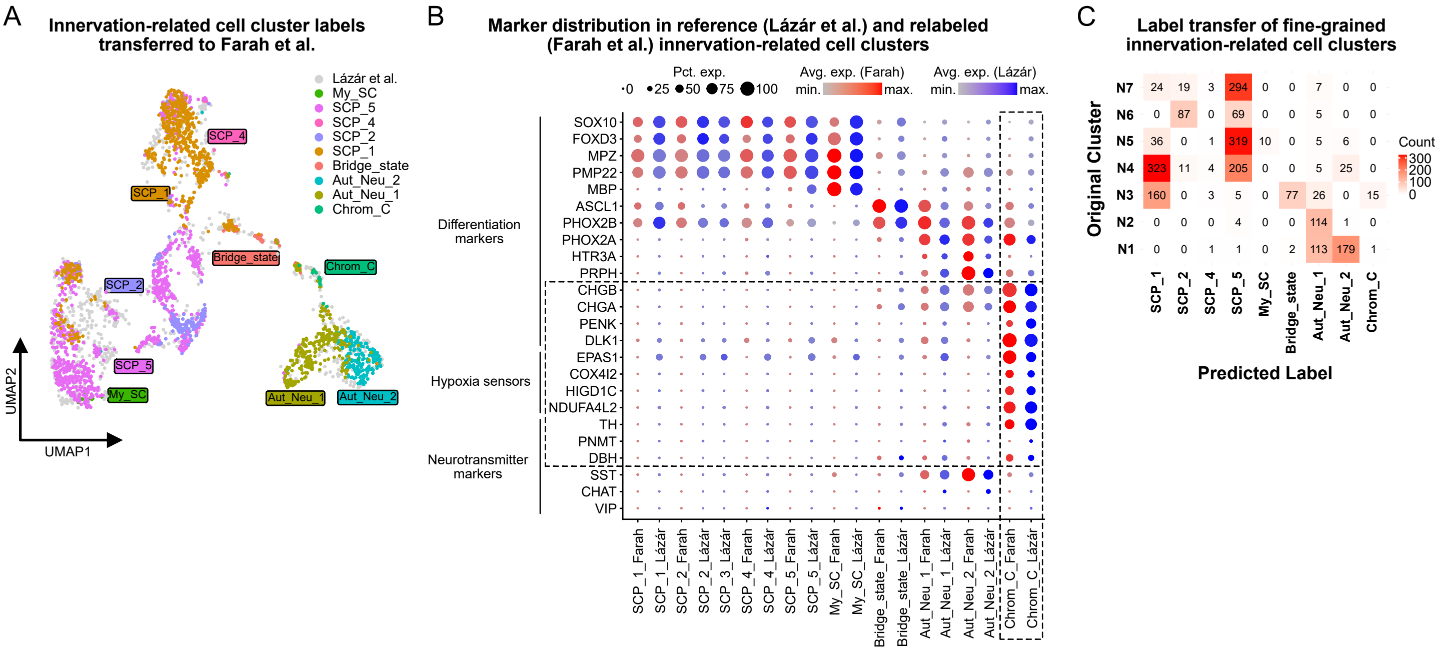

Supplementary Discussion 2 - Figure 10

SUPPLEMENTARY REFERENCES

1. R Core Team. R: A Language and Environment for Statistical Computing. R Foundation for Statistical Computing. https://www.r-project.org (2021).
2. Bergenstråhle, J., Larsson, L. & Lundeberg, J. Seamless integration of image and molecular analysis for spatial transcriptomics workflows. *BMC Genomics* **21**, 482 (2020).
3. Hao, Y. *et al.* Integrated analysis of multimodal single-cell data. *Cell* **184**, 3573–3587.e29 (2021).
4. Korsunsky, I. *et al.* Fast, sensitive and accurate integration of single-cell data with Harmony. *Nat. Methods* **16**, 1289–1296 (2019).
5. Singhal, V. *et al.* BANKSY unifies cell typing and tissue domain segmentation for scalable spatial omics data analysis. *Nat. Genet.* **56**, 431–441 (2024).
6. DeBruine, Z. J., Andrew Pospisilik, J. & Triche, T. J. Fast and interpretable non-negative matrix factorization for atlas-scale single cell data. *bioRxiv* 2021.09.01.458620 (2024).
7. R Core Team. R: A Language and Environment for Statistical Computing. R Foundation for Statistical Computing. https://www.r-project.org (2023).
8. McGinnis, C. S., Murrow, L. M. & Gartner, Z. J. DoubletFinder: Doublet Detection in Single-Cell RNA Sequencing Data Using Artificial Nearest Neighbors. *Cell Syst* **8**, 329–337.e4 (2019).
9. Zappia, L. & Oshlack, A. Clustering trees: a visualization for evaluating clusterings at multiple resolutions. *Gigascience* **7**, giy083 (2018).
10. Andersson, A. *et al.* Single-cell and spatial transcriptomics enables probabilistic inference of cell type topography. *Commun Biol* **3**, 565 (2020).
11. Larsson, L., Franzén, L., Ståhl, P. L. & Lundeberg, J. Semla: a versatile toolkit for spatially resolved transcriptomics analysis and visualization. *Bioinformatics* **39**, btad626 (2023).
12. Wickham, H. *ggplot2: Elegant Graphics for Data Analysis*. Springer International Publishing (2016).
13. Csardi, G. & Nepusz, T. The igraph software package for complex network research. *InterJournal*, **Complex Systems**, 1695. https://igraph.org (2006).
14. Ward, J. M. colorjam: Jam Color manipulation functions. http://github.com/jmw86069/colorjam (2024).
15. Efremova, M., Vento-Tormo, M., Teichmann, S. A. & Vento-Tormo, R. CellPhoneDB: inferring cell-cell communication from combined expression of multi-subunit ligand-receptor complexes. *Nat. Protoc.* **15**, 1484–1506 (2020).
16. Dimitrov, D. *et al.* Comparison of methods and resources for cell-cell communication inference

from single-cell RNA-Seq data. *Nat. Commun.* **13**, 3224 (2022).

17. Kanemaru, K. *et al.* Spatially resolved multiomics of human cardiac niches. *Nature* **619**, 801–810 (2023).
18. Bergen, V., Lange, M., Peidli, S., Wolf, F. A. & Theis, F. J. Generalizing RNA velocity to transient cell states through dynamical modeling. *Nat. Biotechnol.* **38**, 1408–1414 (2020).
19. Wolf, F. A., Angerer, P. & Theis, F. J. SCANPY: large-scale single-cell gene expression data analysis. *Genome Biol.* **19**, 15 (2018).
20. Faure, L., Soldatov, R., Kharchenko, P. V. & Adameyko, I. scFates: a scalable python package for advanced pseudotime and bifurcation analysis from single-cell data. *Bioinformatics* **39**, btac746 (2023).
21. Aibar, S. *et al.* SCENIC: single-cell regulatory network inference and clustering. *Nat. Methods* **14**, 1083–1086 (2017).
22. Huynh-Thu, V. A., Irrthum, A., Wehenkel, L. & Geurts, P. Inferring regulatory networks from expression data using tree-based methods. *PLoS One* **5**, e12776 (2010).
23. Chen, W. *et al.* Single-cell transcriptomic landscape of cardiac neural crest cell derivatives during development. *EMBO Rep.* **22**, e52389 (2021).
24. Zhang, S.-S. *et al.* Iroquois homeobox gene 3 establishes fast conduction in the cardiac His-Purkinje network. *Proc. Natl. Acad. Sci. U. S. A.* **108**, 13576–13581 (2011).
25. Seropian, I. M., Cassaglia, P., Miksztowicz, V. & González, G. E. Unraveling the role of galectin-3 in cardiac pathology and physiology. *Front. Physiol.* **14**, 1304735 (2023).
26. Spielmann, N. *et al.* Extensive identification of genes involved in congenital and structural heart disorders and cardiomyopathy. *Nature Cardiovascular Research* **1**, 157–173 (2022).
27. Mizutani, H. *et al.* Overexpression of myosin phosphatase reduces Ca(2+) sensitivity of contraction and impairs cardiac function. *Circ. J.* **74**, 120–128 (2010).
28. Nicin, L. *et al.* A human cell atlas of the pressure-induced hypertrophic heart. *Nature Cardiovascular Research* **1**, 174–185 (2022).
29. Zhao, Y. *et al.* Hypoxia-induced signaling in the cardiovascular system: pathogenesis and therapeutic targets. *Signal Transduct Target Ther* **8**, 431 (2023).
30. Mill, P., Christensen, S. T. & Pedersen, L. B. Primary cilia as dynamic and diverse signalling hubs in development and disease. *Nat. Rev. Genet.* **24**, 421–441 (2023).
31. Hansen, J. N. *et al.* A cAMP signalosome in primary cilia drives gene expression and kidney cyst formation. *EMBO Rep.* **23**, e54315 (2022).
32. Stankunas, K. *et al.* Pbx/Meis deficiencies demonstrate multigenetic origins of congenital heart disease. *Circ. Res.* **103**, 702–709 (2008).

33. Zou, M. *et al.* Prdm6 drives ductus arteriosus closure by promoting ductus arteriosus smooth muscle cell identity and contractility. *JCI Insight* **8**, e163454 (2023).
34. Hong, L. *et al.* Prdm6 controls heart development by regulating neural crest cell differentiation and migration. *JCI Insight* **7**, e156046 (2022).
35. Lin, J.-H. I. *et al.* Mutation of LRP1 in cardiac neural crest cells causes congenital heart defects by perturbing outflow lengthening. *Commun Biol* **3**, 312 (2020).
36. Moreno-Domínguez, A., Colinas, O., Smani, T., Ureña, J. & López-Barneo, J. Acute oxygen sensing by vascular smooth muscle cells. *Front. Physiol.* **14**, 1142354 (2023).
37. Volz, K. S. *et al.* Pericytes are progenitors for coronary artery smooth muscle. *Elife* **4**, e10036 (2015).
38. Bayraktar, S. *et al.* High-resolution atlas of the developing human heart and the great vessels. *bioRxiv* 2024.04.27.591127 (2024).
39. Farah, E. N. *et al.* Spatially organized cellular communities form the developing human heart. *Nature* **627,** 854–864 (2024).
40. Weber, K. T., Sun, Y., Bhattacharya, S. K., Ahokas, R. A. & Gerling, I. C. Myofibroblast-mediated mechanisms of pathological remodelling of the heart. *Nat. Rev. Cardiol.* **10**, 15–26 (2013).
41. Palis, J., Tober, J., Vemishetti, R., Koniski, A. & Waugh, R. The Megakaryocyte Lineage Arises in the Yolk Sac and Generates an Initial Wave of Large Embryonic Platelets in the Early Mammalian Embryo. *Blood* **104**, 566–566 (2004).
42. Gula, G. & Ratajska, A. Novel insights into embryonic cardiac macrophages. *Dev. Biol.* **488**, 1–10 (2022).
43. Bajpai, G. *et al.* The human heart contains distinct macrophage subsets with divergent origins and functions. *Nat. Med.* **24**, 1234–1245 (2018).
44. Stevens, S. M., von Gise, A., VanDusen, N., Zhou, B. & Pu, W. T. Epicardium is required for cardiac seeding by yolk sac macrophages, precursors of resident macrophages of the adult heart. *Dev. Biol.* **413**, 153–159 (2016).
45. Cahill, T. J. *et al.* Tissue-resident macrophages regulate lymphatic vessel growth and patterning in the developing heart. *Development* **148**, dev.194563 (2021).
46. Leid, J. *et al.* Primitive Embryonic Macrophages are Required for Coronary Development and Maturation. *Circ. Res.* **118**, 1498–1511 (2016).
47. Chen, Q. *et al.* Endothelial cells are progenitors of cardiac pericytes and vascular smooth muscle cells. *Nat. Commun.* **7**, 12422 (2016).
48. Asp, M. *et al.* A Spatiotemporal Organ-Wide Gene Expression and Cell Atlas of the Developing

Human Heart. *Cell* **179**, 1647–1660.e19 (2019).

49. Wei, S., Zhang, M., Zheng, Y. & Yan, P. ZBTB16 Overexpression Enhances White Adipogenesis and Induces Brown-Like Adipocyte Formation of Bovine White Intramuscular Preadipocytes. *Cell. Physiol. Biochem.* **48**, 2528–2538 (2018).
50. Beak, J. Y., Kang, H. S., Kim, Y.-S. & Jetten, A. M. Krüppel-like zinc finger protein Glis3 promotes osteoblast differentiation by regulating FGF18 expression. *J. Bone Miner. Res.* **22**, 1234–1244 (2007).
51. Banerjee, S. S. *et al.* The Krüppel-like factor KLF2 inhibits peroxisome proliferator-activated receptor-gamma expression and adipogenesis. *J. Biol. Chem.* **278**, 2581–2584 (2003).
52. Lombardi, R. *et al.* Cardiac Fibro-Adipocyte Progenitors Express Desmosome Proteins and Preferentially Differentiate to Adipocytes Upon Deletion of the Desmoplakin Gene. *Circ. Res.* **119**, 41–54 (2016).
53. Xu, T. *et al.* Compound and digenic heterozygosity contributes to arrhythmogenic right ventricular cardiomyopathy. *J. Am. Coll. Cardiol.* **55**, 587–597 (2010).
54. Knight-Schrijver, V. R. *et al.* A single-cell comparison of adult and fetal human epicardium defines the age-associated changes in epicardial activity. *Nat Cardiovasc Res* **1**, 1215–1229 (2022).
55. Streef, T. J. *et al.* Single-cell analysis of human fetal epicardium reveals its cellular composition and identifies CRIP1 as a modulator of EMT. *Stem Cell Reports* **18**, 1421–1435 (2023).
56. Meier, A. B. *et al.* Epicardioid single-cell genomics uncovers principles of human epicardium biology in heart development and disease. *Nat. Biotechnol.* **41**, 1787–1800 (2023).
57. Cai, C.-L. *et al.* A myocardial lineage derives from Tbx18 epicardial cells. *Nature* **454**, 104–108 (2008).
58. Zhou, B. *et al.* Epicardial progenitors contribute to the cardiomyocyte lineage in the developing heart. *Nature* **454**, 109–113 (2008).
59. Smart, N. *et al.* De novo cardiomyocytes from within the activated adult heart after injury. *Nature* **474**, 640–644 (2011).
60. Hesse, J. *et al.* Single-cell transcriptomics defines heterogeneity of epicardial cells and fibroblasts within the infarcted murine heart. *Elife* **10**, e65921(2021).
61. Eroglu, E. *et al.* Epicardium-derived cells organize through tight junctions to replenish cardiac muscle in salamanders. *Nat. Cell Biol.* **24**, 645–658 (2022).
62. Basha, S., Jin-Smith, B., Sun, C. & Pi, L. The SLIT/ROBO Pathway in Liver Fibrosis and Cancer. *Biomolecules* **13**, 785 (2023).
63. Feng, L. *et al.* Role of the SLIT-ROBO signaling pathway in renal pathophysiology and various

renal diseases. *Front. Physiol.* **14**, 1226341 (2023).

64. Liu, Y. *et al.* Crosstalk between the activated Slit2-Robo1 pathway and TGF-β1 signalling promotes cardiac fibrosis. *ESC Heart Fail* **8**, 447–460 (2021).
65. Katz, T. C. *et al.* Distinct compartments of the proepicardial organ give rise to coronary vascular endothelial cells. *Dev. Cell* **22**, 639–650 (2012).
66. Peacock, J. D., Lu, Y., Koch, M., Kadler, K. E. & Lincoln, J. Temporal and spatial expression of collagens during murine atrioventricular heart valve development and maintenance. *Dev. Dyn.* **237**, 3051–3058 (2008).
67. Braun, E. *et al.* Comprehensive cell atlas of the first-trimester developing human brain. *Science* **382**, eadf1226 (2023).
68. Betters, E., Liu, Y., Kjaeldgaard, A., Sundström, E. & García-Castro, M. I. Analysis of early human neural crest development. *Dev. Biol.* **344**, 578–592 (2010).
69. O'Rahilly, R. & Müller, F. The development of the neural crest in the human. *J. Anat.* **211**, 335–351 (2007).
