# Supplementary material for: Spatiotemporal gene expression and cellular dynamics of the developing human heart
Source: Nat Genet. 2025 Oct 29;57(11):2756–71. doi: 10.1038/s41588-025-02352-6 (PMC12597827; doi:10.1038/s41588-025-02352-6)
Supplement: Supplementary file 2 — Reporting Summary [file 41588_2025_2352_MOESM2_ESM.pdf]

nature research

Corresponding author(s): Joakim Lundeberg, Enikő Lázár

Last updated by author(s): Jul 21, 2025

Reporting Summary

Nature Research wishes to improve the reproducibility of the work that we publish. This form provides structure for consistency and transparency in reporting. For further information on Nature Research policies, see our Editorial Policies and the Editorial Policy Checklist.

Statistics

For all statistical analyses, confirm that the following items are present in the figure legend, table legend, main text, or Methods section.

n/a	Confirmed	
☐	☒	The exact sample size (*n*) for each experimental group/condition, given as a discrete number and unit of measurement
☐	☒	A statement on whether measurements were taken from distinct samples or whether the same sample was measured repeatedly
☐	☒	The statistical test(s) used AND whether they are one- or two-sided<br>*Only common tests should be described solely by name; describe more complex techniques in the Methods section.*
☒	☐	A description of all covariates tested
☒	☐	A description of any assumptions or corrections, such as tests of normality and adjustment for multiple comparisons
☐	☒	A full description of the statistical parameters including central tendency (e.g. means) or other basic estimates (e.g. regression coefficient) AND variation (e.g. standard deviation) or associated estimates of uncertainty (e.g. confidence intervals)
☐	☒	For null hypothesis testing, the test statistic (e.g. *F*, *t*, *r*) with confidence intervals, effect sizes, degrees of freedom and *P* value noted<br>*Give P values as exact values whenever suitable.*
☒	☐	For Bayesian analysis, information on the choice of priors and Markov chain Monte Carlo settings
☒	☐	For hierarchical and complex designs, identification of the appropriate level for tests and full reporting of outcomes
☒	☐	Estimates of effect sizes (e.g. Cohen's *d*, Pearson's *r*), indicating how they were calculated

Our web collection on statistics for biologists contains articles on many of the points above.

Software and code

Policy information about availability of computer code

Data collection	Sequencing data from the embryonic and fetal heart spatial samples was processed with 10x Genomics Space Ranger version 1.2.1.<br>Sequencing data from the embryonic and fetal heart single-cell samples was processed with 10x Genomics Cell Ranger version 4.0.0.
Data analysis	Data were analyzed on the statistical software R, with some scripts ran with Python, and some analyses performed on Excel. All analysis scripts and custom code are available at the public GitHub page: https://github.com/rmauron/HDCA_heart_dev.

For manuscripts utilizing custom algorithms or software that are central to the research but not yet described in published literature, software must be made available to editors and reviewers. We strongly encourage code deposition in a community repository (e.g. GitHub). See the Nature Research guidelines for submitting code & software for further information.

Data

Policy information about availability of data

All manuscripts must include a data availability statement. This statement should provide the following information, where applicable:

- Accession codes, unique identifiers, or web links for publicly available datasets
- A list of figures that have associated raw data
- A description of any restrictions on data availability

All the data required to replicate the analysis, including cellranger output, spaceranger output, metadata, processed ISS data, extended figures and tables, as well as main RDS objects are shared on the Mendeley DATA repository (links shared in the manuscript). The processed data of Visium, scRNA-seq and ISS are publicly available for browsing gene expression, clusterings, and other analysis results at https://hdcaheart.serve.scilifelab.se/web/index.html. The raw sequencing data can be shared upon reasonable request to the corresponding authors through EGA.

Field-specific reporting

Please select the one below that is the best fit for your research. If you are not sure, read the appropriate sections before making your selection.

☒ Life sciences ☐ Behavioural & social sciences ☐ Ecological, evolutionary & environmental sciences

For a reference copy of the document with all sections, see nature.com/documents/nr-reporting-summary-flat.pdf

Life sciences study design

All studies must disclose on these points even when the disclosure is negative.

Sample size	No sample-size calculation was performed. A close-to-even number of samples were included in the single-cell RNA-sequencing and Visium datasets (15 and 16 hearts, respectively), providing balanced coverage of the overlapping investigated developmental windows (5.5th-14th pcw for the single-cell RNA-sequencing and 6th-12th pcw for the Visium analysis). Age-resolved analysis of both datasets was performed with having at least three independent hearts in each compared age group. In the Visium analysis, two to four close-to-consecutive sections were processed, approximating technical replicates. For temporal comparison of single-cell cluster distributions, age-resolved populations were randomly downsampled to the size of the least abundandt group. The ISS dataset was generated from four hearts reflecting the age groups compared in the single-cell and Visium analyses, while supporting immunostaining was perfomed on three hearts and images were included in the manuscript from a single fetal heart sample.
Data exclusions	In the Visium analysis, we filter out low-count measurements and measurements outside the tissue. This is done for two reasons: first, it allows us to crop the data volume to the tissue area, thus reducing the size of the data and speeding up computing steps. Second, while there should be no gene expression outside the tissue, measurements may, in practice, be non-zero due to technical errors and diffusion. Therefore, we instead introduce a virtual measurement of the area outside the tissue which is forced to zero. These preprocessing steps were pre-established. The resulting count matrix was filtered for MALAT1, ribosomal, mitochondrial and hemoglobin genes.<br>In the single-cell analysis, cells with low counts were excluded. Additionally, cells with more than 30% mitochondrial transcript counts, less than 3% ribosomal transcripts, or more than 10% hemoglobin transcripts were removed.
Replication	Replication is attempted with close-to-consecutive sections for the Visium, ISS and immunostaining datasets, as described in the Methods section of the manuscript.
Randomization	Not applicable (no experimental groups)
Blinding	Not applicable (no experimental groups)

Reporting for specific materials, systems and methods

We require information from authors about some types of materials, experimental systems and methods used in many studies. Here, indicate whether each material, system or method listed is relevant to your study. If you are not sure if a list item applies to your research, read the appropriate section before selecting a response.

Materials & experimental systems

n/a	Involved in the study	
☐	☒	Antibodies
☒	☐	Eukaryotic cell lines
☒	☐	Palaeontology and archaeology
☒	☐	Animals and other organisms
☐	☒	Human research participants
☒	☐	Clinical data
☒	☐	Dual use research of concern

Methods

n/a	Involved in the study	
☒	☐	ChIP-seq
☒	☐	Flow cytometry
☒	☐	MRI-based neuroimaging

Antibodies

Antibodies used	anti-ARL13B (RRID:AB_3073658, ab136648, Abcam, 1:400), anti-PDE4C (RRID:AB_3094595, HPA054218, Atlas Antibodies, 1:100), anti-ATF3 (RRID:AB_1078233, HPA001562, Atlas Antibodies, 1:100)
Validation	All the utilized antibodies have been validated for IF application in human cells or tissues, according to the product documentation.

Human research participants

Policy information about studies involving human research participants

Population characteristics	Gender and age of each sample included in the single-cell and Visium datasets is described in the manuscript. Although not

Population characteristics	chosen, sex distribution in the study was balanced (17 hearts from female and 14 from male donors). Sex of the donors included in the ISS dataset and IHC images was not investigated. Samples collected are all from the developmental period between 5.5th-14th postconceptional weeks (spatial datasets: 6th-12th pcw; single-cell dataset: 5.5th-14th pcw).
Recruitment	All heart specimens included in this study were collected from elective medical abortions at the Department of Obstetrics and Gynecology at Danderyd Hospital and Karolinska Huddinge Hospital in Stockholm, Sweden. Only individuals over 18 years old with full decision-making capacity and without diagnosed psychiatric conditions affecting consent were eligible to donate embryonic tissues. The patients donated tissue with written informed consent, after receiving both oral and written information about the purpose of the research project, and the possibility of retracting their consent at any time, including later destruction of the donated tissue. The study participants did not receive any kind of compensation for their donation.
Ethics oversight	The study was performed with approval of the Swedish Ethical Review Authority and the National Board of Health and Welfare, under the ethical permit number 2018/769-31, in accordance with Swedish regulations governing the use of prenatal tissue for medical research and treatment.

Note that full information on the approval of the study protocol must also be provided in the manuscript.
